# Structural variation and eQTL analysis in two experimental populations of chickens divergently selected for feather-pecking behavior

**DOI:** 10.1007/s10048-022-00705-5

**Published:** 2022-11-30

**Authors:** Clemens Falker-Gieske, Jörn Bennewitz, Jens Tetens

**Affiliations:** 1 Department of Animal Sciences, Georg-August-University, Burckhardtweg 2, 37077 Göttingen, Germany; 2 Institute of Animal Science, University of Hohenheim, Garbenstr. 17, 70599 Stuttgart, Germany; 3 Center for Integrated Breeding Research, Georg-August-University, Albrecht-Thaer-Weg 3, 37075 Göttingen, Germany

**Keywords:** Feather pecking, GABA, Structural variation, Schizophrenia, Genome-wide association study, eQTL

## Abstract

Feather pecking (FP) is a damaging nonaggressive behavior in laying hens with a heritable component. Its occurrence has been linked to the immune system, the circadian clock, and foraging behavior. Furthermore, dysregulation of miRNA biogenesis, disturbance of the gamma-aminobutyric acid (GABAergic) system, as well as neurodevelopmental deficiencies are currently under debate as factors influencing the propensity for FP behavior. Past studies, which focused on the dissection of the genetic factors involved in FP, relied on single nucleotide polymorphisms (SNPs) and short insertions and deletions < 50 bp (InDels). These variant classes only represent a certain fraction of the genetic variation of an organism. Hence, we reanalyzed whole-genome sequencing data from two experimental populations, which have been divergently selected for FP behavior for over more than 15 generations, performed variant calling for structural variants (SVs) as well as tandem repeats (TRs), and jointly analyzed the data with SNPs and InDels. Genotype imputation and subsequent genome-wide association studies, in combination with expression quantitative trait loci analysis, led to the discovery of multiple variants influencing the GABAergic system. These include a significantly associated TR downstream of the GABA receptor subunit beta-3 (*GABRB3*) gene, two microRNAs targeting several GABA receptor genes, and dystrophin (*DMD*), a direct regulator of GABA receptor clustering. Furthermore, we found the transcription factor *ETV1* to be associated with the differential expression of 23 genes, which points toward a role of *ETV1*, together with *SMAD4* and *KLF14*, in the disturbed neurodevelopment of high-feather pecking chickens.

**Supplementary Information:**

The online version contains supplementary material available at 10.1007/s10048-022-00705-5.

## Introduction

FP in chickens is a behavioral disorder that severely impacts animal welfare and causes significant economic losses. It has been proposed that FP is obsessive–compulsive-like behavior [3]. In the past, the damage has been controlled by beak trimming, which has now been prohibited in many European countries. Numerous studies found an involvement of environmental factors such as light intensity, nutrition, stocking density, and lack of foraging (reviewed by [4] and [5]). Furthermore, evidence has been accumulating that the immune system plays a major role in the development of FP behavior [1, 6–8]. The gut microbiota has also been proposed to be involved in FP behavior, but this has been disproven in several studies [9, 10]. Since feather pecking is a complex heritable trait (reviewed by [11]), the dissection of the genetic causes is essential for the development of effective breeding strategies to eradicate the causative alleles. To achieve that goal, chickens were divergently selected for FP behavior over more than 15 generations based on their estimated breeding values for the behavior. Breeding of these lines was initiated in Denmark and continued in Hohenheim, Germany. In Hohenheim, two populations were established—an F_2_ cross and 12 half-sib families (in the preceding text referred to as F_2_ and HS). A detailed description of the experimental populations and the research conducted with them was reviewed by Bennewitz and Tetens [12]. Based on the results that were acquired by whole-genome and transcriptome sequencing with the Hohenheim selection lines, FP appears to be a disorder of the γ-aminobutyric acid (GABAergic) system in conjunction with a disturbance of embryonic neurodevelopment by a lack of leukocytes in the developing brain. Several variants in or in close proximity to GABA receptor genes were identified in genome-wide association studies (GWAS) conducted on medium-density single nucleotide polymorphism (SNP) array and imputed-sequence level genotypes [2, 13, 14]. Furthermore, brain transcriptome analysis of high and low feather peckers (HFP and LFP) before and after light stimulation revealed that HFP responds with very few changes in gene expression in comparison to LFP, with numerous GABA receptor genes upregulated in LFP. Only *GABRB2* (gamma-aminobutyric acid type A receptor subunit beta2) was among differentially expressed genes (DEGs) in HFP, but it was downregulated, instead of upregulated, a pattern that we observed for most DEGs in HFP brains [14]. We attribute this low level of gene expression changes in response to light to a high level of excitation in HFP brains due to the lack of multiple GABA receptors. Since GABA is the major inhibitory neurotransmitter, a lack of its receptors in the brain would lead to a high neuronal excitatory state, which explains the observed hyperactivity and the obsessive–compulsive-like behavior observed in HFP. Since *Dicer1* was among the downregulated DEGs, we assume that miRNA processing is disturbed, as is also the case in schizophrenia patients [15], which in turn leads to low GABA receptor expression levels. In the general comparison of brain transcriptomes between HFP and LFP hens, we observed an enrichment of immune system-related DEGs [1], which we could further pinpoint in an expression quantitative trait loci (eQTL) analysis to a small deletion 652 bp downstream of the *KLF14* gene. In total, the differential expression of 40 genes between HFP and LFP chickens was significantly associated with this *KLF14* variant, a majority of which are involved in leukocyte biology [8]. It has been shown in mice that CD4 T cells are essential for healthy development from the fetal to the adult brain. A defect in CD4 T cell maturation affected synapse development and led to behavioral abnormalities [16]. The evidence suggests that this mechanism is responsible for disturbing embryonic brain development in HFP chickens, which contributes to FP behavior.

One commonality of all the studies that we conducted on the genetics involved in FP is the overlap in associated genes with human psychiatric disorders, most prevalent schizophrenia. SVs play a notable role in human psychiatric disorders [17–19], which is also the case for tandem repeats (TRs) [20]. Commonly investigated classes of SVs include insertions, deletions, duplications, inversions, and translocations, which arise from various combinations of DNA gains, losses, or rearrangements [21]. Here, we present the first in-depth study on the potential role of SVs and TRs in FP, which led to a deeper understanding of the mechanisms responsible for this behavioral disorder.

## Material and methods

### Animals and husbandry

The F_2_ and HS lines of White Leghorn chickens were divergently selected for feather pecking behavior for over more than 15 generations at Hohenheim University [22, 23]. Animals, which were used for genotyping in the study presented here were as follows: from the F_2_ design 25 founder (whole-genome sequenced), 89 F_1_ (SNP chip genotyped), and 817 F_2_ animals (SNP chip genotyped) and from the HS population, 24 animals were whole-genome sequenced and 494 animals were SNP chip genotyped. RNA from the whole brains of 167 HS chickens was used for Fluidigm gene expression analysis for the eGWAS approach [8]. All experimental procedures [1], rearing and husbandry conditions [24], as well as phenotyping [2], were described in previous studies. Briefly, the phenotypic data were generated by direct observations made by seven independent investigators at approximately 32 weeks of age. Observations were recorded in 20 min sessions in average group sizes of about 42 animals. The phenotypes are expressed as the number of FP bouts actively delivered during a standardized time span. As these count data are heavily distributed from normality, Box–Cox-transformation was applied [2, 13].

### Structural variants and short tandem repeats discovery

Illumina whole genome sequencing data were mapped to chicken genome version GRCg6a (GCF_000002315.5 RefSeq assembly) and used to call SNPs and short (< 50 bp) insertions and deletions (InDels) in our previous study [2]. This was achieved with the genome analysis toolkit (GATK) v. 4.0, according to the best practice guideline of the broad institute [25]. SVs were called as described by Blaj et al. [26] with slightly different settings: A high-confidence SV call set was produced from the output of three variant callers: smove v. 0.2.6 (Brent, P. (2018) Smoove. https://brentp.github.io/post/smoove/), DELLY v. 0.7.7 [27], and manta v. 1.6.0 [28]. SURVIROR v. 1.0.7 [29] was used to combine the output of the three variant callers with the following settings: maximum distance between breakpoints of 1000 bp, minimum number of supporting callers 2, SV type and strands were taken into account, and the minimum SV size was set to 30 bp. Variants with a call rate < 0.8 and variants with QUAL < 1000 were removed. TRs were called with GangSTR v. 2.5 [30]. A library of known TRs as input for GangSTR was acquired from the UCSC data repository (https://hgdownload.soe.ucsc.edu/goldenPath/galGal6/bigZips/). TRs were filtered to retain genotypes with a minimum sequence depth (DP) of 10, a quality score (Q) higher than 0.8, and a call rate < 0.8.

### Haplotype construction and imputation

To impute medium-density chip genotypes to whole genome-level SNPs and InDels, we employed the same strategy as we previously described [2], with the deviation that all imputation steps were performed with Beagle v. 5.2 [31] and the setting ne = 1000. SVs and TRs were merged with SNPs and InDels from our previous study, which were acquired with the GATK [25]. This merged call set was phased with Beagle v. 5.2 for the estimation of haplotypes and used as a reference panel to impute medium-density chip genotypes to SVs and TRs with the same strategy as for SNPs and InDels. Chip genotypes from HS animals were directly imputed using the WGS reference panel. For the F_2_ design, we first imputed chip-genotyped F_1_ animals to the WGS level, merged the output with the initial reference panel, phased the merged dataset, and used it as a reference panel for the imputation of F_2_ SNP chip-genotyped animals. To remove SNPs and InDels from the imputed SVs/TRs, GATK SelectVariants was utilized.

### Detection of quantitative trait loci

Prior to GWAS, multiallelic variants from the SVs/TR dataset were converted to biallelic variants with the norm function from bcftools v. 1.14 [32]. All GWAS were conducted with gcta v. 1.92.3 beta3 [33], applying a mixed linear model association analysis with a leaving-one-chromosome-out (LOCO) approach and a minor allele frequency (MAF) threshold of 0.01. In brief, relatedness between animals and stratification [6] was corrected by including a random genetic term based on a genomic relationship matrix calculated only from SNP-chip data and following a LOCO approach [34]. Briefly, a model of the form $y=W\alpha +X\beta +u+\epsilon$ *is* fitted (*y* = *n* × 1 vector of phenotyped (n) hens; *W* = *n* × *c* incidence matrix of fixed effects with *c* being the number of effects; *α* = vector of corresponding coefficients including the mean; *X* = *n* × 1 vector of marker genotypes at the locus tested; *β* = corresponding effect size; *u* = vector of random genetic effects, with $u\sim N(0,{A}^{-}{\sigma }_{g}^{2})$, where ${\sigma }_{g}^{2}$ represents genetic variance and *A*^−^ is the genomic relationship matrix based on all SNP-chip markers except those on the chromosome currently analyzed; ε = random residual term, with $\epsilon \sim N(0,I{\sigma }_{\epsilon }^{2})$, where ${\sigma }_{\epsilon }^{2}$ represents the residual variance and *I* represents an identity matrix). Line effects (HFP and LFP) and hatch were included in the analysis of the HS design; for the F_2_ design, only the hatch was included as a fixed effect. The phenotype used for GWAS, “*feather pecks delivered Box–Cox transformed*” (FPD_BC), has been described by Iffland et al. [13]. Phenotypes for expression GWAS (eGWAS) were normalized gene expression data from our previous study [8]. There we analyzed the expression of 86 genes in 167 HS chickens, which we discovered in a transcriptome analysis performed on 48 of the HS birds [1]. Information on the hatch was used as a covariate in all GWAS and eGWAS. Genomic relationship matrices were created from the target 60 k SNP Chip genotypes. Meta-analyses of GWAS results were performed with METAL v. 1.1 [35] using the sample size-based approach with default settings. The proportion of variance in phenotype explained by a given SNP (PVE) was calculated according to the formula Var(*Y*) = *β*^2^Var(*X*) + *σ*^*2*^ by Shim et al. [36] (Var(*Y*) = variance in phenotype; *β* = effect size of genetic variant *X*; *σ*^*2*^ = remaining variance). To correct for multiple testing, the threshold for genome-wide significance of variants was calculated by Bonferroni correction ($\frac{\mathrm{number}\;\mathrm{of}\;\mathrm{variants}}{0.05}$).

### Association weight matrix construction

The AWM was created as described in our previous study [8] by deploying the strategy for AWM construction by Reverter and Fortes [37], followed by the detection of significant gene–gene interactions with their PCIT algorithm [38]. Input variants were chosen as follows: *p*-value < 1 × 10^−4^ for the main phenotype (FPD_BC) or *p*-value < 1 × 10^−4^ in at least ten of the eGWAS. That way, variants affecting the main phenotype and gene expression were both considered in the analysis. This led to the selection of 57 input variants, 0.16% of all detected SVs and TRs, for the HS population. The R script by Reverter and Fortes was modified by setting the *p*-value threshold for primary and secondary SNP selection to 1 × 10^−4^. The gene–gene interaction map was constructed with Cytoscape [39], and gene class information was acquired with PANTHER [40] and UniProt [41].

### Transcription factor enrichment

To detect significant binding site enrichment for the transcription factor *ETV1*, the CiiiDER software (build May 15th, 2020) [42] was employed with frequency matrix MA0761.2 (https://jaspar.genereg.net/matrix/MA0761.2/) and the following settings: *p*-value threshold for gene coverage enrichment = 0.05; base position upstream scan limit = 1500 bp; base position downstream scan limit = 500 bp. Additional members of the ETS (E twenty-six) family of transcription factors used in the analysis were *ETV2* (MA0762.1), *ETV3* (MA0763.1), *ETV4* (MA0764.2), *ETV5* (MA0765.1), *ETV6* (MA0645.1), and *ETV7* (MA1708.1). Genes that were associated with the *ETV1* variant (*p*-value < 1 × 10^−4^; Supplementary Information S1) were used as input, and DEGs with a log_2_ fold change < 0.2 were used as background genes (14,514 genes).

## Results

### Genome-wide association studies with different classes of genetic variants

**Fig. 1:**
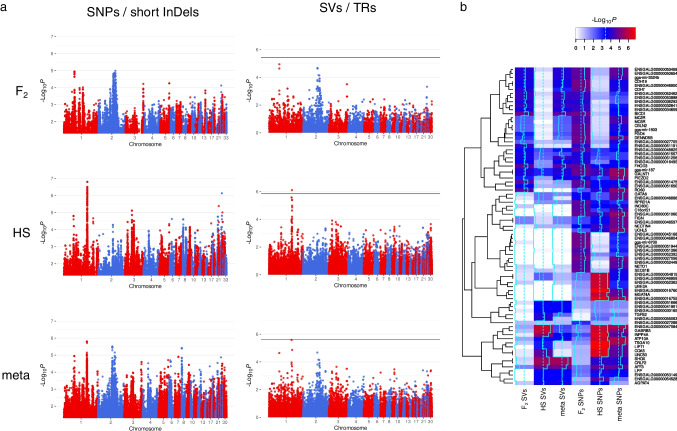
**a** Genome-wide association studies (GWAS) performed with single nucleotide polymorphisms (SNPs) in combination with short (< 50 bp) insertions and deletions (InDels) and structural variants (SVs) in combination with tandem repeats (TRs). All variant classes were investigated in the F_2_ cross and half-sib (HS) families of chickens divergently selected for feather pecking and subsequently combined in a meta-analysis. **b** Heatmap of the 20 highest associated genes from each GWAS. The – log_10_ *p*-values of the highest associated variants for each gene were used

In total, 63,824 TRs and 11,098 SVs were discovered in the joint variant calling of whole genome sequenced HS and F_0_ chickens. The SVs contained 6014 deletions, 2741 inversions, 1334 duplications, and 995 translocations. The variant calling of SNPs and InDels was reported in our previous study and yielded 12,864,421 SNPs and 2,142,539 InDels [2]. To grasp the whole depth of the datasets at hand, we repeated the imputation of SNPs and InDels with the most recent Beagle version (v. 5.2) [31] and set the effective population size to 1000, which is known to improve the imputation accuracy in small populations [43]. GWAS results for the trait FPD_BC were analyzed for both experimental designs, the F_2_ cross, and the HS population, separately and after combining the results in a meta-analysis. Manhattan plots from GWAS with imputed SNPs/InDels and imputed SVs/TRs are shown in Fig. 1a. Common peaks for both variant classes on GGA1 and GGA2 were observable in the F_2_ cross, as well as on GGA1 and GGA3 of the HS population. QTL are summarized in Table 1, and lead SVs and TRs with their predicted effects are listed in Table 2.

**Table 1:** Positional ranges in base pairs of QTL that were discovered in both experimental populations (F_2_ cross and half-sib (HS) families) for both groups of variant classes: single nucleotide polymorphisms (SNPs) and short insertions/deletions (InDels) as well as structural variants (SVs) and tandem repeats (TRs)

F_2_ SNPs and short InDels	F_2_ SVs and TRs	HS SNPs and short InDels	HS SVs and TRs
GGA1: 59,032,772–59,548,358	GGA1: 59,138,261–59,216,858	GGA1: 132,578,705–133,803,185	GGA1: 130,621,356–134,077,752
GGA2: 81,060,432–97,072,903	GGA2: 83,493,152–94,489,210	GGA3: 18,434,015–54,601,143	GGA3: 19,120,538–70,385,553

**Table 2:** Structural variants (inversions (INV) and deletions (DEL)) and tandem repeats (TRs) that showed the highest association in genome wide association studies for feather pecking behavior in an F_2_ cross and half-sib (HS) families. Variant effects and closest genes were predicted with SnpEff

GWAS	Type	Size (bp)	Position	*p*-value	Effect	Gene(s)
F_2_	INV	1403	GGA1: 59,216,858–59,218,260	1.14 × 10^−5^	intron_variant	*BICD1*
F_2_	TR		GGA1: 59,138,261	2.29 × 10^−5^	intron_variant	*BICD1*
F_2_	TR		GGA2: 83,217,700	2.11 × 10^−5^	intron_variant	*FHOD3*
F_2_	DEL	509	GGA2: 83,493,152–83,493,661	2.11 × 10^−5^	intergenic_region	*ENSGALG00000051475-RPRD1A*
F_2_	TR		GGA2: 83,497,153	2.30 × 10^−5^	intergenic_region	*ENSGALG00000051475-RPRD1A*
F_2_	DEL	209	GGA2: 83,497,939–83,498,148	2.30 × 10^−5^	intergenic_region	*ENSGALG00000051475-RPRD1A*
F_2_	TR		GGA2: 89,486,485	6.90 × 10^−5^	intergenic_region	*ENSGALG00000053898-ENSGALG00000052462*
F_2_	TR		GGA2: 94,489,210	7.28 × 10^−5^	intergenic_region	*ENSGALG00000051191-ENSGALG00000032841*
HS	TR		GGA1: 132,889,302	7.79 × 10^−7^	intergenic_region	*GABRB3-ENSGALG00000047084*
HS	TR		GGA1: 130,621,356	2.64 × 10^−6^	intergenic_region	*CRLF2-SHOX*
HS	TR		GGA1: 133,533,130	4.00 × 10^−6^	intron_variant	*INPP4A*
HS	TR		GGA1: 133,533,741	4.09 × 10^−6^	intron_variant	*INPP4A*
HS	DEL	368	GGA1: 134,077,752–134,077,908	1.37 × 10^−5^	intron_variant	*AFF3*
HS	TR		GGA1: 133,606,912	2.30 × 10^−5^	downstream_gene_variant	*COA5*
HS	TR		GGA1: 132,889,302	6.42 × 10^−5^	intergenic_region	*GABRB3-ENSGALG00000047084*
HS	TR		GGA1: 134,020,467	9.02 × 10^−5^	intron_variant	*AFF3*
HS	TR		GGA3: 19,445,704	0.00012	downstream_gene_variant	*TGFB2*
HS	TR		GGA3: 40,921,527	0.00016	intron_variant	*DLL1*
HS	TR		GGA3: 19,264,158	0.00022	intergenic_region	*LYPLAL1-ENSGALG00000051126*
HS	TR		GGA3: 45,338,399	0.00023	intron_variant	*AGPAT4*
HS	DEL	531	GGA3: 54,243,436–54,244,326	0.00026	intron_variant	*REPS1*
HS	TR		GGA3: 70,359,487	0.00032	intron_variant	*GRIK2*
HS	TR		GGA3: 70,385,553	0.00032	intron_variant	*GRIK2*
HS	TR		GGA3: 19,120,538	0.00049	intergenic_region	*ENSGALG00000047985-LYPLAL1*

The only variant that reached genome-wide significance after Bonferroni correction (−*p* < 1.41 × 10^−6^) was a TR in the HS population, 126,821 bp downstream of *GABRB3*. The proportion of variance in the phenotype FPD_BC that is explained by this variant is 0.047. Given heritability estimates of around 0.15 [22, 44, 45], the amount of phenotypic variance explained by the lead variant is considerable. To visualize overlaps between the six different sets of results, we created a heat map, which shows the − log_10_*p*-values of the top 20 associated genes from the different GWAS (Fig. 1b) and summarized all *p*-values in Supplementary Information S2. Numerous genes showed high association signals (*p* < 0.01) in the meta-analyses for both variant classes: *AFF3*, *ATP10A*, *BICD1*, *CDH19*, *CDH7*, *CRLF2*, *ENSGALG00000016495* (*KLHL29*), *ENSGALG00000032841* (lncRNA), *ENSGALG00000034659* (miRNA), *ENSGALG00000046900* (lncRNA), *ENSGALG00000048825* (lncRNA), *ENSGALG00000050654* (lncRNA), *ENSGALG00000053149* (miRNA), *ENSGALG00000053459* (lncRNA), *FHOD3*, *GALNT1*, *gga-mir-187*, *gga-mir-3524b*, *INPP4A*, *LPP*, *PIEZO2*, and *SHOX*. We assume that these genes are burdened with multiple mutations that contribute to the phenotype, which might be a result of the divergent selection for feather-pecking behavior over multiple generations. Predicted targets of the microRNA (miRNA) *gga-mir-187* are *GABRA1*, *GABRB2*, *GABRB3*, *GABRG1*, and *GABRG2*. *GABRB2* is also a predicted target of *gga-mir-3524b* (www.targetscan.org, accessed January 27, 2022).

### Expression quantitative trait loci (eQTL) analyses

To clarify whether the SVs and TRs that we detected influence the expression of transcripts that we identified in a previous study to be differentially expressed between LFP and HFP [1], we performed an eQTL analysis. We employed the same strategy as in our past study [8] and performed eGWAS for 86 genes from 167 chickens (84 HFP and 83 LFP) from the HS population (Manhattan plots are summarized in Supplementary Information S3). A total of 35,571 SVs and TRs were screened for association with gene expression, and we detected 909 genome-wide significant associated signals. SVs and TRs for which we detected genome-wide association with at least 10 DEGs are shown in Table 3. To identify significant gene–gene interactions from those 86 eGWAS, we constructed an association weight matrix [37], followed by the detection of significant correlations with the PCIT algorithm [38]. Central to the gene–gene interaction map is the transcription factor *ETV1*. We selected DEGs with *p*-values < 1 × 10^−4^ for association with the variant, a tandem repeat with the sequence CCCGGCCCG 70 bp upstream of *ETV1* (GGA2: 27,337,541). This led to the selection of 23 genes that were associated with this variant, with *p*-values ranging from 1.41 × 10^−6^ to 9.89 × 10^−5^ with the top associated DEG CERS4L almost reaching genome-wide significance (*p*-value = 1.41 × 10^−6^). With the 23 selected genes (Supplementary Information S1), we performed a transcription factor binding site enrichment analysis with Ciiider and found a significant enrichment (*p*-value = 0.013, log_2_-enrichment = 0.494) of *ETV1* binding sites (Fig. 3a) in proximity to those genes (Fig. 3b, Supplementary Information S4). To demonstrate specificity, we included all available PWMs for members of the ETS family of transcription factors: *ETV2*–*ETV6*. The only other member of the TF family for which transcription factor binding site enrichment was detected was *ETV4*, but the *p*-value was not significant. The complete results of the analysis are summarized in Supplementary Information S5. Another noteworthy gene, which we discovered in an eQTL study with SNPs and InDels [8], is *dystrophin* (*DMD*). The variant at position GGA1: 116,965,973, which is a biallelic intronic TR between exons 7 and 8, was associated with genome-wide significance to the DEGs *LOC112531493* (*p*-value = 1.36 × 10^−8^), *LOC422393* (*p*-value = 5.57 × 10^−6^), *RASSF8* (*p*-value = 5.76 × 10^−6^), and *LOC112533169* (*p*-value = 8.48 × 10^−6^). The 2 bp *DMD* intron deletion between exons 1 and 2 we discovered in our previous study (rs735635304, GGA1: 117,179,438) was associated with genome-wide significance to the DEGs *LOC112532977* (*p*-value = 7.34 × 10^−10^) and *LOC107049114* (*p*-value = 1.60 × 10^−9^). The two DMD variants are 213 kb apart and not in linkage disequilibrium (*R*^2^ = 0.022868).

**Table 3:** Large deletions (DEL) and tandem repeats (TR) that showed genome-wide significant association (*p*-values < 1.41 × 10^−6^) in at least 10 expression genome-wide association studies for genes that are differentially expressed in brains of high and low feather pecking chickens

Type	Size (bp)	Position	Effect	Gene(s)	No. of associations
TR		GGA25: 1,465,942	intergenic_region	*ENSGALG00000055092–IQGAP3*	53
TR		GGA25: 3,317,155	intron_variant	*S100A16*	47
TR		GGA17: 6,359,797	intergenic_region	*ENSGALG00000035908–ENSGALG00000047338*	39
DEL	294	GGA8: 3,889,333–3,889,627	intron_variant	*RO60*	30
TR		GGA2: 56,803,419	intron_variant	*ATP9B*	22
TR		GGA2: 56,944,739	intergenic_region	*SALL3-ENSGALG00000054175*	22
TR		GGA17: 5,355,006	upstream_gene_variant	*SLC27A4*	15
TR		GGA27: 3,942,783	intron_variant	*GPATCH8*	11
TR		GGA27: 3,976,916	frameshift_variant	*GPATCH8*	11
TR		GGA27: 3,977,142	intron_variant	*GRN*	11
TR		GGA27: 3,988,533	intergenic_region	*TMEM98-ARHGAP27*	11
TR		GGA27: 4,009,079	intron_variant	*PLEKHM1*	11
TR		GGA27: 4,022,832	intron_variant	*ENSGALG00000047878*	11

## Discussion

Numerous studies have been conducted to unravel the genetics behind FP behavior in chickens, as reviewed in [12, 46], but none of these focused on the analysis of structural genetic variation. SVs contribute to complex traits to a higher degree than SNPs [21] and have been the focus of studies on neuropsychiatric disorders in recent years [17–19]. Here, we combined data from two well-described experimental crosses, an F_2_ design and a HS population, and performed multiple GWAS and eQTL analyses on different classes of genetic variants. By applying conventional GWAS approaches on the two experimental populations with two sets of variant classes (SNPs and InDels as well as SVs and TRs) followed by meta-analysis, we identified strong associations with numerous putative candidate genes for both variant classes. GALNT1 catalyzes glycosylation of target proteins, and aberrant glycosylation of i.e. the GABA_A_ receptor is a pathological hallmark in postmortem brains of patients suffering from schizophrenia [47]. FHOD3 controls dendritic spine morphology [48] and might contribute to FP behavior by causing aberrant neuronal development in the cerebral cortex. Similarly, *KLHL29* has already been discussed to be involved in a neurodevelopmental disorder [49]. *AFF3* is also a new promising candidate gene that was previously associated with intellectual disability and cellular migration in the cerebral cortex [50, 51]. Regarding its cellular function, PIEZO2 is of considerable interest. It is a mechanically activated cation channel, required for light/touch sensation and proprioception, and is abundantly expressed in dorsal root ganglion and sensory endings of proprioceptors in mice [52]. Since one of the major triggers of FP behavior is light stimulation [14, 53], genetic variation in *PIEZO2* might lead to disturbed light perception in HFP.

**Fig. 2:**
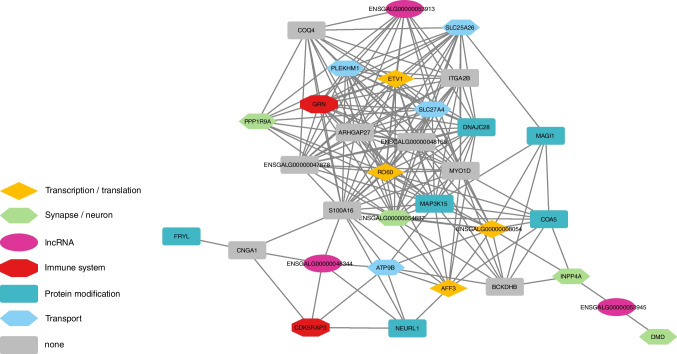
Gene–gene interaction map of significant correlations that were identified from an association weight matrix on expression genome-wide association studies with the PCIT algorithm

**Fig. 3:**
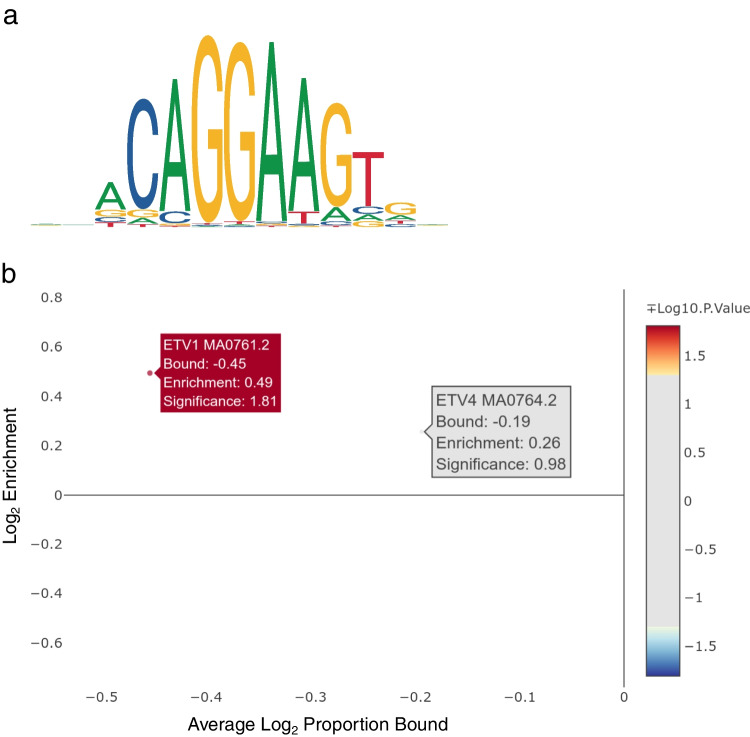
**a** Graphical representation of the binding motif frequency matrix of the *ETV1* transcription factor. **b** Result of transcription factor binding site enrichment analysis for *ETV1* with genes from expression genome-wide associations studies with *p*-values < 1 × 10^-4^

However, the lead variant from these analyses is a TR 126 kb downstream of the *GABRB3* gene, which explains a considerable amount of the observed phenotypic variance. But, since this variant was detected with a sample size of about 500 animals, its contribution to the phenotype should be interpreted with caution. According to the Beavis effect, the results of QTL studies with sample sizes of around 500 individuals are slightly overestimated [54]. Furthermore, miRNA *gga-mir-187* is strongly associated with FP behavior in all conducted GWAS (Fig. 1b), and among predicted targets of this regulatory RNA are several GABA receptor genes. Based on whole-brain transcriptome analyses of the light response of HFP, we previously postulated that downregulation or missing upregulation of GABA receptor expression is caused by miRNA dysregulation due to low levels of *Dicer1* expression in HFP brains [14]. The findings presented here provide further evidence for a disturbance in miRNA regulation and involvement of the GABAergic system in FP behavior. Since these chickens have been selected for FP behavior based on their estimated breeding values for multiple generations, it is possible that mutations leading to low expression of several GABA receptors have been accumulating. Previous studies with the same experimental population already pointed in that direction [2, 13]. But these are not only limited to GABA receptor genes, miRNAs, or genes encoding miRNA processing proteins. By conducting an eQTL analysis with SVs and TRs, we were able to confirm the involvement of *DMD* (dystrophin) in the regulation of genes that are differentially expressed between HFP and LFP. We already discovered *DMD* in an eQTL analysis with SNPs and InDels [8]. Since dystrophin is a direct regulator of GABA receptor clustering [55], this adds another layer to the GABA receptor disturbance in HFP brains. Several other genes that appear in the gene–gene interaction map based on significant AWM correlations (Fig. 2) have been connected to the GABAergic system. These include *COQ4* [56], *ETV1* [57], *NEURL1* [58], *SLC25A26*, and *SLC27A4* [59]. In this regard, *ETV1* is of considerable interest since it is the only transcription factor that we identified in the AWM analysis with SVs and TRs. According to the Human Protein Atlas, *ETV1* expression is specific to salivary glands and the brain, specifically the cerebellum and thalamus (https://www.proteinatlas.org/ENSG00000006468-ETV1; accessed August 2022). *ETV1* is associated with the differential expression of 23 genes between HFP and LFP, and *ETV1* binding sites are enriched in proximity to these genes (Fig. 3). Although genome-wide significance was closely missed for these associations, we strongly believe that the AWM approach by Reverter and Fortes [37] led to the discovery of a high-confidence set of variants for this complex trait. Among these putative ETV1 targets are *CSF2RB*, which has been associated with major depression and schizophrenia [60], and *MIS18BP1*, a gene implicated in the autism spectrum [61]. Furthermore, an *ETV1* antibody coimmunoprecipitated the GABAA receptor α6 (GABAARα6) promoter region in mice [57], and *ETV1* is a regulator of gene expression in CD4 and CD8 T cells [62]. Hence, *ETV1* may not only impact GABA receptor expression but might also participate in neurodevelopment. As demonstrated by Pasciuto *et al.*, a lack of CD4 T cells in the brain of mice leads to excess immature neuronal synapses and behavioral abnormalities. Among the top DEGs identified by single-cell RNAseq were the transcription factors *Klf2* and *Klf4* [16]. We previously postulated that FP behavior is the result of disturbances in embryonic neurodevelopment and identified *KLF14* as a major regulator in this regard [8]. In that study, we also identified a Smad4 domain-containing transcription factor, namely *ENSGALG00000042129* (*CHTOP*), but we were not able to detect the enrichment of binding sites for that transcription factor in proximity to its associated DEGs. However, in light of the fact that ETV1 forms functional complexes with Smad4 [63], we propose a model in which the transcription factors *ETV1*, *CHTOP*, and *KLF14* have an additive effect on the density of T cells in the developing brain of HFP. This might lead to structural abnormalities in the brains of HFP and may explain, at least in part, their abnormal behavior. Multiple studies point toward a central role of Krüppel-Like factors in neurodevelopment and behavior [64–68]. However, conclusive results on the function of Krüpple-like factors in the neurodevelopment in chickens have not been reported yet, and research is hampered by erroneous annotation of KLF orthologues [69].

Apart from *DMD*, we also discovered the genes *PPP1R9A*, *INPP4A*, and *COA5* in both eQTL-AWM analyses, one of which was performed with SVs and TRs (Fig. 2) and one with SNPs and InDels [8]. In schizophrenia and bipolar disorder, the prefrontal cortical expression of *PPP1R9A* was altered [70] and its gene product Neurabin regulates anxiety-like behavior in adult mice [71]. *INPP4A* has been implicated in multiple neurological conditions, namely schizophrenia, autism, epilepsy, and intellectual disability [72–74]. An additional link to schizophrenia is a frameshift variant in the *GPATCH8* gene in exon 9 of transcript GPATCH8-201 (Table 3), an ortholog of *ZNF804A*, which has been shown to impact various mental illnesses via pre-mRNA processing [75]. Multiple TRs and one large deletion in *RO60* showed genome-wide association with at least 30 DEGs between HFP and LFP (Table 3). With 53 genome-wide associations, an intergenic TR seemed to have an impact on more than half of the top DEGs between HFP and LFP whole brains. The variant is located 2.7 kb downstream of the E3 ubiquitin ligase *ENSGALG00000055092* (*DCAF11*) and 7.5 kb upstream of IQGAP3, which is required for proper cell cycle progression [76] and which influences immune cell infiltration and immune modulators [77]. A 294-bp intron deletion in *RO60*, a gene involved in the regulation of inflammatory gene expression [78], was associated with 30 DEGs, which might be responsible for the observation that 48.1% of DEGs between HFP and LFP belong to the PANTHER protein class defense/immunity (PC00090) [1]. A TR within an *S100A16* intron yielded 47 significantly associated DEGs, which is also a gene related to the immune system [79]. An intergenic TR with 39 genome-wide associated DEGs was located between the two lncRNAs *ENSGALG00000035908* and *ENSGALG00000047338*. They probably belong to the class of psychiatric ncRNAs, a term scored by Gandal *et al.* [80].

We discovered multiple links between FP and human psychiatric disorders in our previous studies [1, 2, 14]. The fact that methylation of *KLF14* correlates with psychosis severity in schizophrenia patients [81] strengthens our argument for the applicability of HFP chickens in the research of the basic mechanisms involved in human psychiatric disorders. Furthermore, by conducting multiple GWAS on two experimental populations of FP chickens with different variant classes with whole-genome marker density, we identified numerous genes that have previously been linked to psychiatric disorders or other neurological conditions or phenotypes (Table 4). These genes coherently fit the mechanistic hypotheses raised so far and, at the same time, underpin the complex nature of this trait. Among these are anxiety, depression, intellectual disability, autism, compulsive behavior, drug addiction, bipolar disorder, and schizophrenia. This raises the question of what common mechanisms these conditions share, which is a current matter of debate. Blokhin *et al.* proposed that future treatment of human psychiatric disorders should be tailored under the use of a genome-wide “targetome” [82], and Johnsson *et al.* discovered multiple genetic overlaps between anxiety behavior in chickens and numerous human psychiatric disorders [83]. The strong genetic burden of HFP chickens with mutations affecting neuropsychiatric genes makes this line of chickens a valid model system to study the effects of tailored drug treatment strategies.

**Table 4:** Summary of published data related to neurological disorders on the 20 highest associated genes from each genome wide association study on feather pecking behavior in laying hens

Gene	Gene product	Published data related to neurological disorders
*AFF3*	AF4/FMR2 family member 3	Association with intellectual disability [50, 51]
*AGPAT4*	1-acyl-sn-glycerol-3-phosphate acyltransferase delta	Age-dependent increase of anxiety in knockout mice [84] and association with the major depressive disorder [85]
*ATP10A*	Phospholipid-transporting ATPase VA	Autism susceptibility locus [86]
*CBLN2*	Cerebellin-2	Regulator of compulsive behavior [87]
*CDH7*	Cadherin-7	Associated with the major depressive disorder [88]
*DENND5B*	DENN domain-containing protein 5B	Altered expression in patients with epilepsy and regulation of seizures in mice [89]
*FGD4*	FYVE, RhoGEF and PH domain-containing protein 4	Involved in clustering and trafficking of GABA receptors [90]
*FHOD3*	FH1/FH2 domain-containing protein 3	Controls the dendritic spine morphology [48]
*GABRB3*	Gamma-aminobutyric acid receptor subunit beta-3	Associated with FP in chickens [2, 14] and schizophrenia[91]
*INO80C*	INO80 complex subunit C	Mediator of cocaine addiction [92]
*INPP4A*	Inositol polyphosphate-4-phosphatase type I A	Decreased expression in epilepsy [73] and a nonsense mutation in intellectual disability [72]
*KIAA1211L* (*ENSGALG00000016755*)	CRACD-like protein	Associated with depression, bipolar disorder, schizophrenia [93], and opioid use [94]
*LIPT1*	Lipoyltransferase 1	Involved in the development of Leigh syndrome [95]
*MC2R*	Adrenocorticotropic hormone receptor	Risk factor for schizophrenia [96]
*MGAT4A*	Alpha-1,3-mannosyl-glycoprotein 4-beta-N-acetylglucosaminyltransferase A	Abnormal expression in the prefrontal cortex in schizophrenia [97]
*MRPL30*	39S ribosomal protein L30	Associated with the major depressive disorder [85]
*NECTIN4*	Nectin-4	Associated with opioid abuse [98]
*NETO1*	Neuropilin and tolloid-like protein 1	NMDA receptor-interacting protein is required for synaptic plasticity and learning [99]
*SHOX*	Short stature homeobox protein	Autism locus [100]
*TGFB2*	Transforming growth factor beta-2 proprotein	Involved in signaling toward the age of onset and cognitive functioning in schizophrenia [101]
*TSGA10*	Testis-specific gene 10 protein	miRNA target in the development of schizophrenia [102]
*UBE3A*	Ubiquitin-protein ligase E3A	Autism susceptibility locus [86] [103]
*UNC50*	Protein unc-50 homolog	Associated with bipolar disorder [104] and involvement in the cell-surface expression of neuronal nicotinic receptors [105]

## Supplementary Information

Below is the link to the electronic supplementary material.

Supplementary file1 (XLSX 8 KB)

Supplementary file2 (XLSX 10 KB)

Supplementary file3 (PDF 10116 KB)

Supplementary file4 (PDF 52 KB)

Supplementary file5 (XLSX 6 KB)

## Data Availability

All methods applied here have been outlined in previous studies [1, 2, 8]. The raw RNA sequencing data have been deposited at the NCBI Sequence Read Archive (BioProject ID PRJNA656654) and the raw whole genome sequencing data as well (BioProject ID PRJNA664592).
