# Supplementary material for: Structural variation and eQTL analysis in two experimental populations of chickens divergently selected for feather-pecking behavior
Source: Neurogenetics. 2022 Nov 30;24(1):29–41. doi: 10.1007/s10048-022-00705-5 (PMC9823035; doi:10.1007/s10048-022-00705-5)
Supplement: Supplementary file 3 — Supplementary file3 (PDF 10116 KB) [file 10048_2022_705_MOESM3_ESM.pdf]

Supplementary Information S2: Manhattan plots of expression genome wide associations studies conducted on genes, which were differentially expressed between high and low feather pecking chickens.

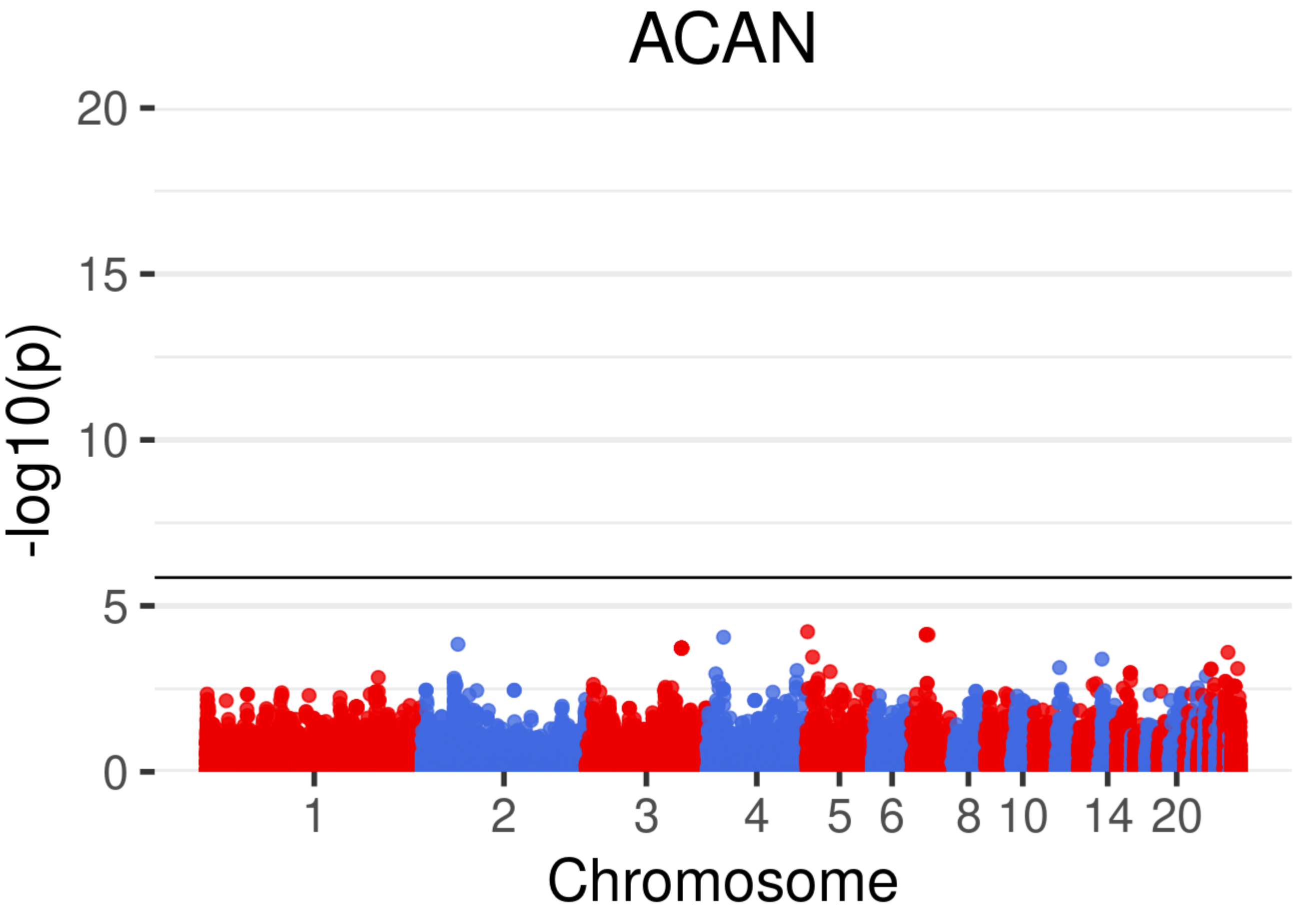

ACAN
20
15
10
5
0
-log10(p)
1
2
3
4
5
6
8
10
14
20
Chromosome

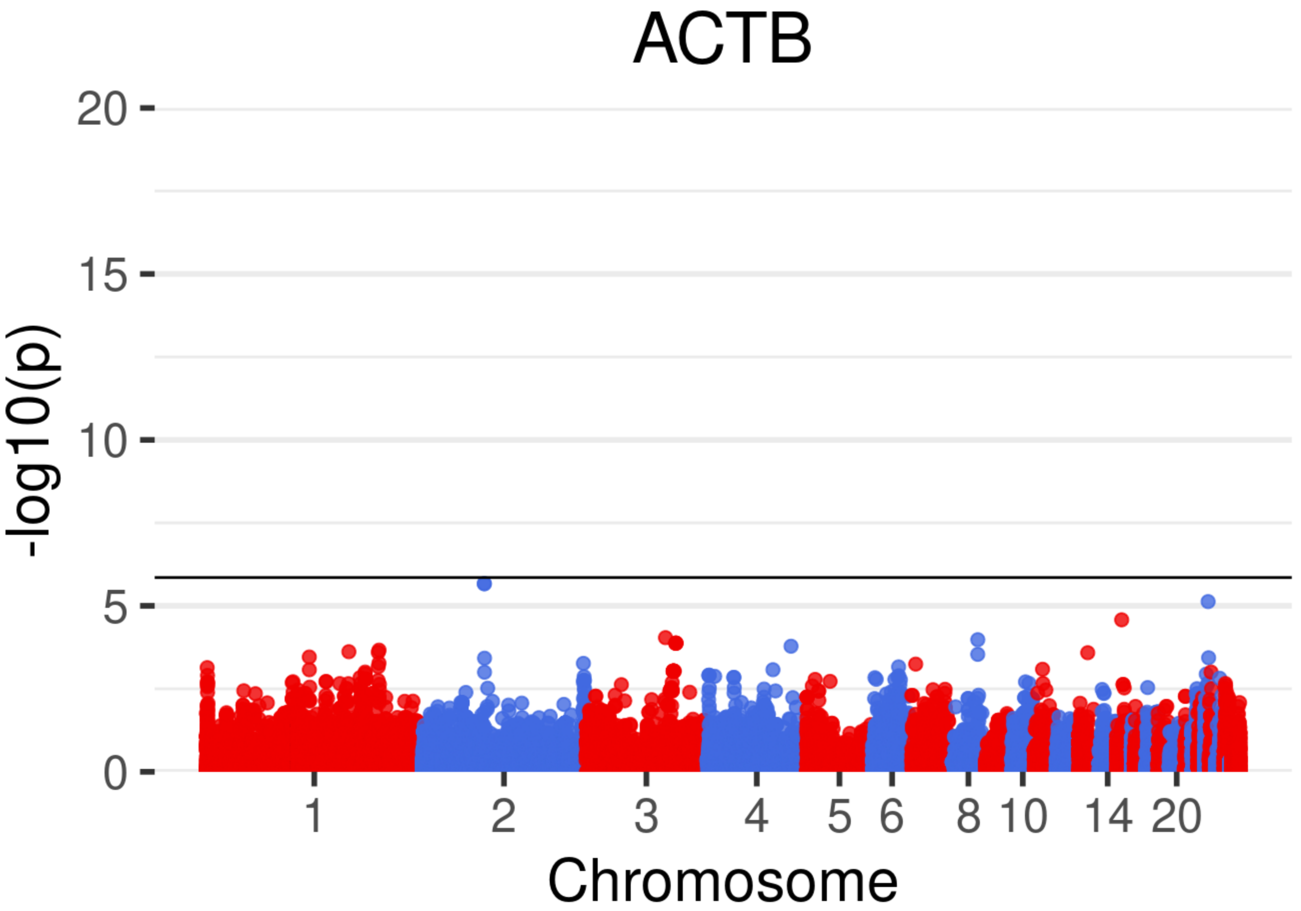
ACTB
-log10(p)
20
15
10
5
0
1
2
3
4
5
6
8
10
14
20
Chromosome

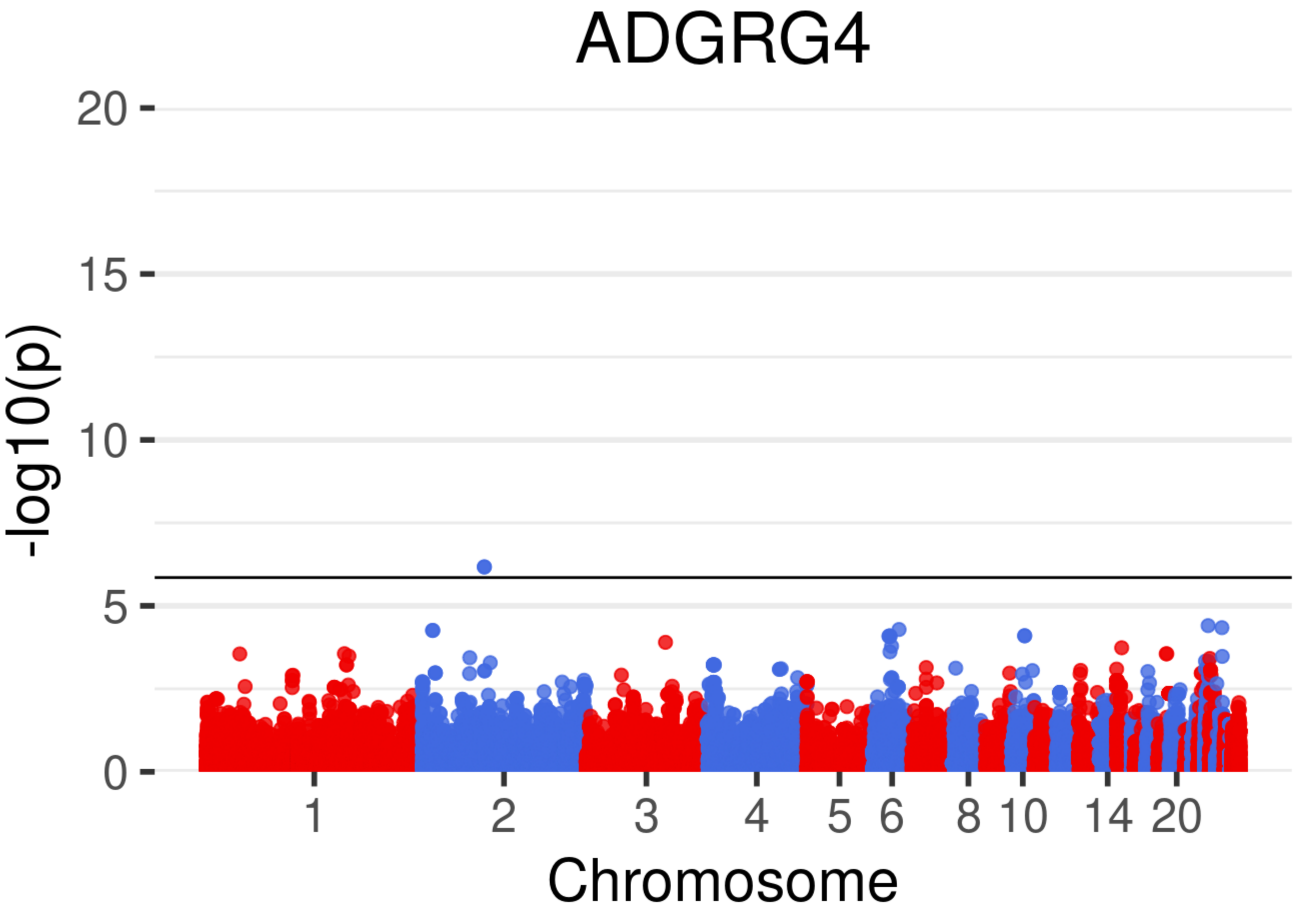
ADGRG4
20
15
10
5
0
-log10(p)
1
2
3
4
5
6
8
10
14
20
Chromosome

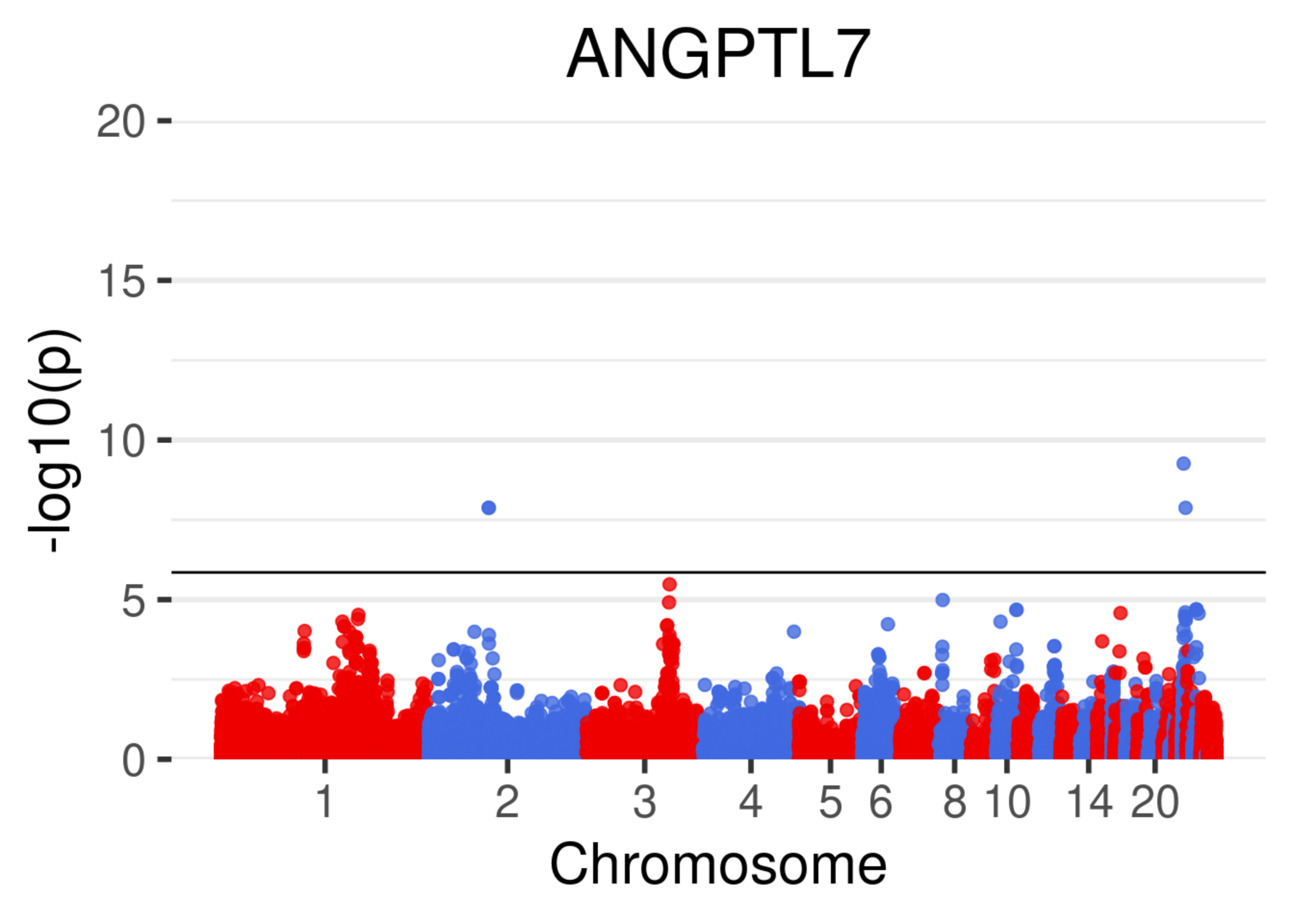
ANGPTL7
-log10(p)
20
15
10
5
0
1
2
3
4
5
6
8
10
14
20
Chromosome

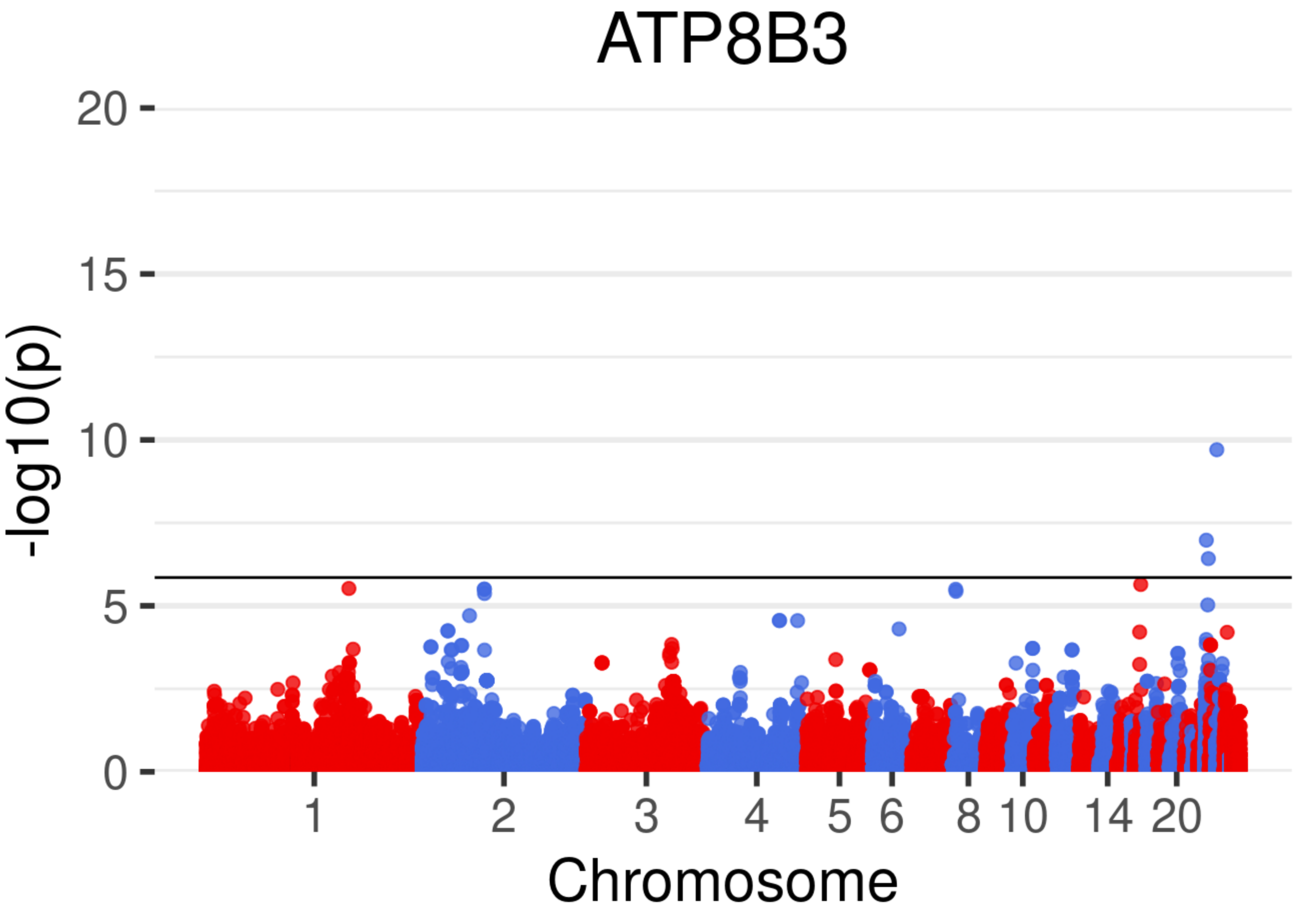

ATP8B3
-log10(p)
20
15
10
5
0
1
2
3
4
5
6
8
10
14
20
Chromosome

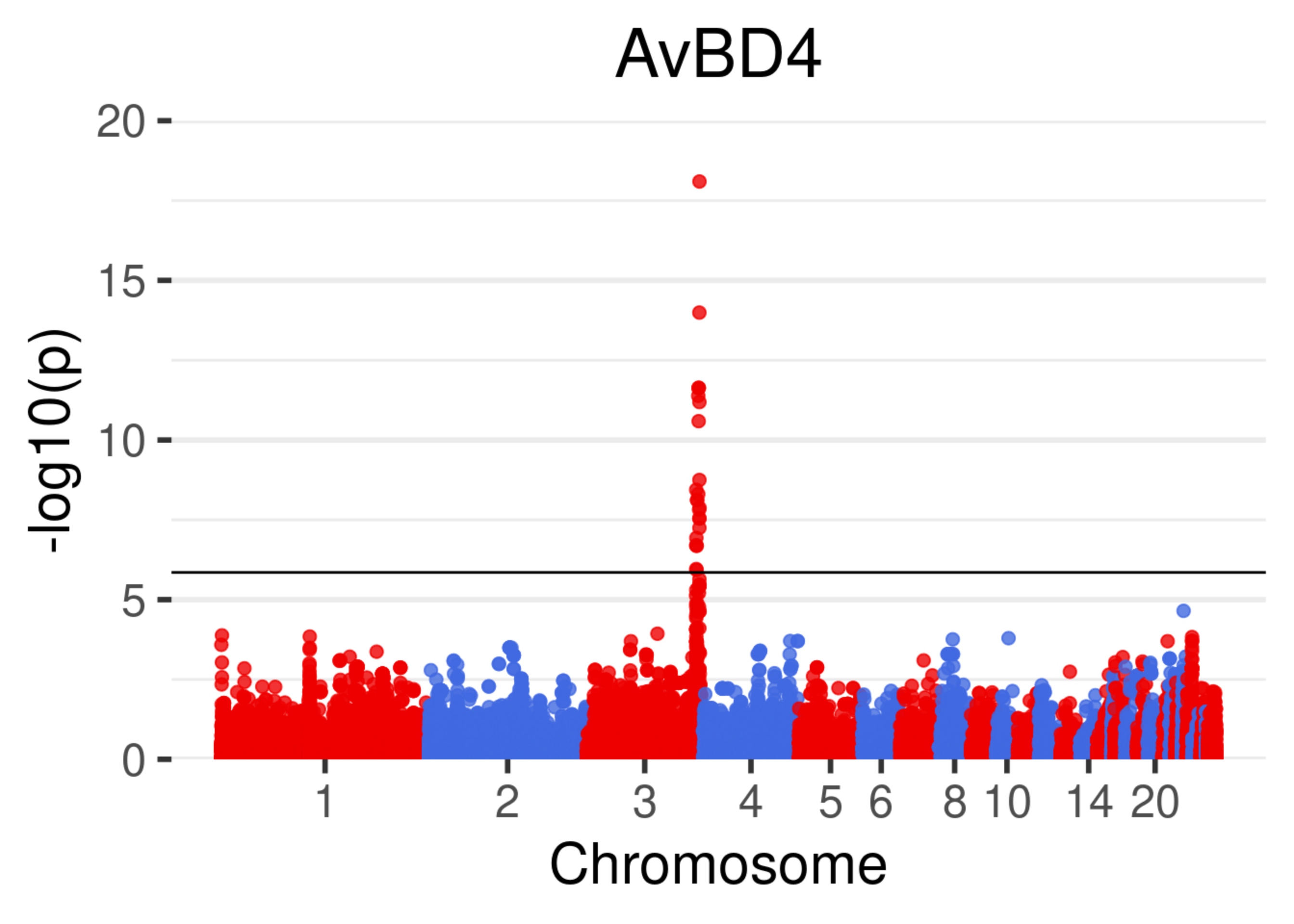

AvBD4
-log10(p)
20
15
10
5
0
1
2
3
4
5
6
8
10
14
20
Chromosome

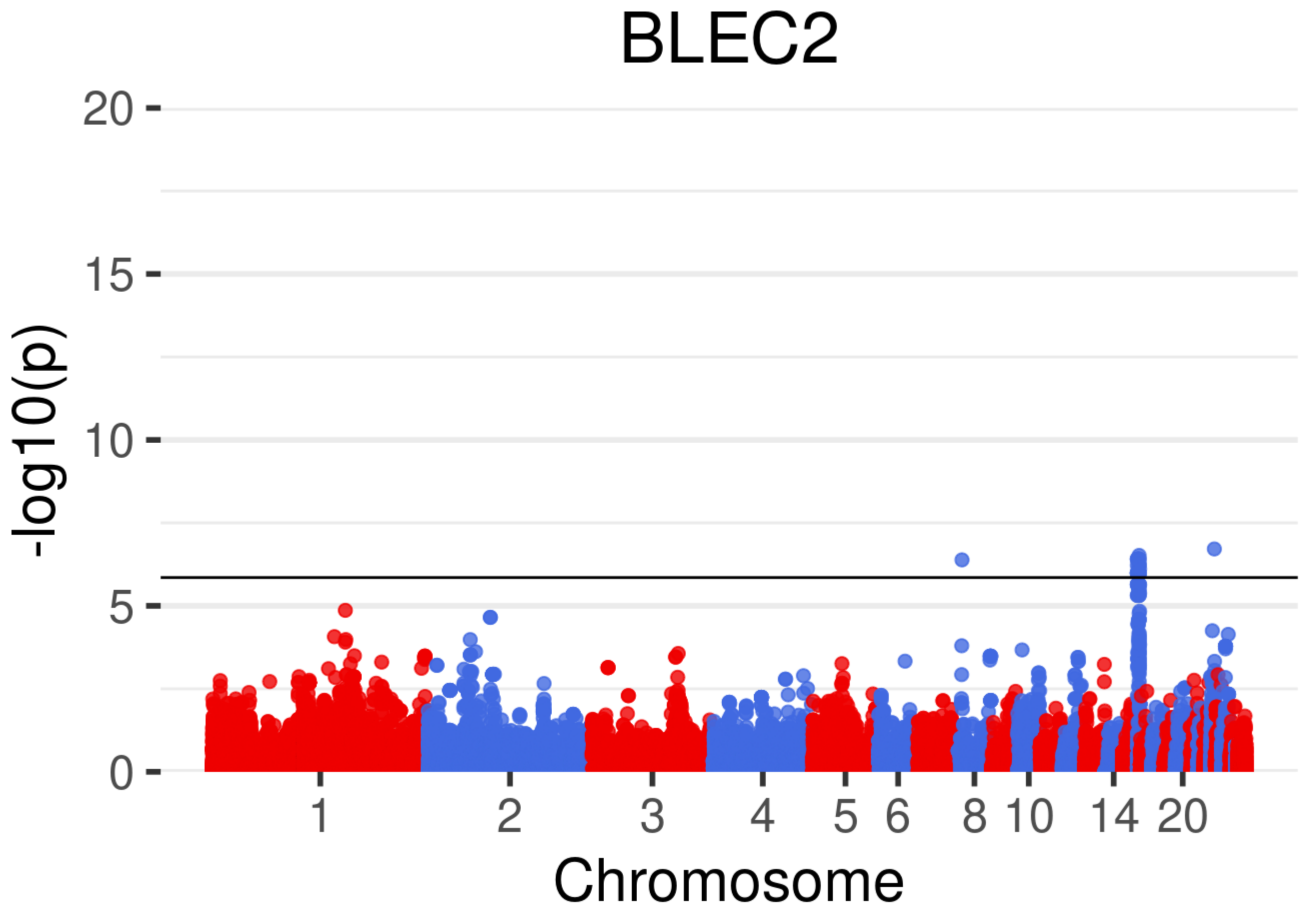
BLEC2
-log10(p)
20
15
10
5
0
1
2
3
4
5
6
8
10
14
20
Chromosome

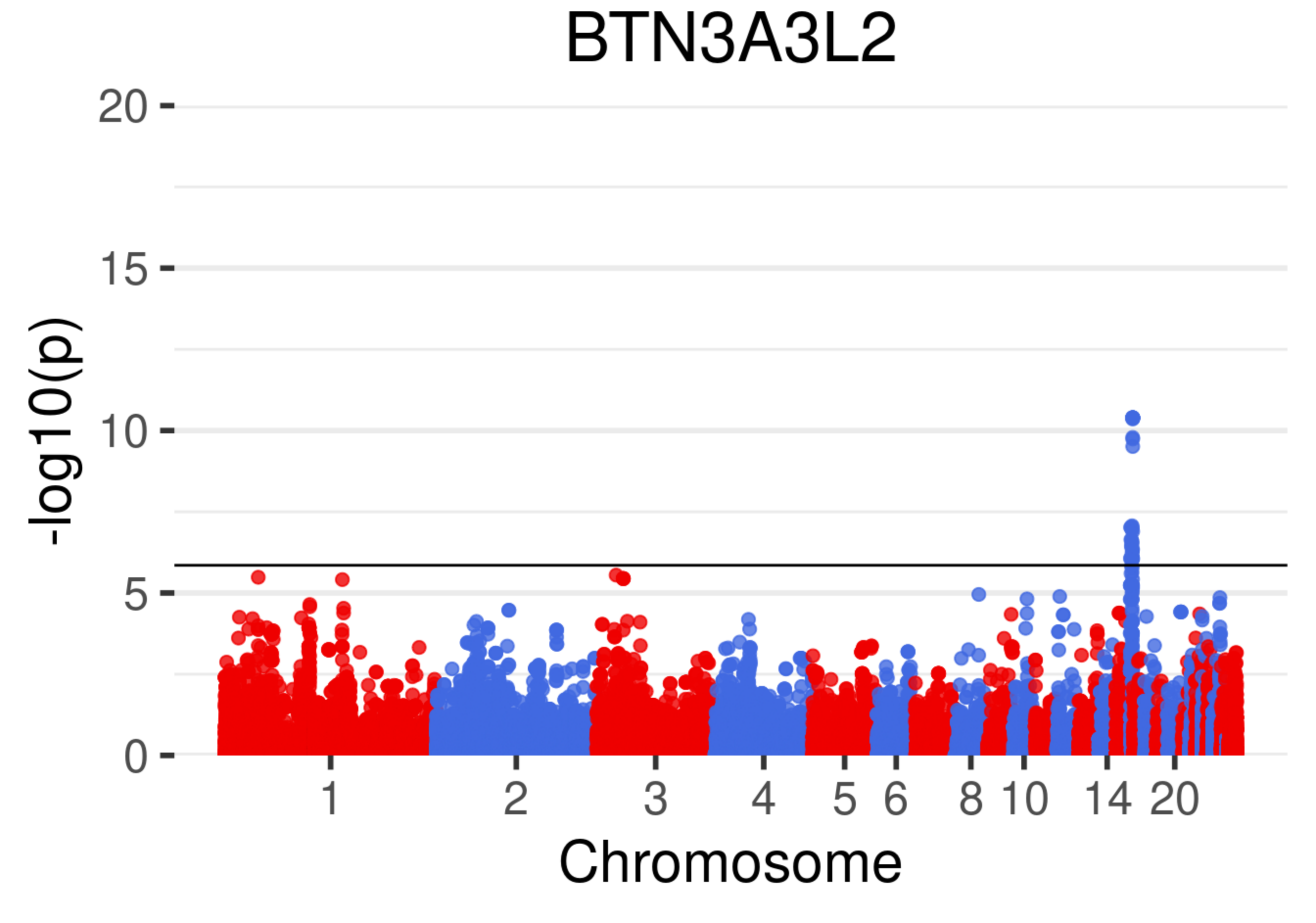

BTN3A3L2
20
15
10
5
0
-log10(p)
1
2
3
4
5
6
8
10
14
20
Chromosome

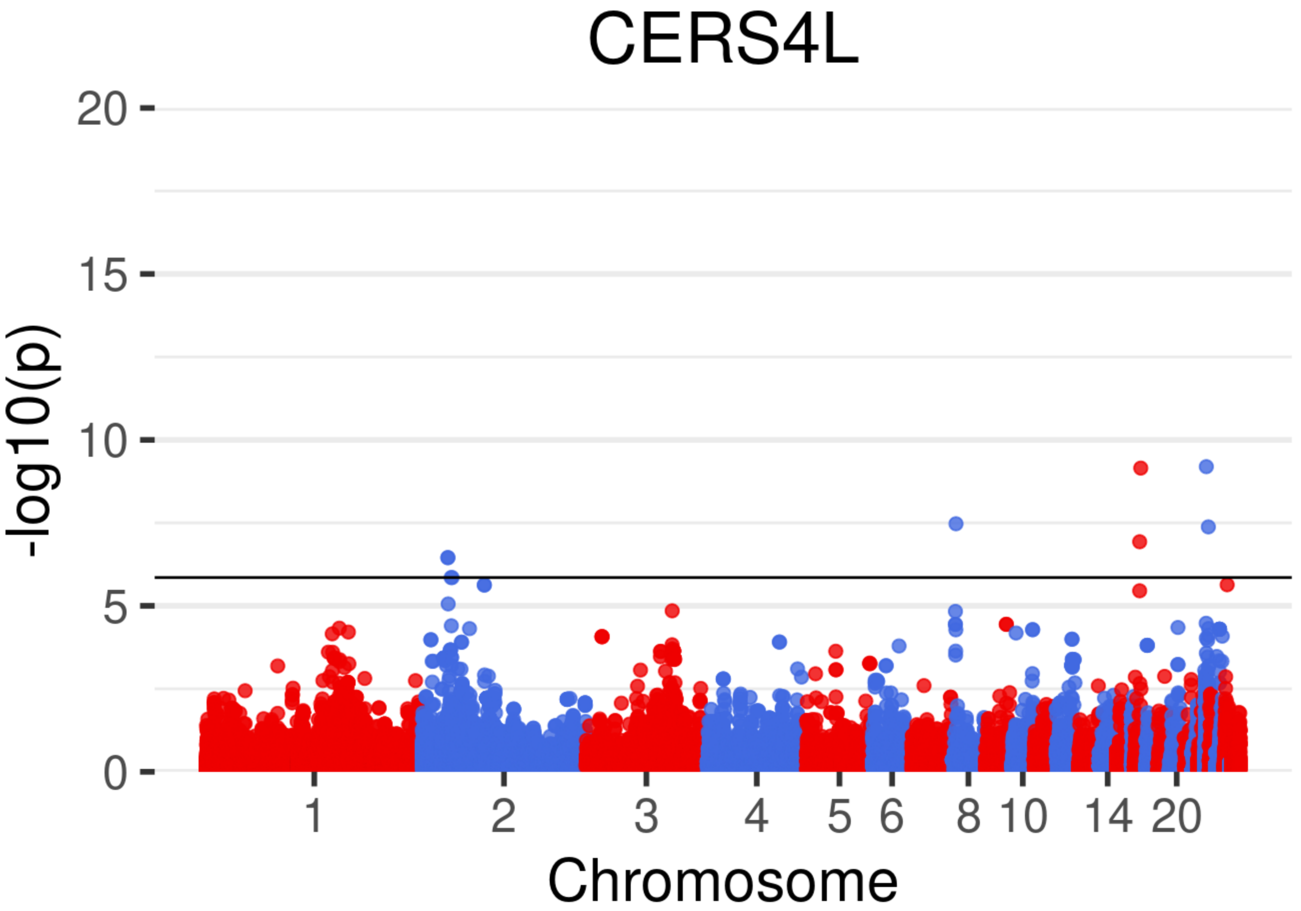
CERS4L
-log10(p)
20
15
10
5
0
1
2
3
4
5
6
8
10
14
20
Chromosome

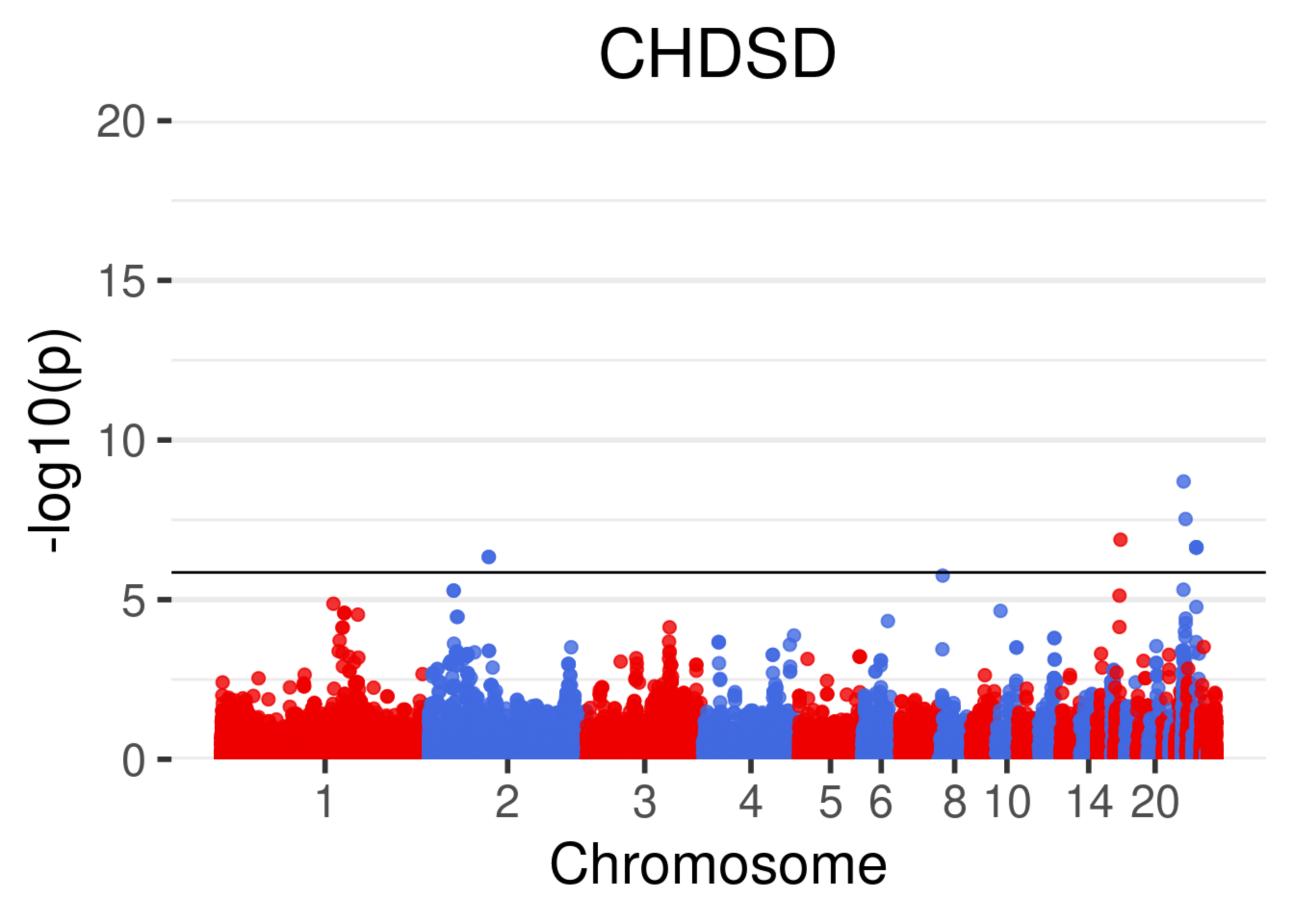
CHDSD
20
15
10
5
0
-log10(p)
1
2
3
4
5
6
8
10
14
20
Chromosome

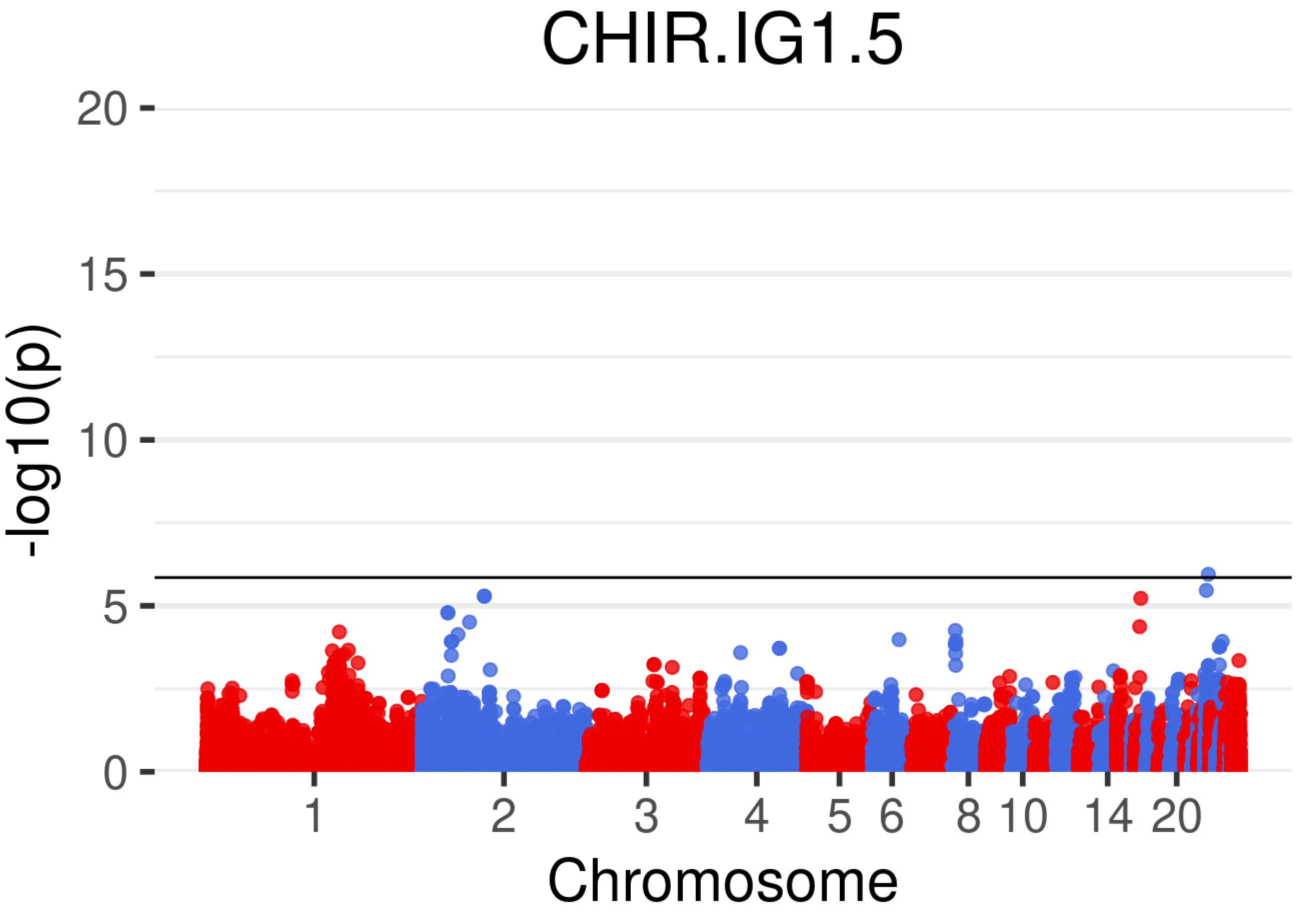

CHIR.IG1.5
-log10(p)
20
15
10
5
0
1
2
3
4
5
6
8
10
14
20
Chromosome

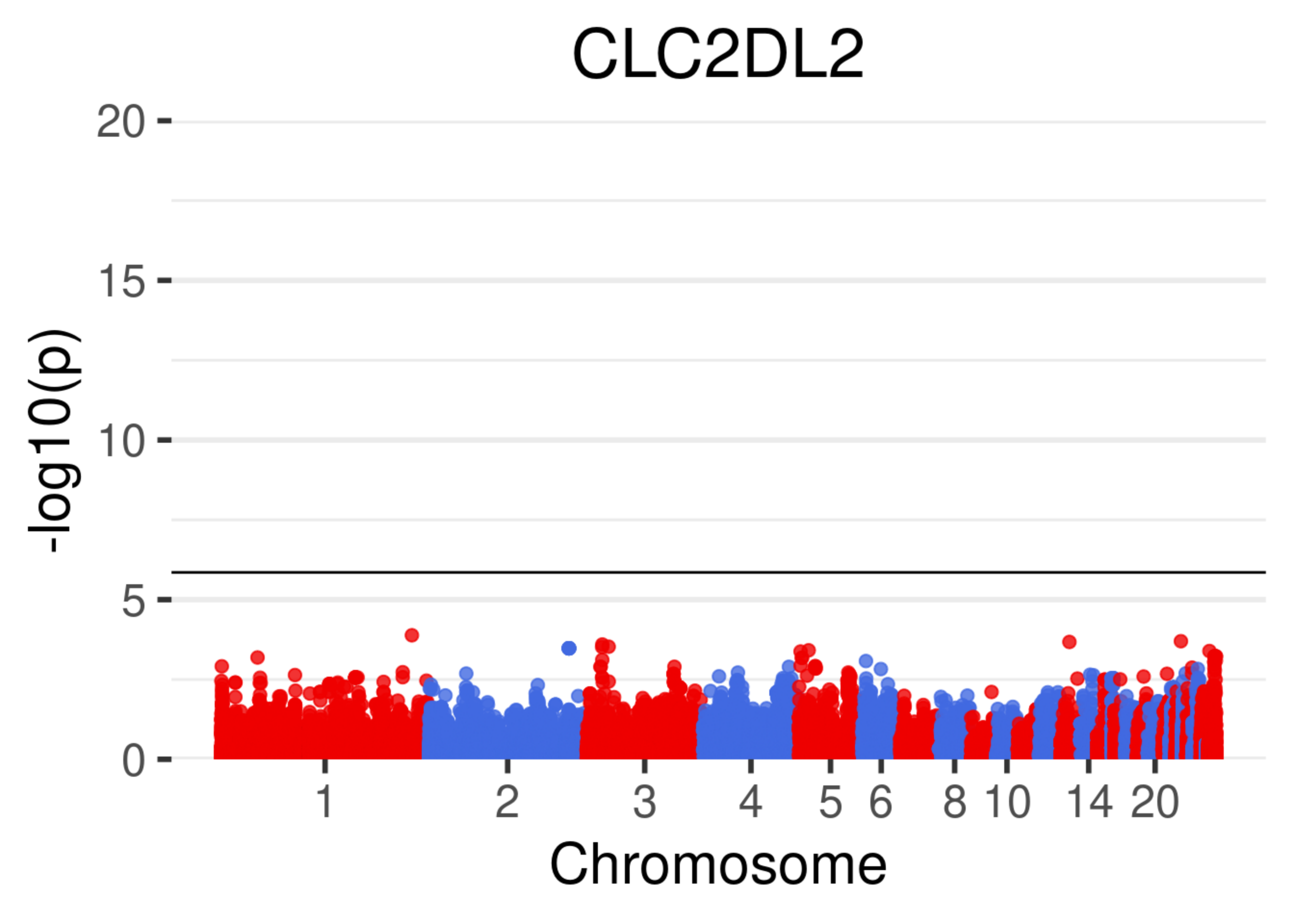
CLC2DL2
-log10(p)
20
15
10
5
0
1
2
3
4
5
6
8
10
14
20
Chromosome

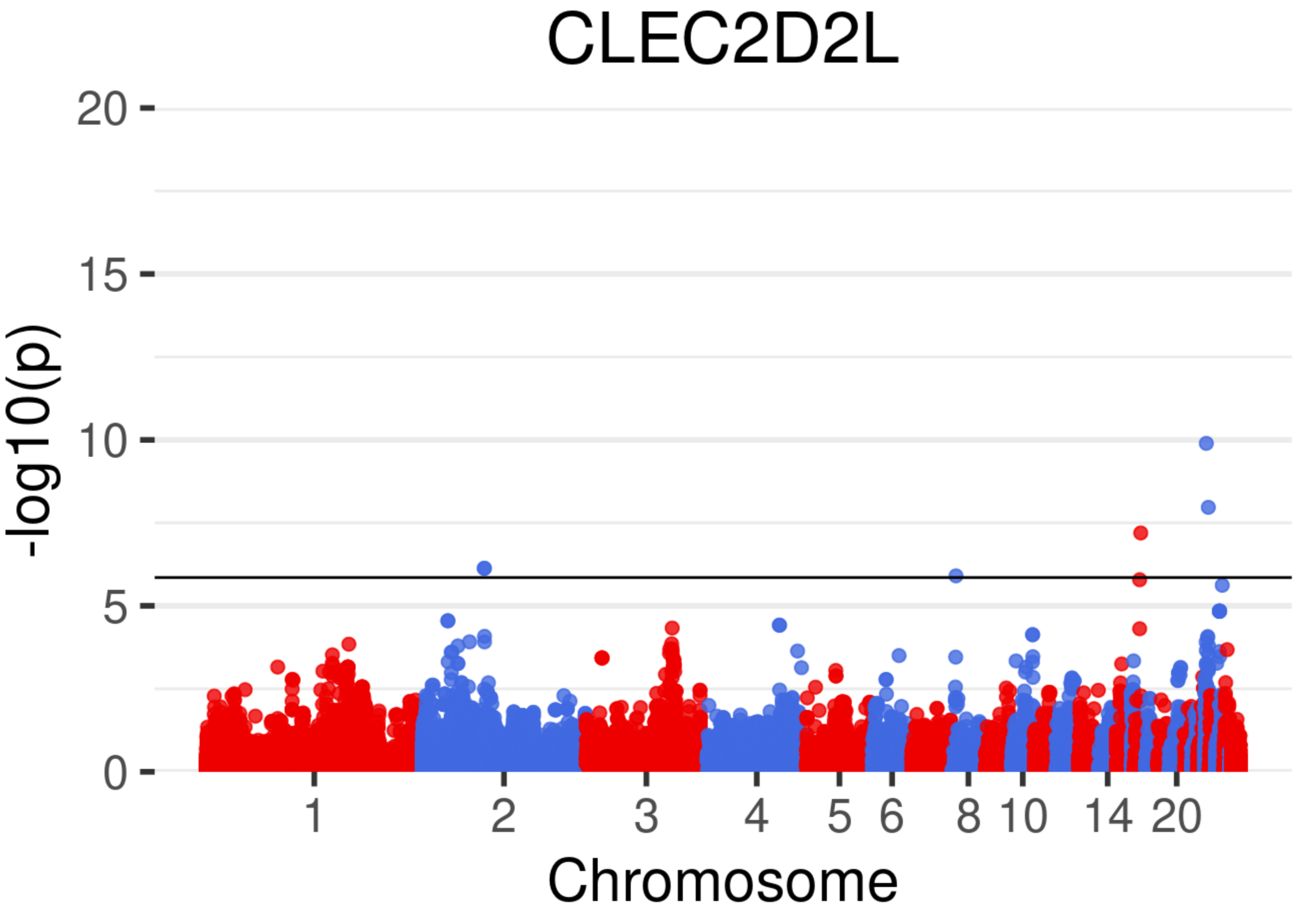

CLEC2D2L
-log10(p)
20
15
10
5
0
1
2
3
4
5
6
8
10
14
20
Chromosome

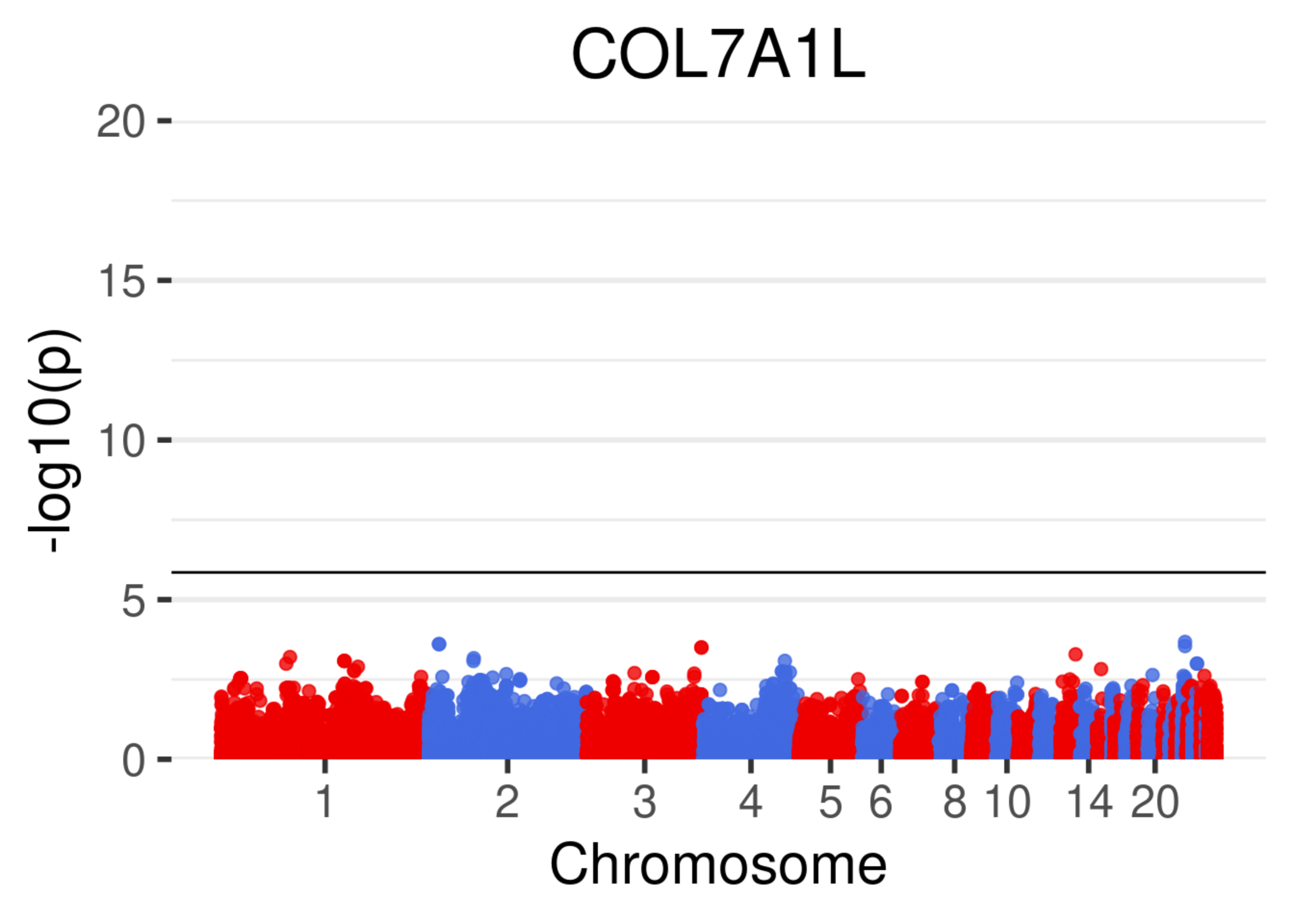
COL7A1L
-log10(p)
20
15
10
5
0
1
2
3
4
5
6
8
10
14
20
Chromosome

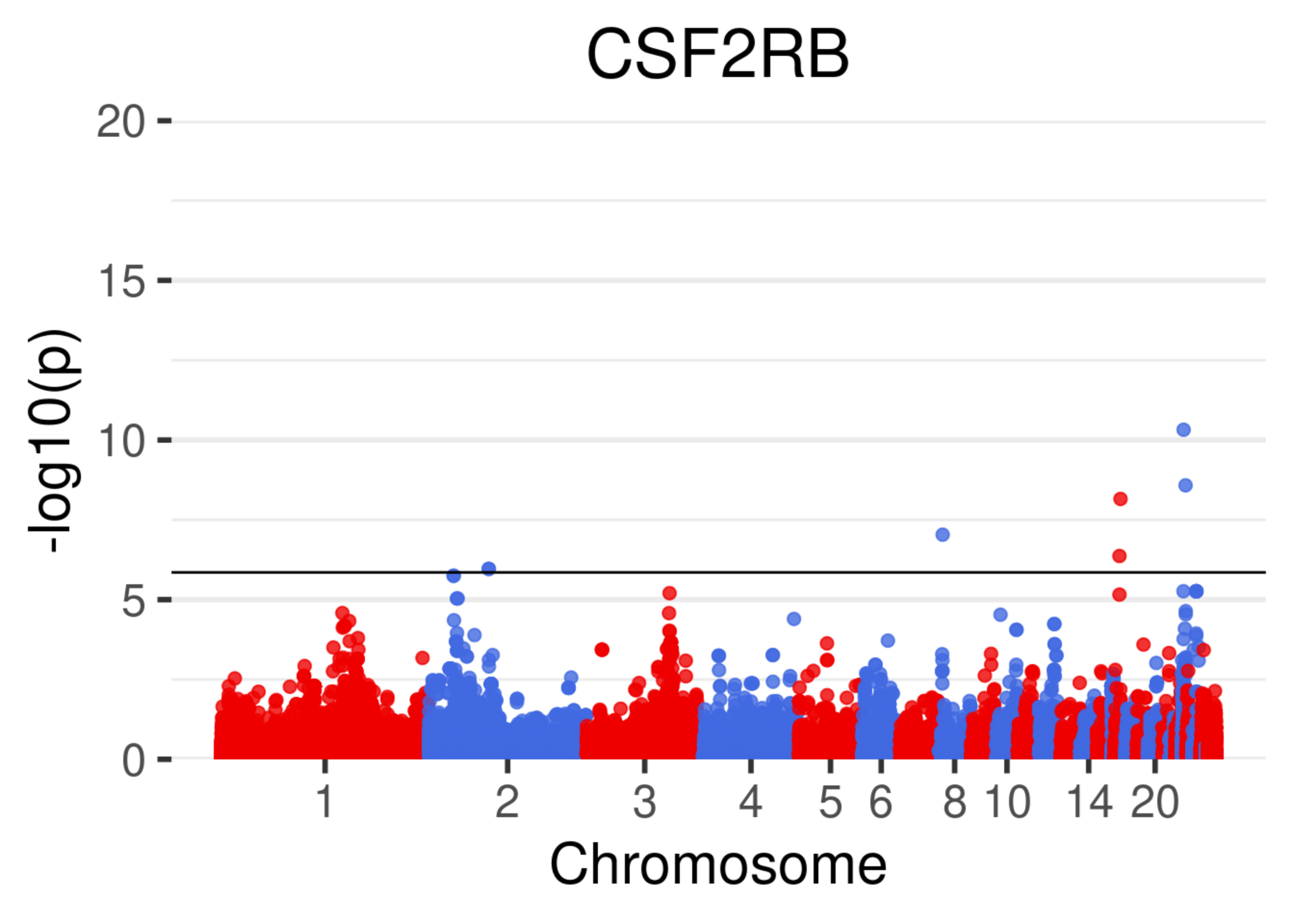
CSF2RB
-log10(p)
0
5
10
15
20
1
2
3
4
5
6
8
10
14
20
Chromosome

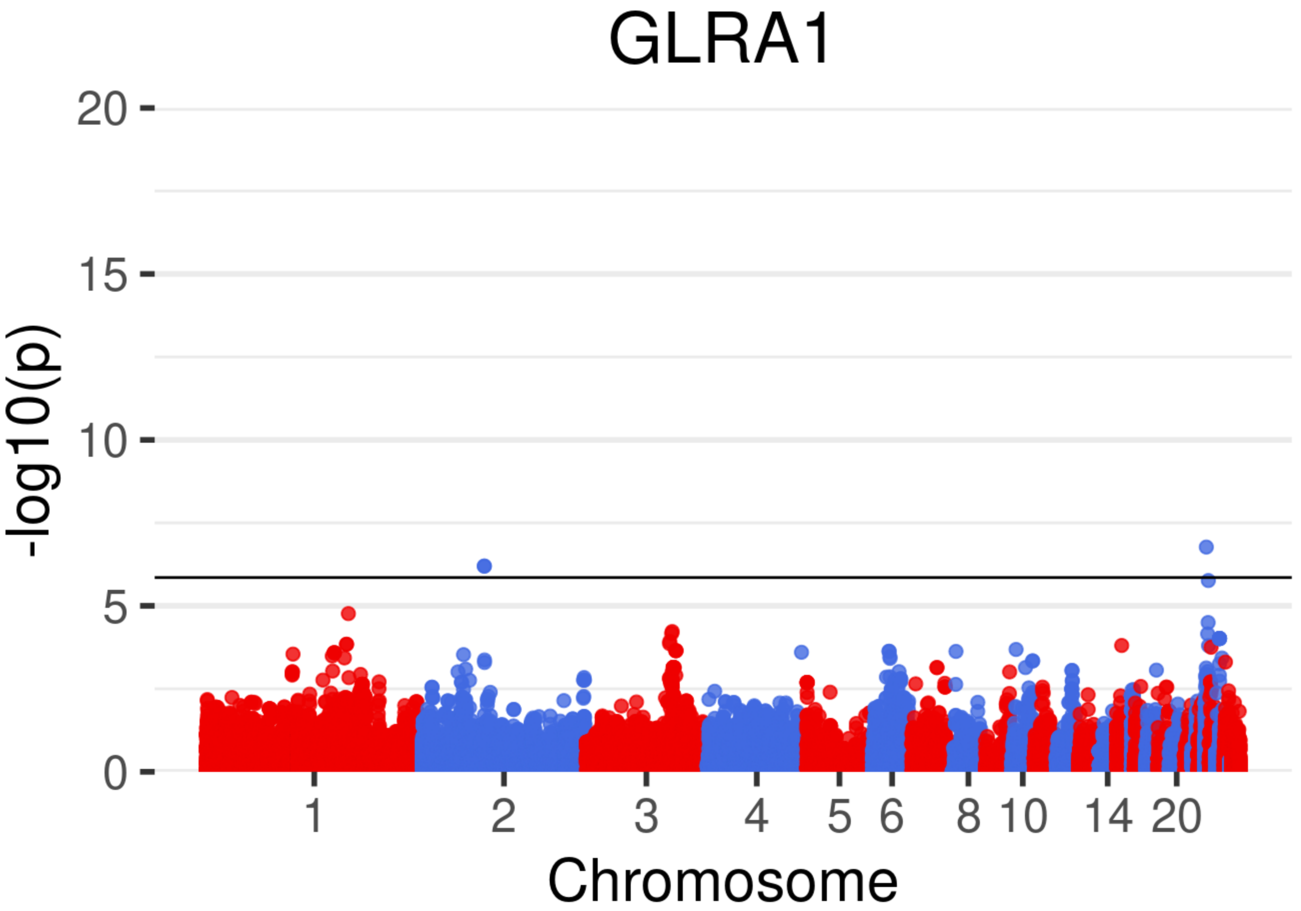

GLRA1
20
15
10
5
0
-log10(p)
1
2
3
4
5
6
8
10
14
20
Chromosome

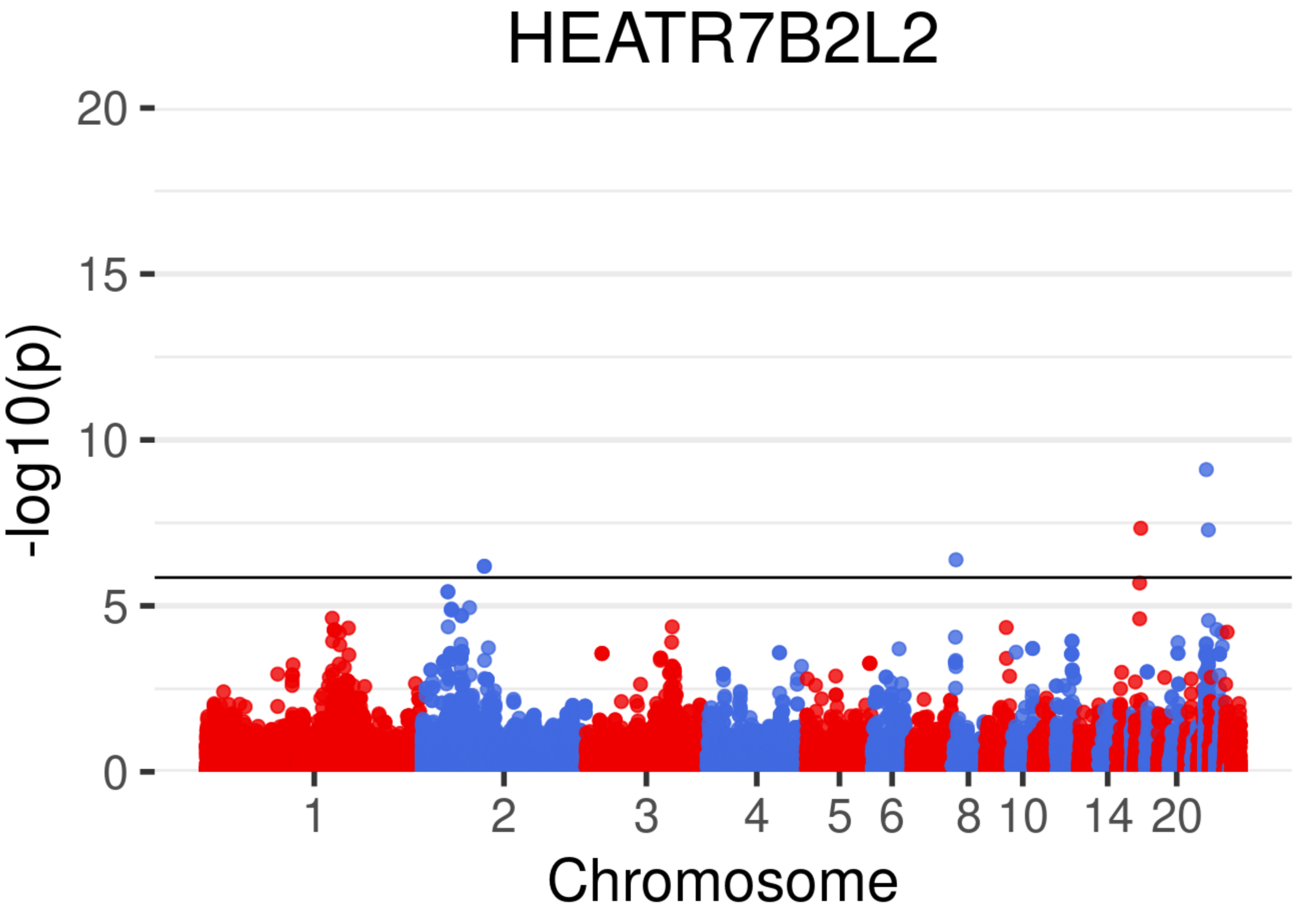
HEATR7B2L2
-log10(p)
20
15
10
5
0
1
2
3
4
5
6
8
10
14
20
Chromosome

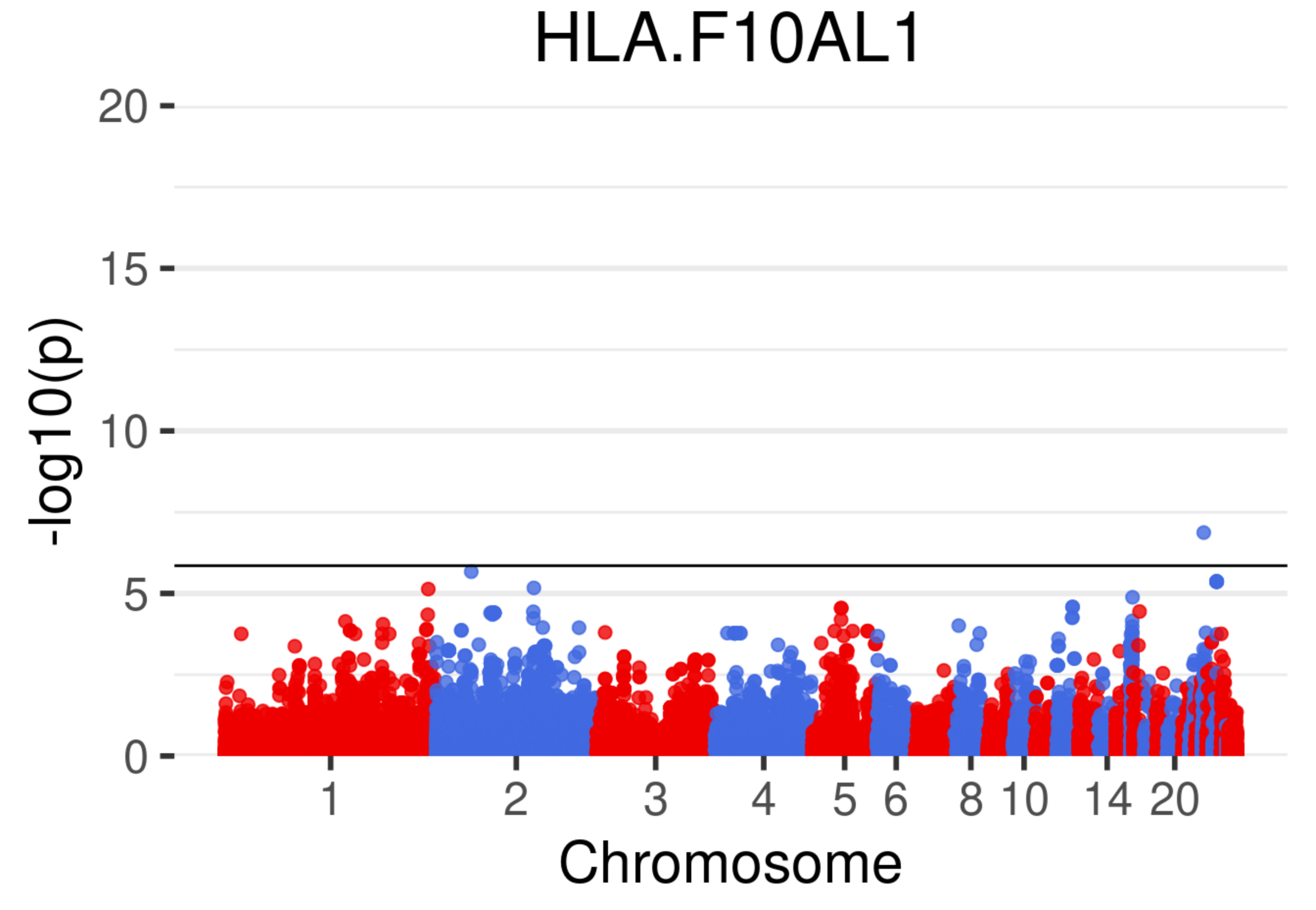
HLA.F10AL1
20
15
10
5
0
-log10(p)
1
2
3
4
5
6
8
10
14
20
Chromosome

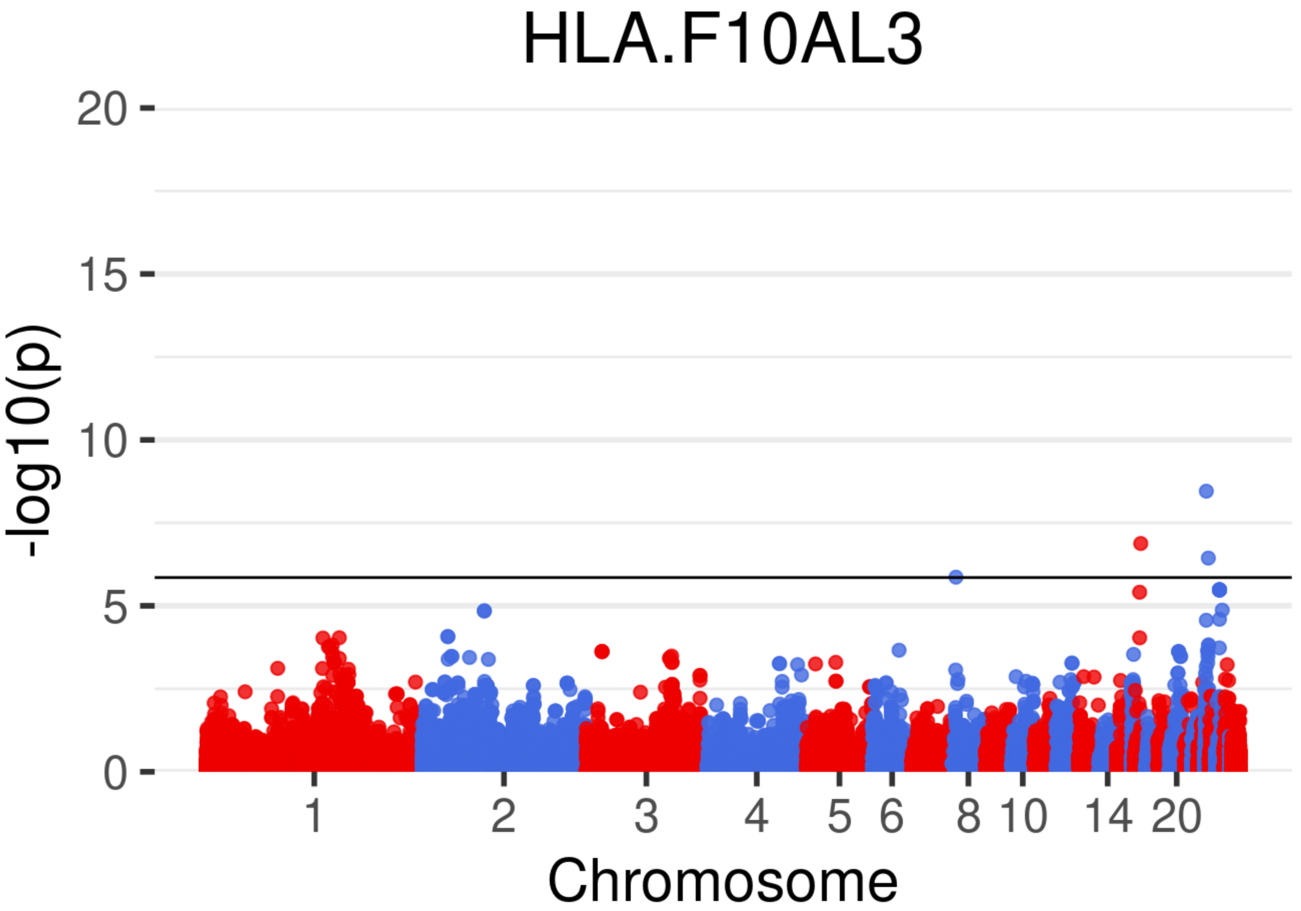
HLA.F10AL3
-log10(p)
20
15
10
5
0
1
2
3
4
5
6
8
10
14
20
Chromosome

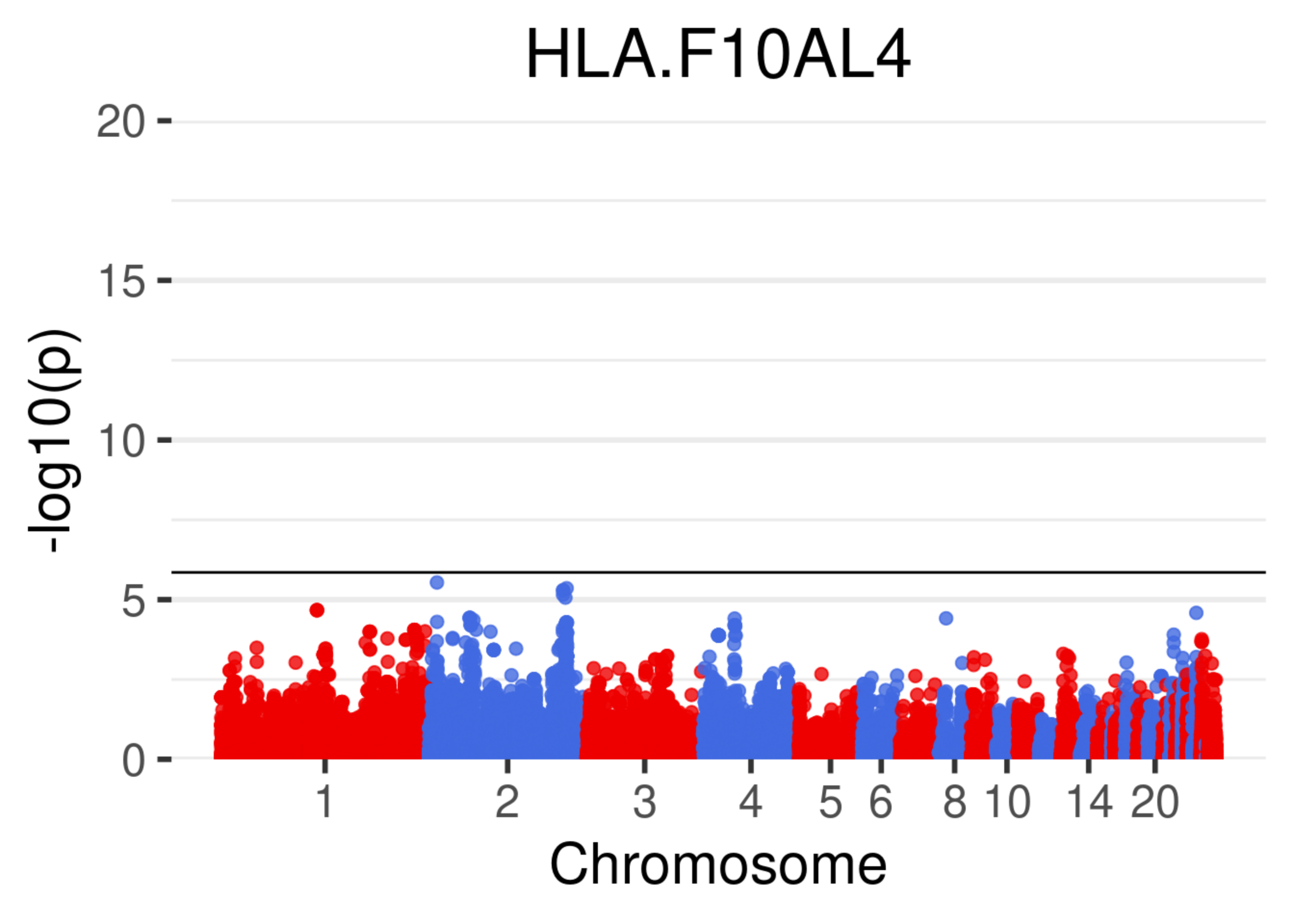

HLA.F10AL4
-log10(p)
20
15
10
5
0
1
2
3
4
5
6
8
10
14
20
Chromosome

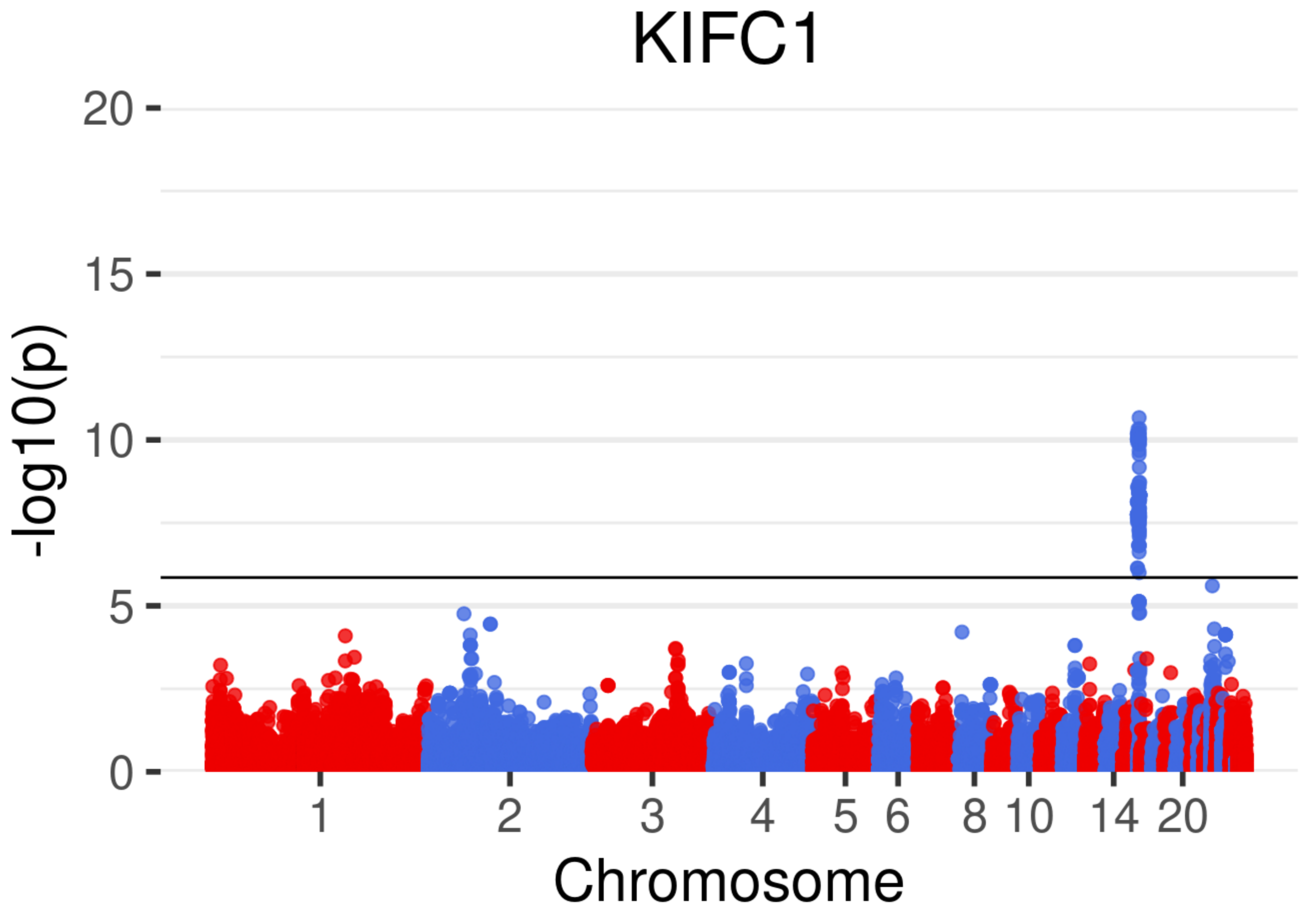
KIFC1
-log10(p)
0
5
10
15
20
Chromosome
1
2
3
4
5
6
8
10
14
20

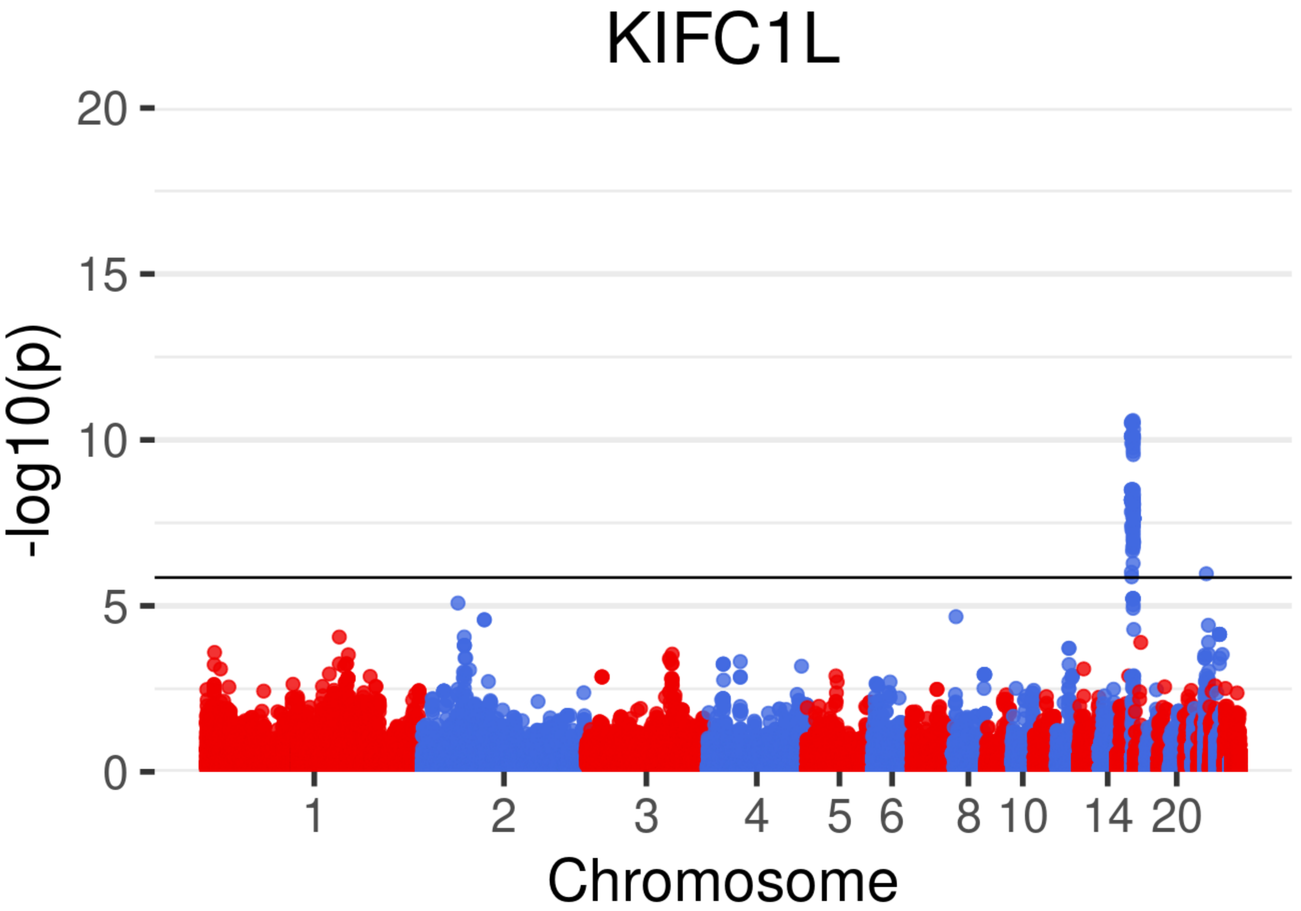
KIFC1L
-log10(p)
20
15
10
5
0
1
2
3
4
5
6
8
10
14
20
Chromosome

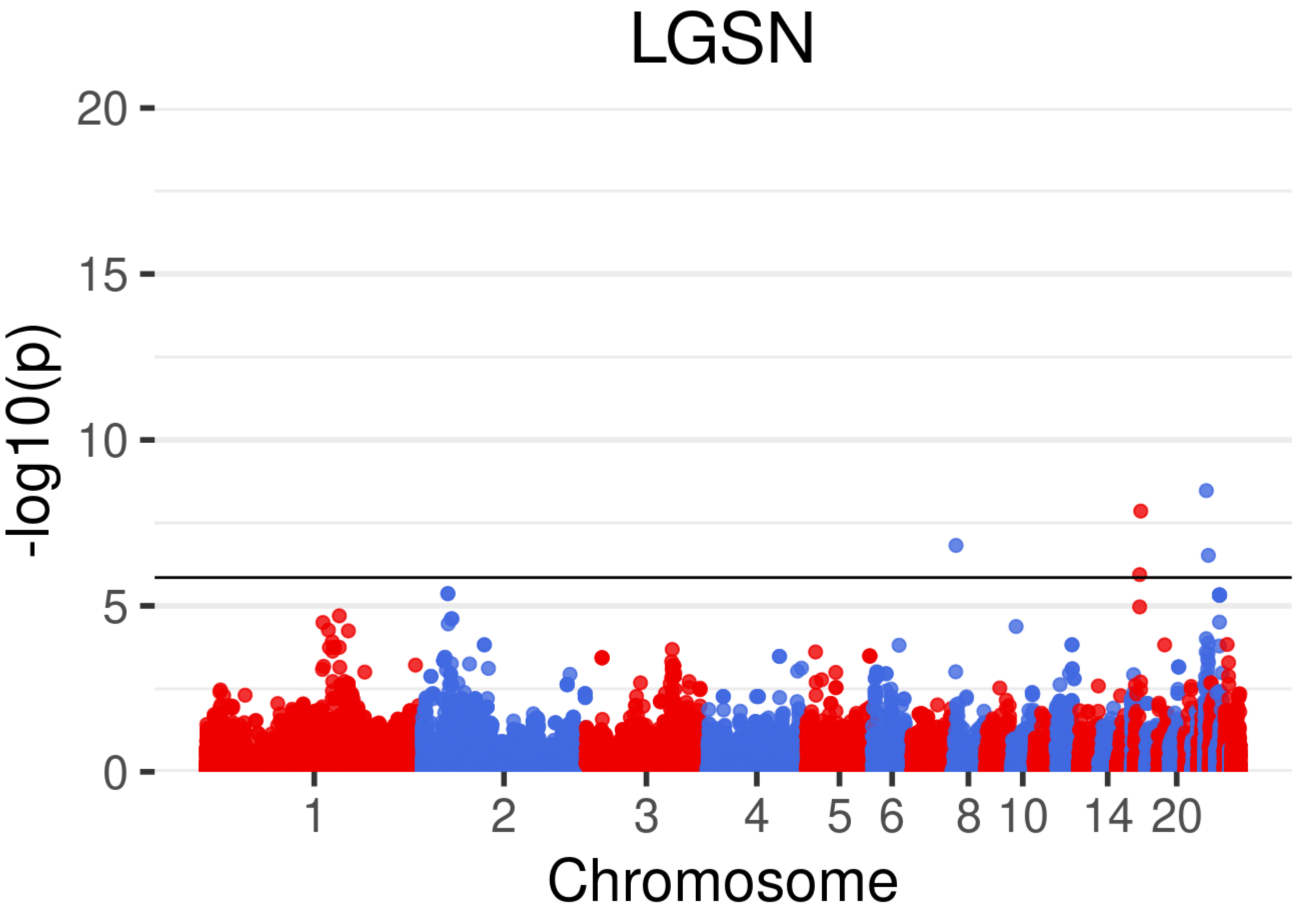

LGSN
-log10(p)
20
15
10
5
0
1
2
3
4
5
6
8
10
14
20
Chromosome

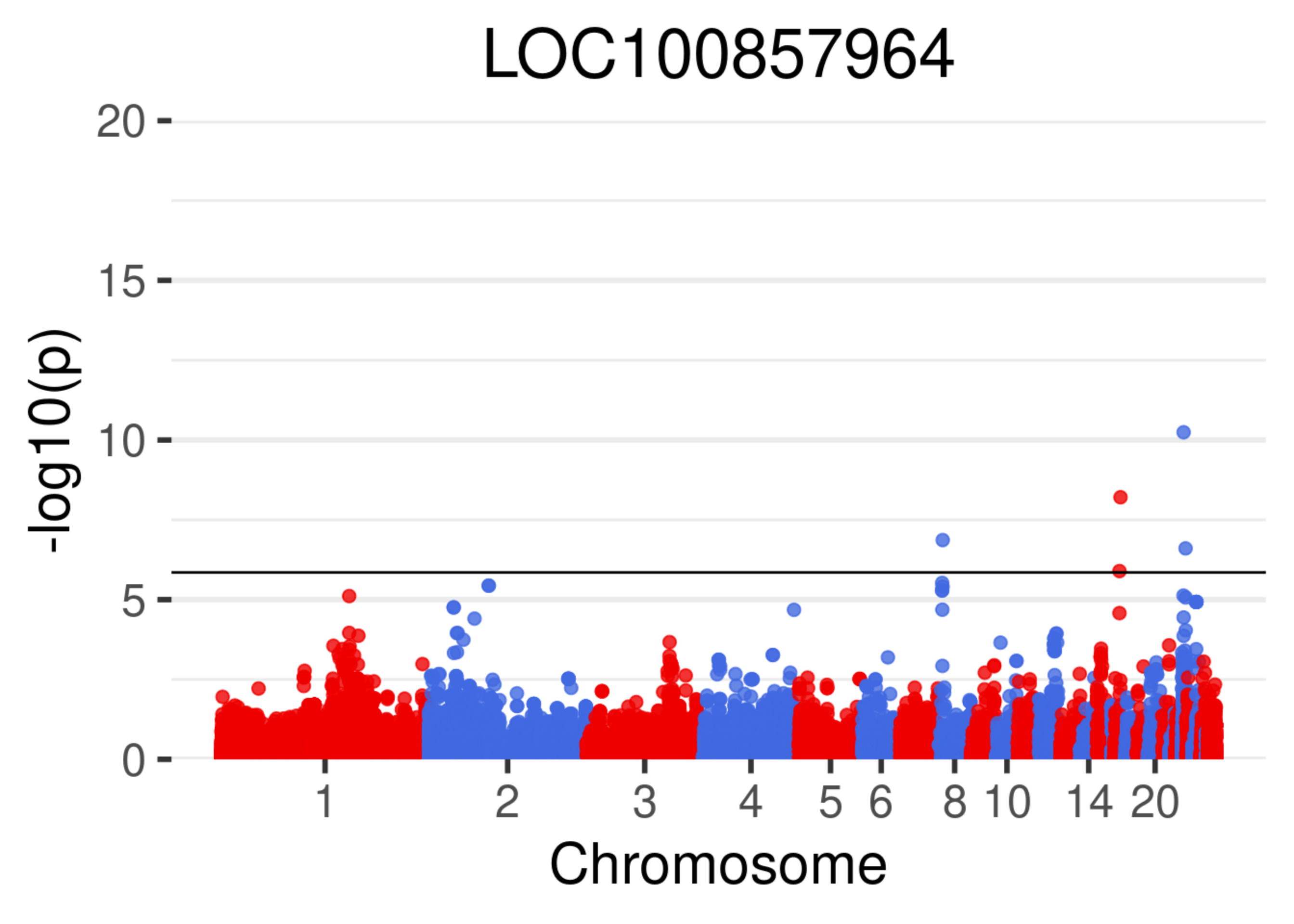

LOC100857964
-log10(p)
20
15
10
5
0
1
2
3
4
5
6
8
10
14
20
Chromosome

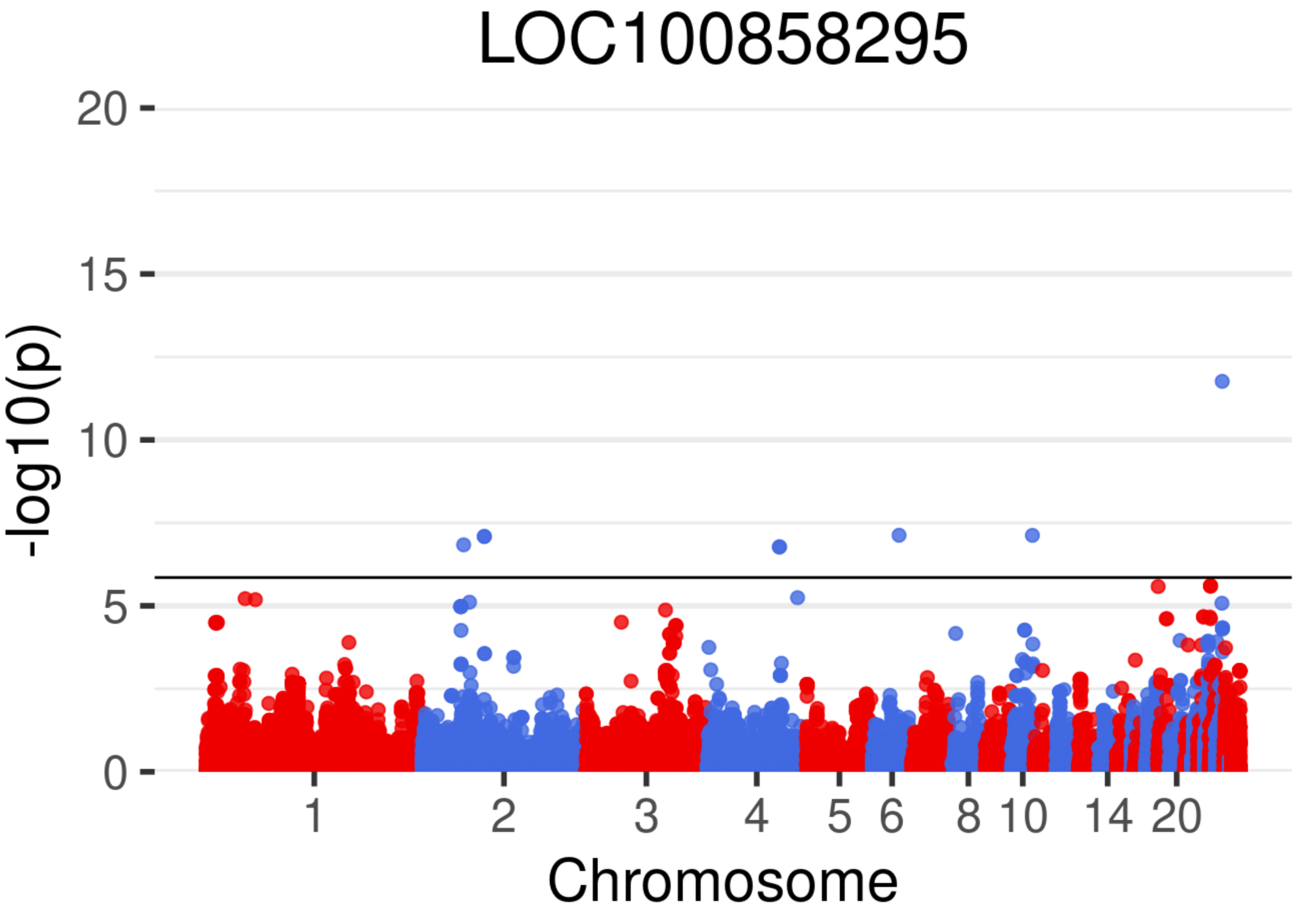
LOC100858295
-log10(p)
20
15
10
5
0
1
2
3
4
5
6
8
10
14
20
Chromosome

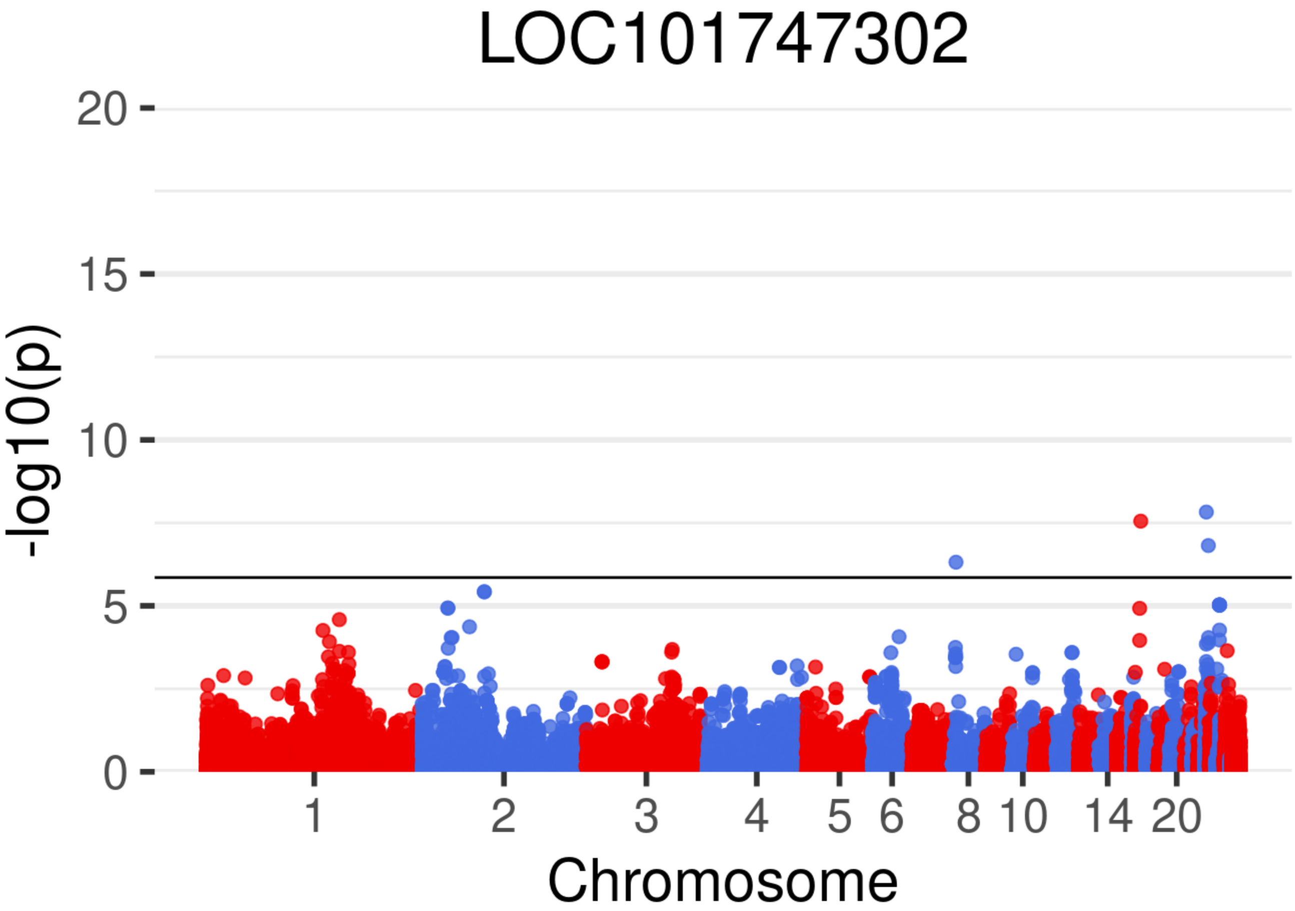
LOC101747302
-log10(p)
20
15
10
5
0
1
2
3
4
5
6
8
10
14
20
Chromosome

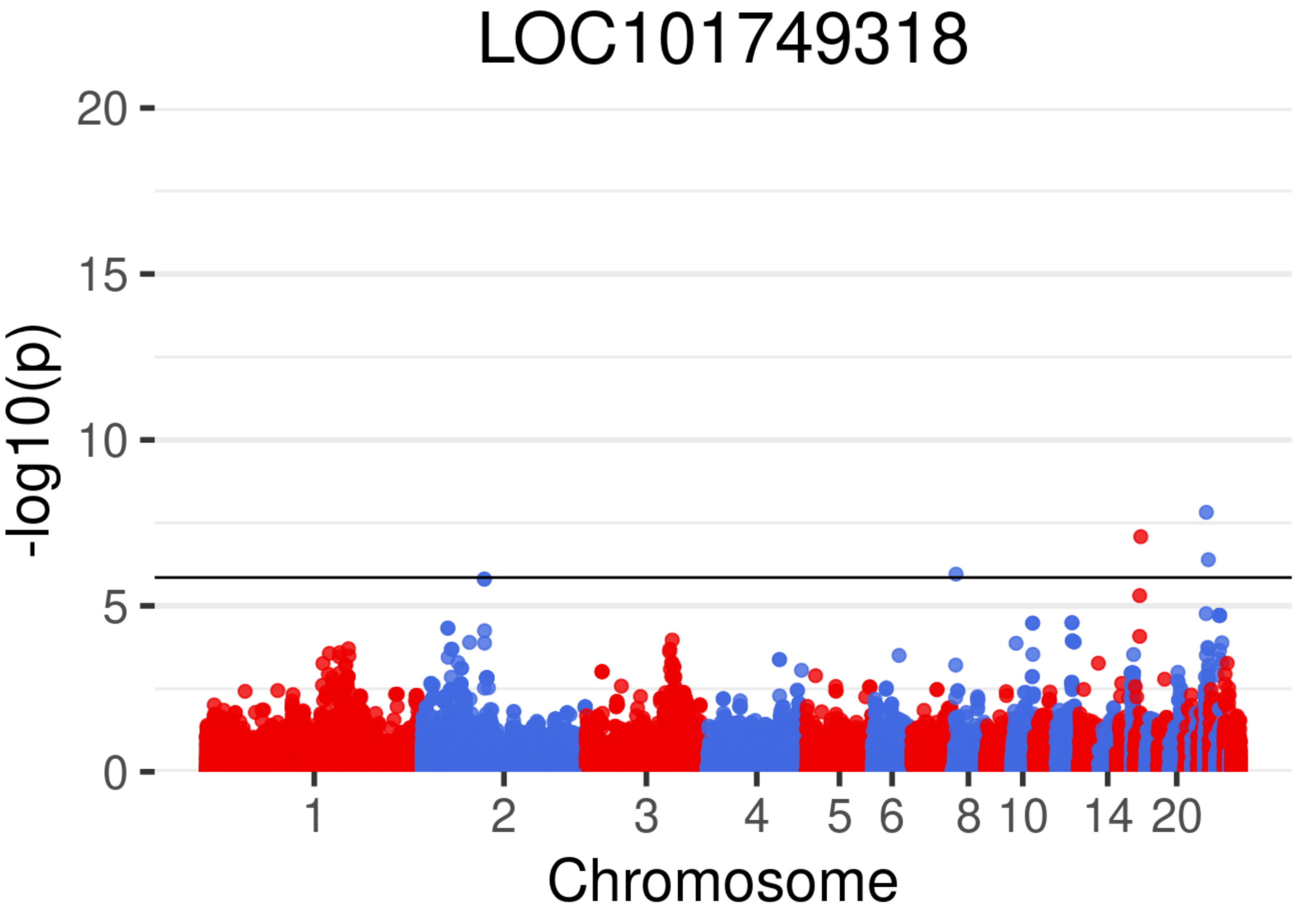

LOC101749318
-log10(p)
20
15
10
5
0
1
2
3
4
5
6
8
10
14
20
Chromosome

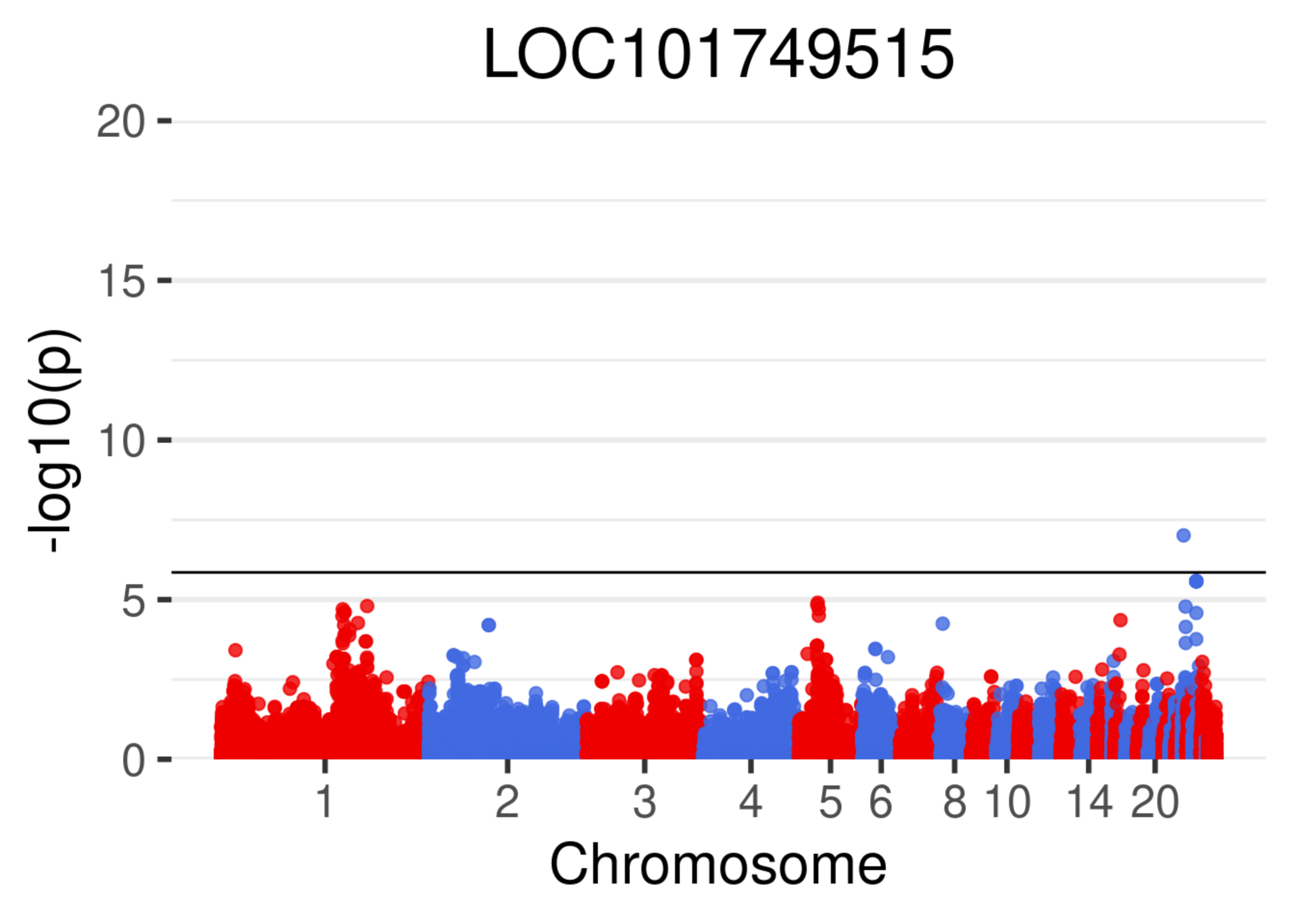
LOC101749515
-log10(p)
20
15
10
5
0
1
2
3
4
5
6
8
10
14
20
Chromosome

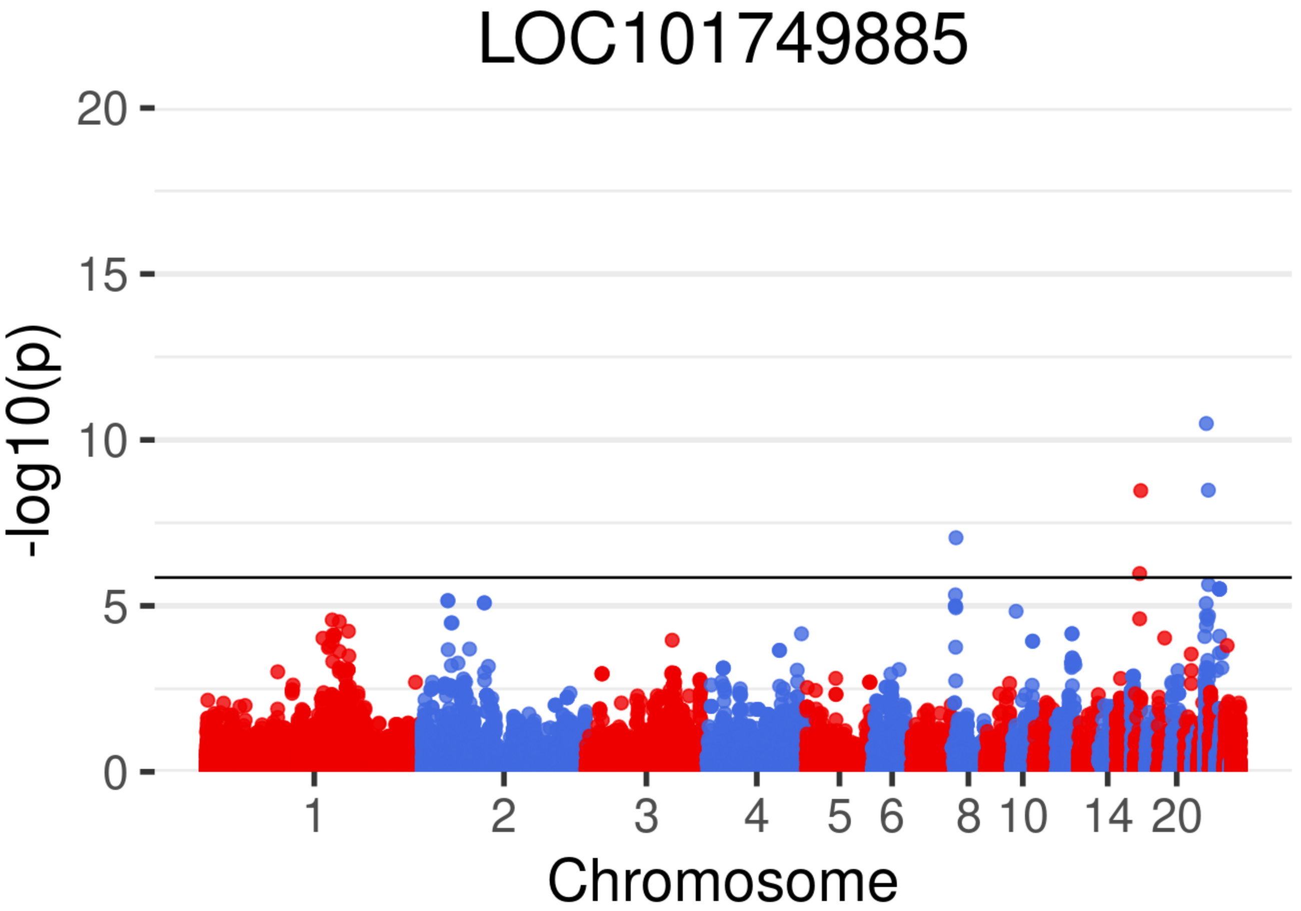
LOC101749885
-log10(p)
20
15
10
5
0
1
2
3
4
5
6
8
10
14
20
Chromosome

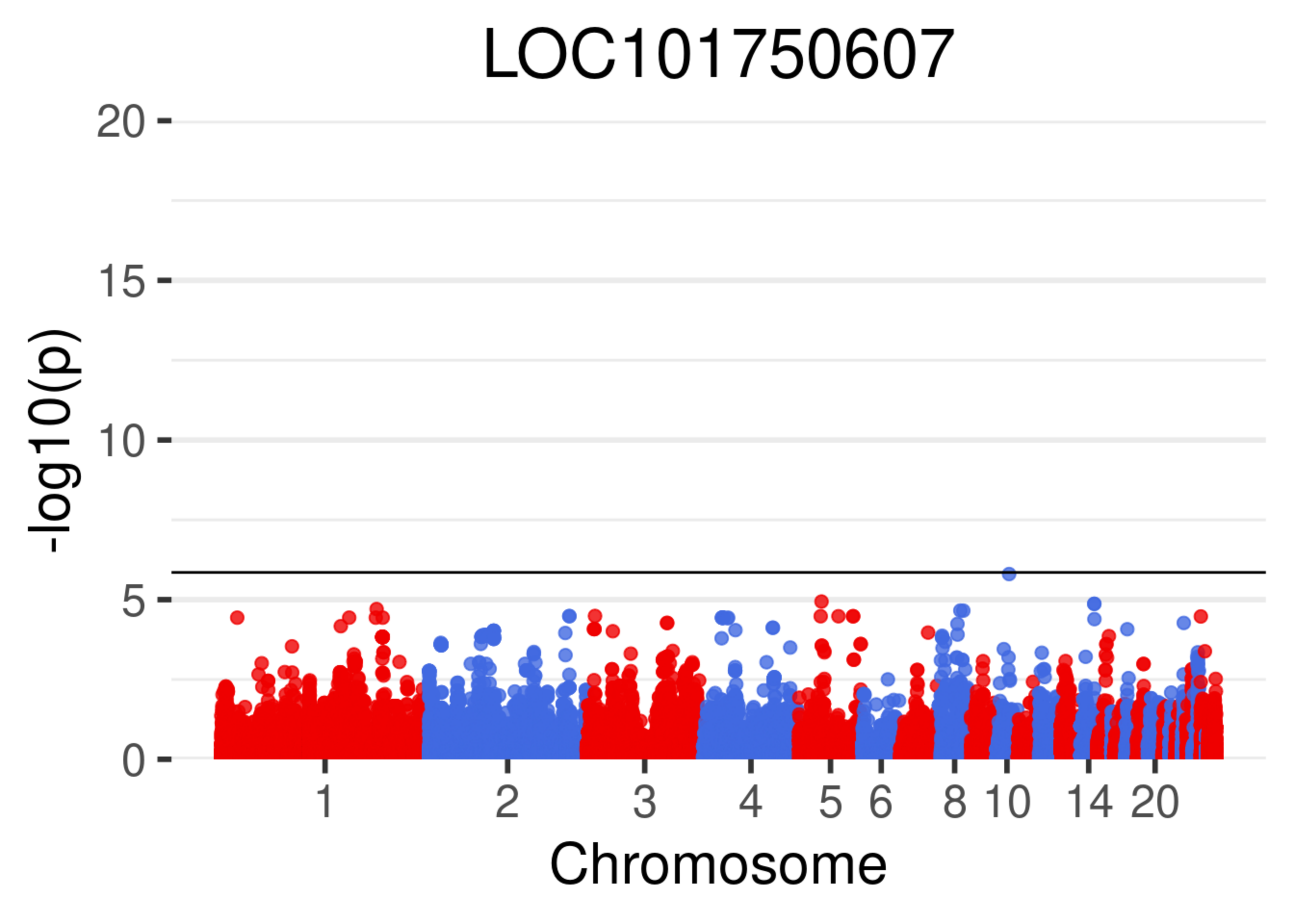

LOC101750607
-log10(p)
20
15
10
5
0
1
2
3
4
5
6
8
10
14
20
Chromosome

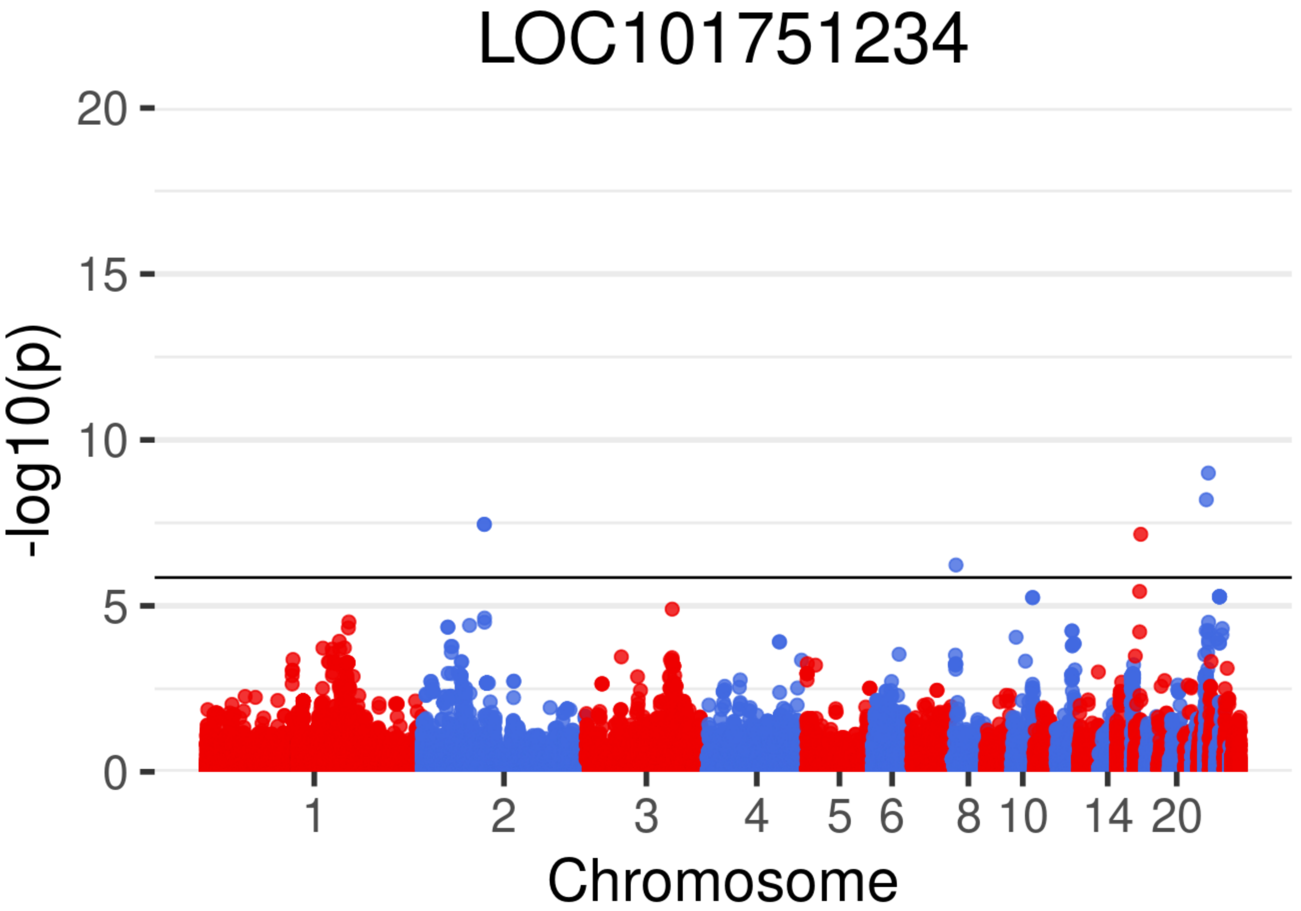
LOC101751234
-log10(p)
20
15
10
5
0
1
2
3
4
5
6
8
10
14
20
Chromosome

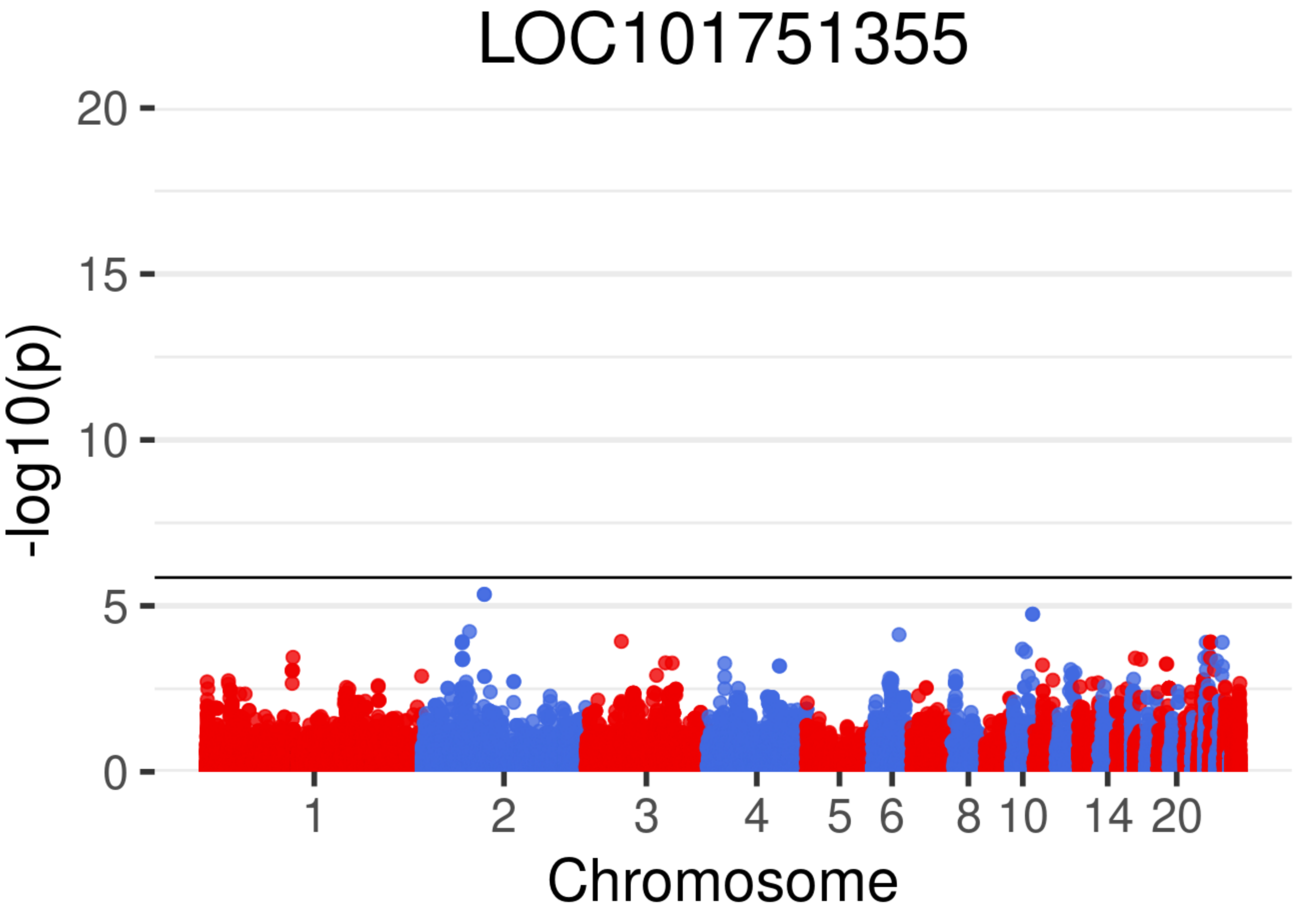
LOC101751355
20
15
10
5
0
-log10(p)
1
2
3
4
5
6
8
10
14
20
Chromosome

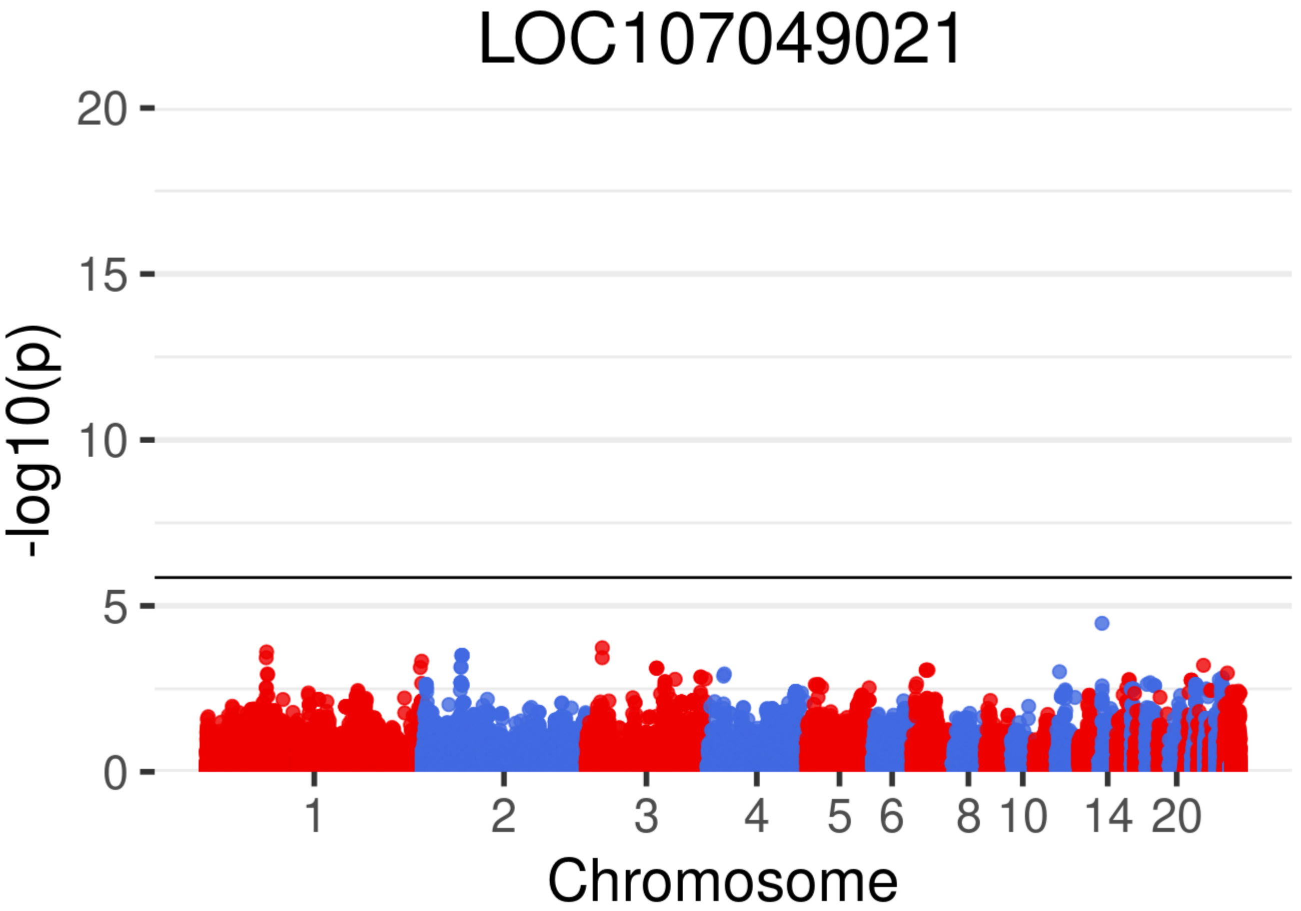
LOC107049021
-log10(p)
20
15
10
5
0
1
2
3
4
5
6
8
10
14
20
Chromosome

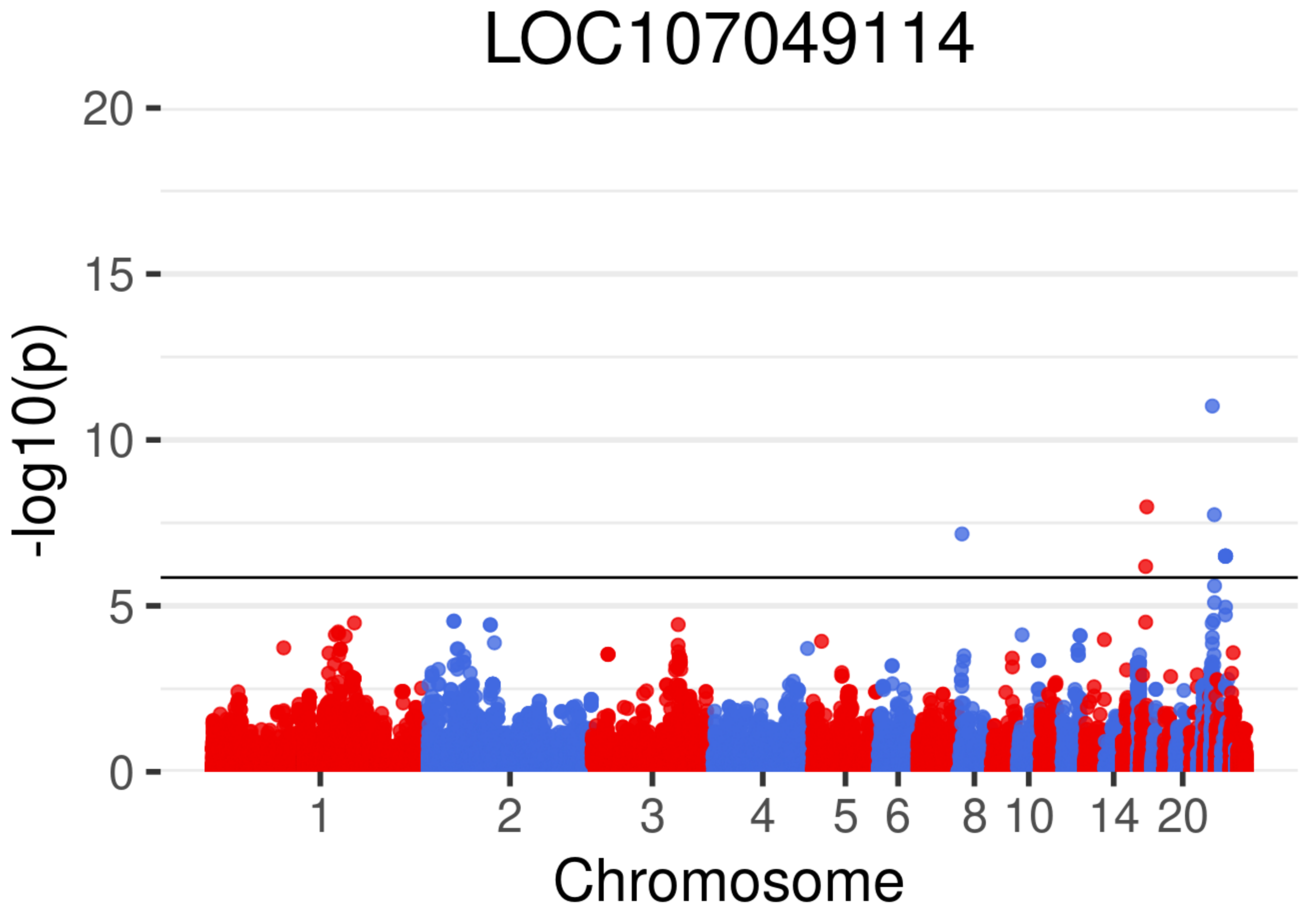

LOC107049114
-log10(p)
20
15
10
5
0
1
2
3
4
5
6
8
10
14
20
Chromosome

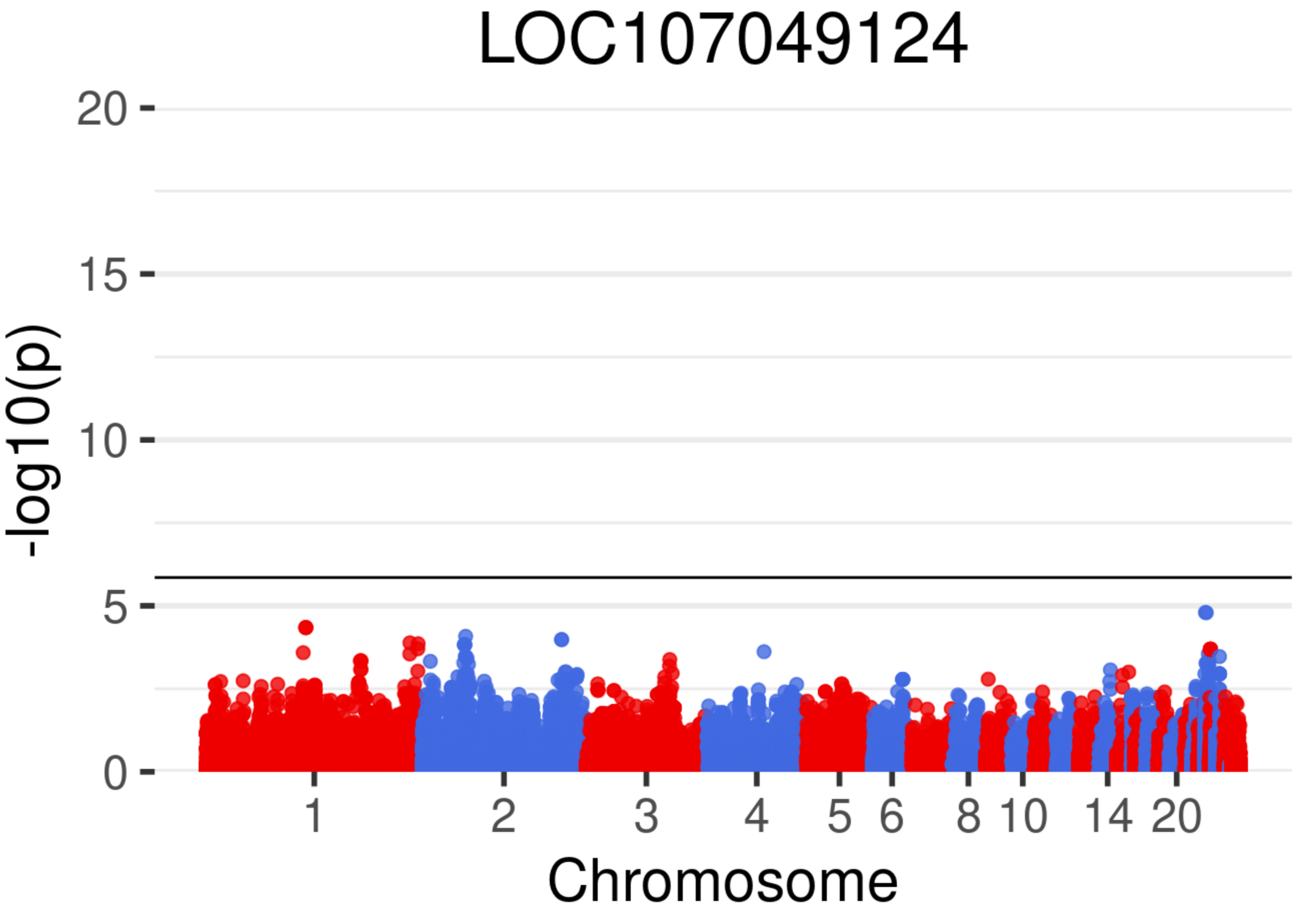
LOC107049124
-log10(p)
20
15
10
5
0
1
2
3
4
5
6
8
10
14
20
Chromosome

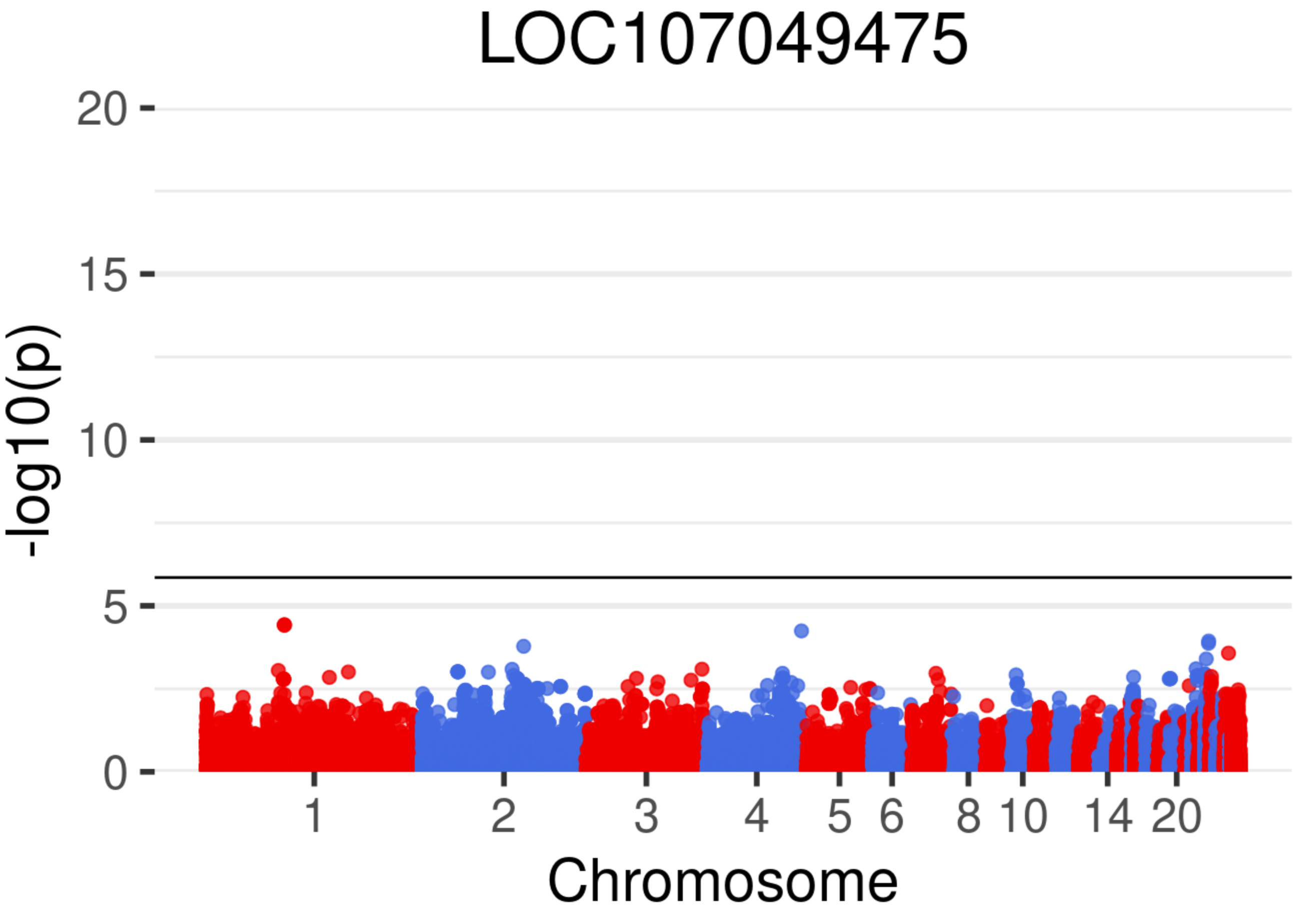

LOC107049475
20
15
10
5
0
-log10(p)
1
2
3
4
5
6
8
10
14
20
Chromosome

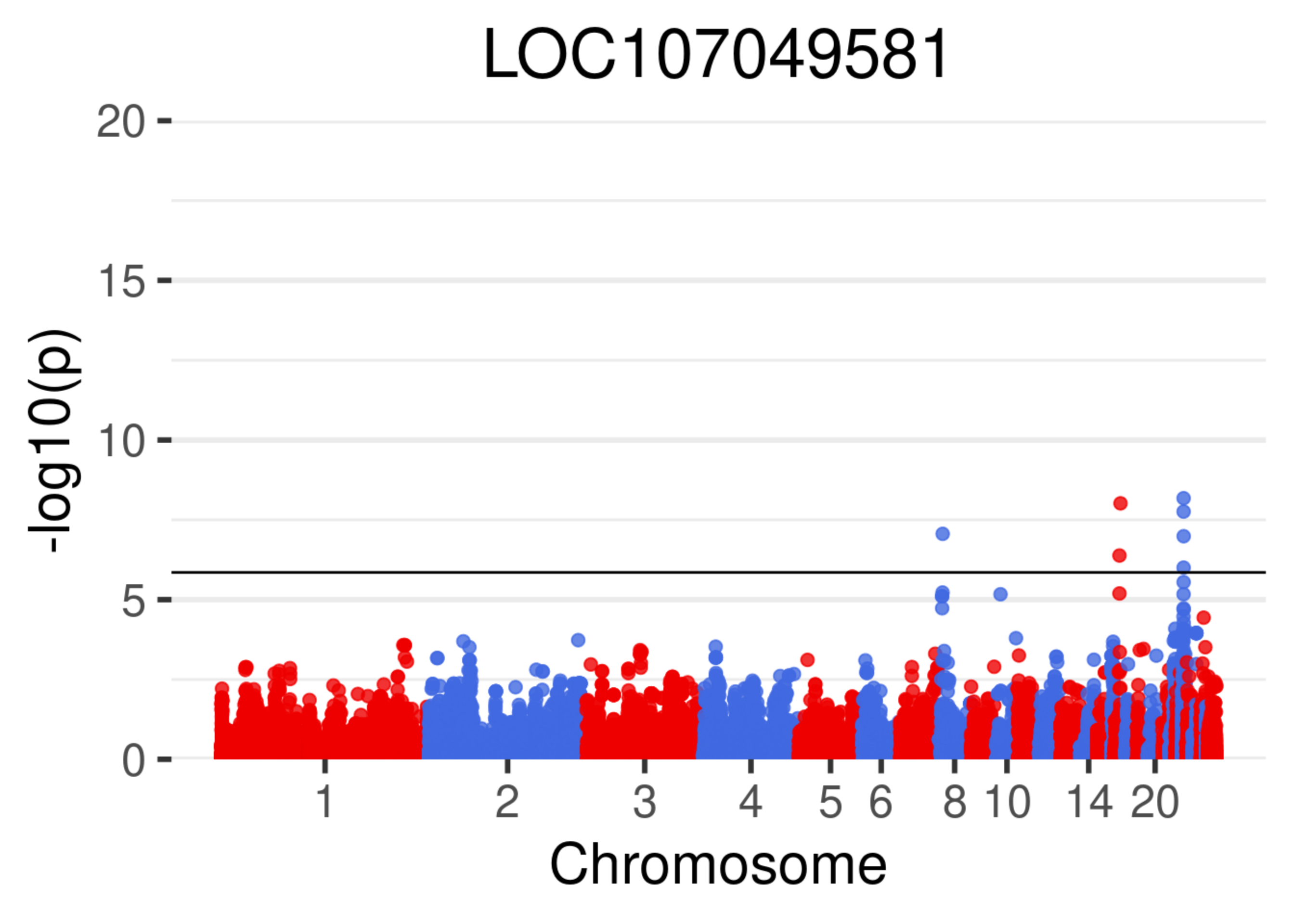

LOC107049581
-log10(p)
20
15
10
5
0
1
2
3
4
5
6
8
10
14
20
Chromosome

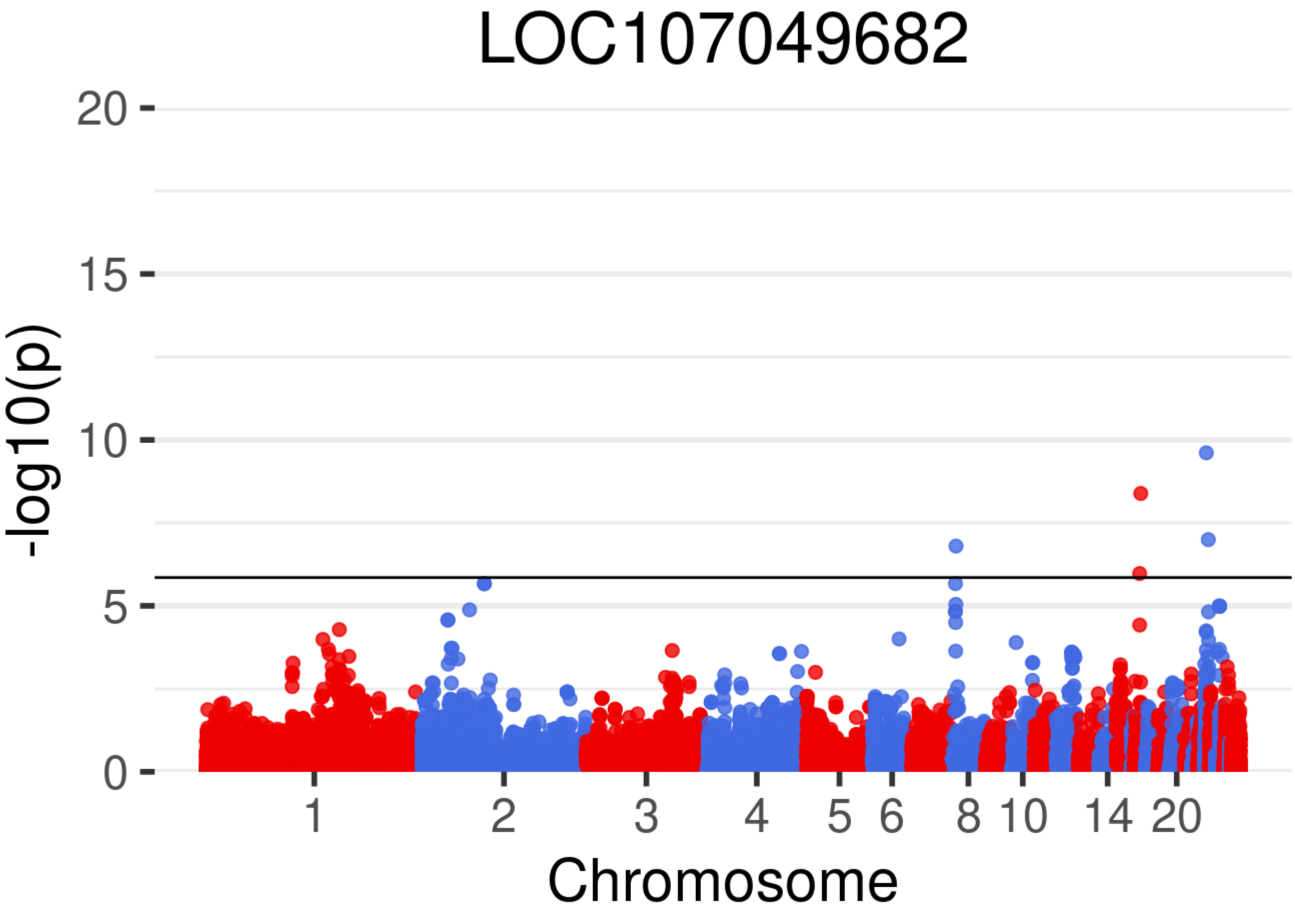
LOC107049682
20
15
10
5
0
-log10(p)
1
2
3
4
5
6
8
10
14
20
Chromosome

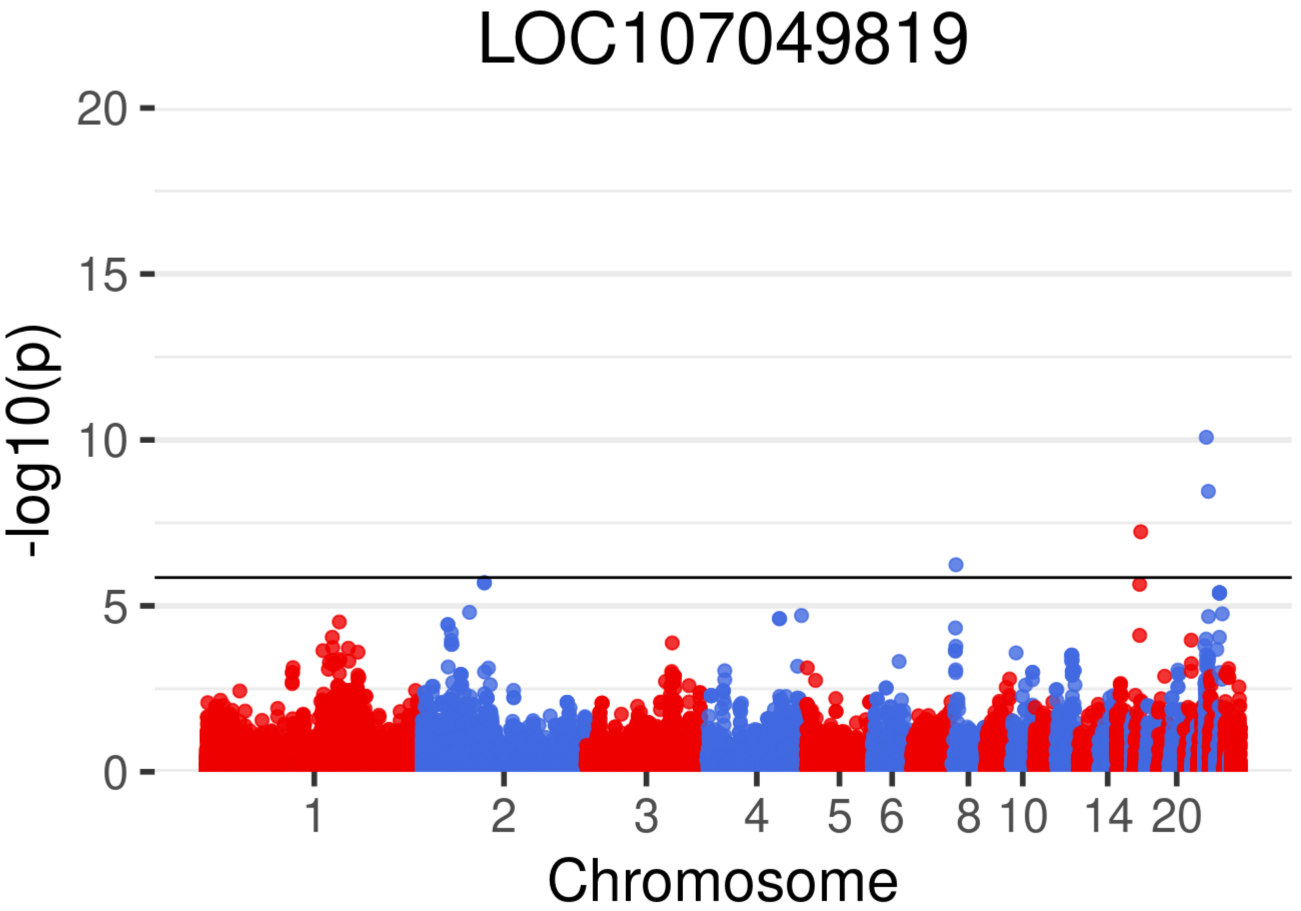
LOC107049819
-log10(p)
20
15
10
5
0
1
2
3
4
5
6
8
10
14
20
Chromosome

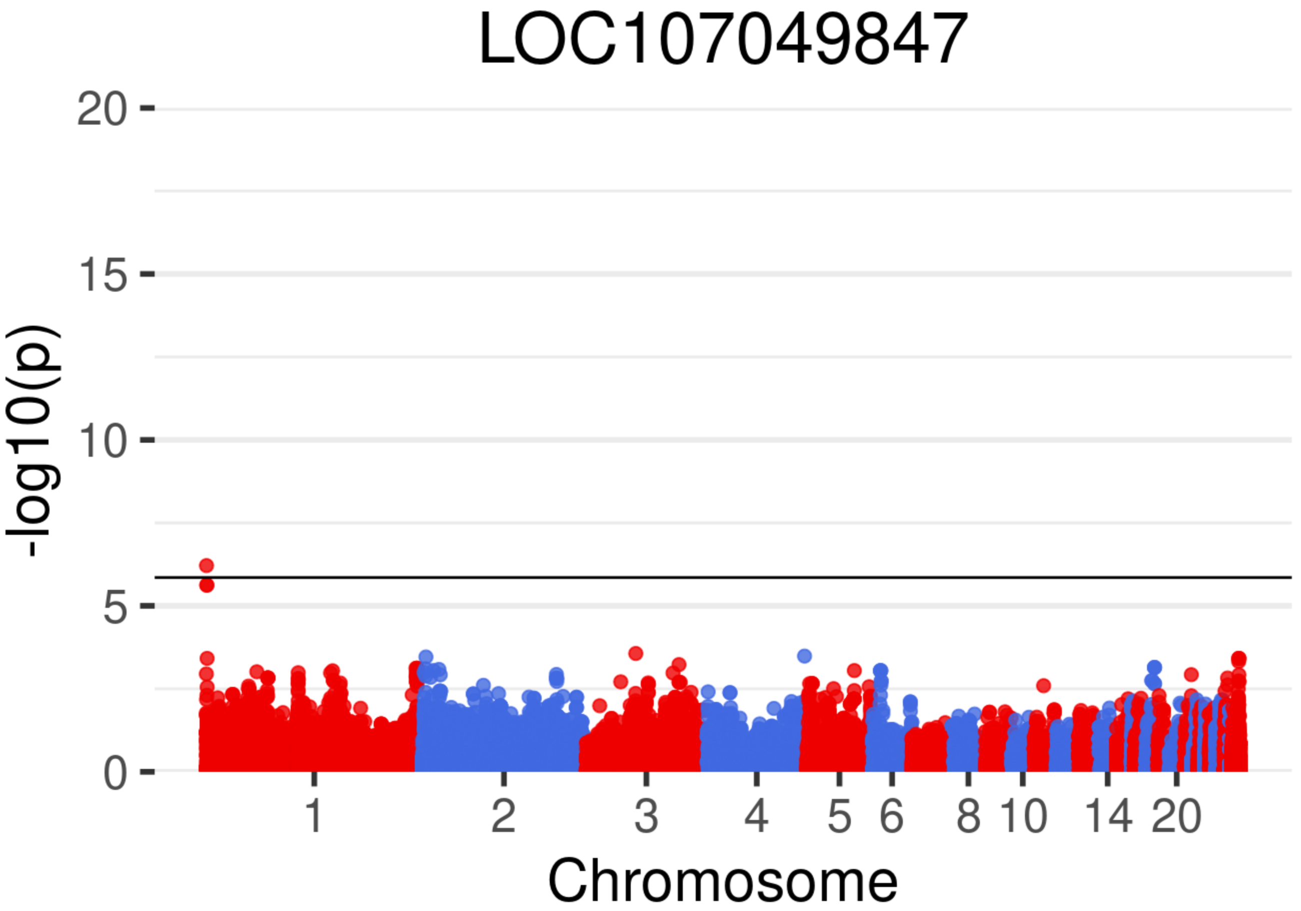

LOC107049847
-log10(p)
20
15
10
5
0
1
2
3
4
5
6
8
10
14
20
Chromosome

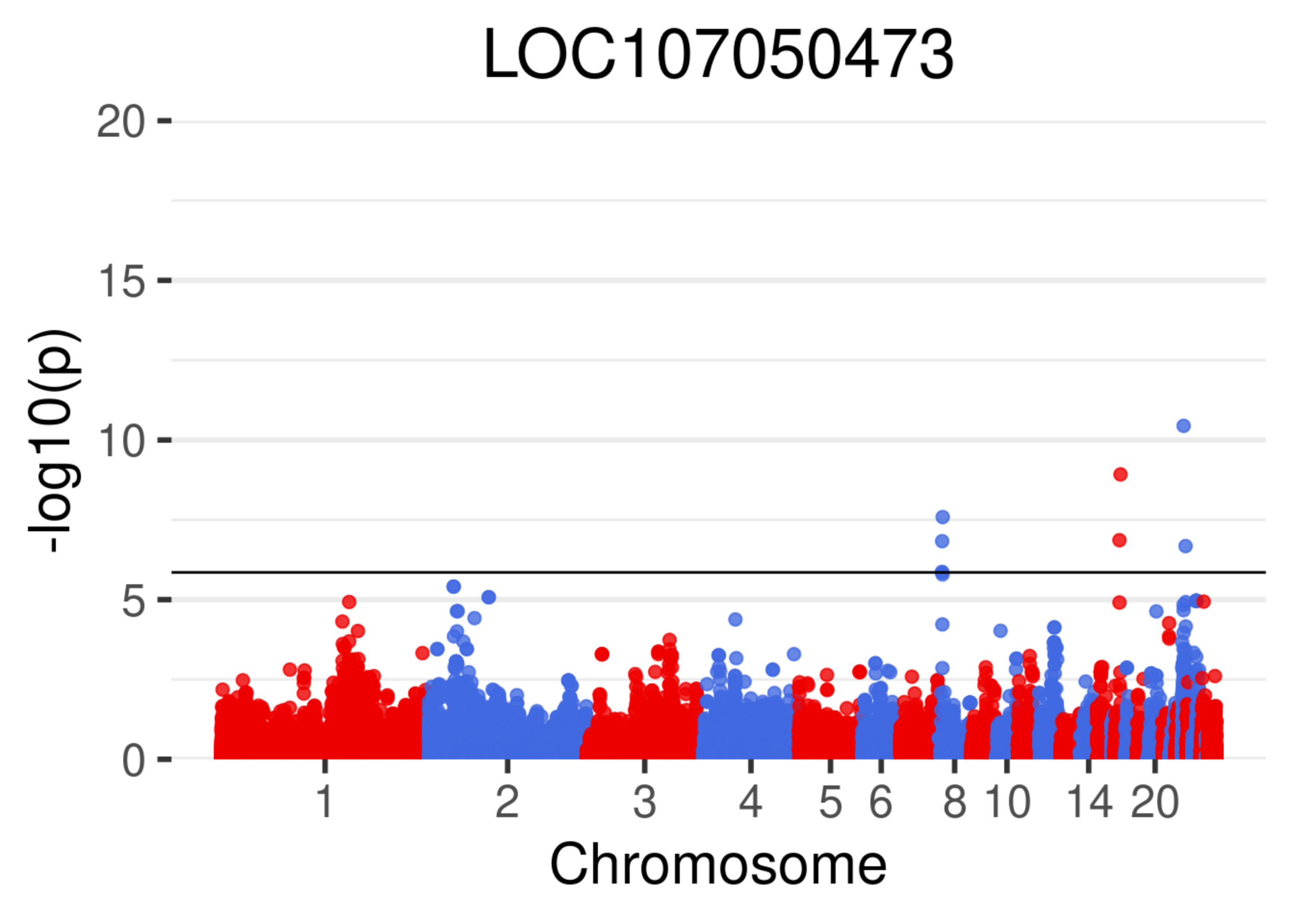
LOC107050473
-log10(p)
20
15
10
5
0
1
2
3
4
5
6
8
10
14
20
Chromosome

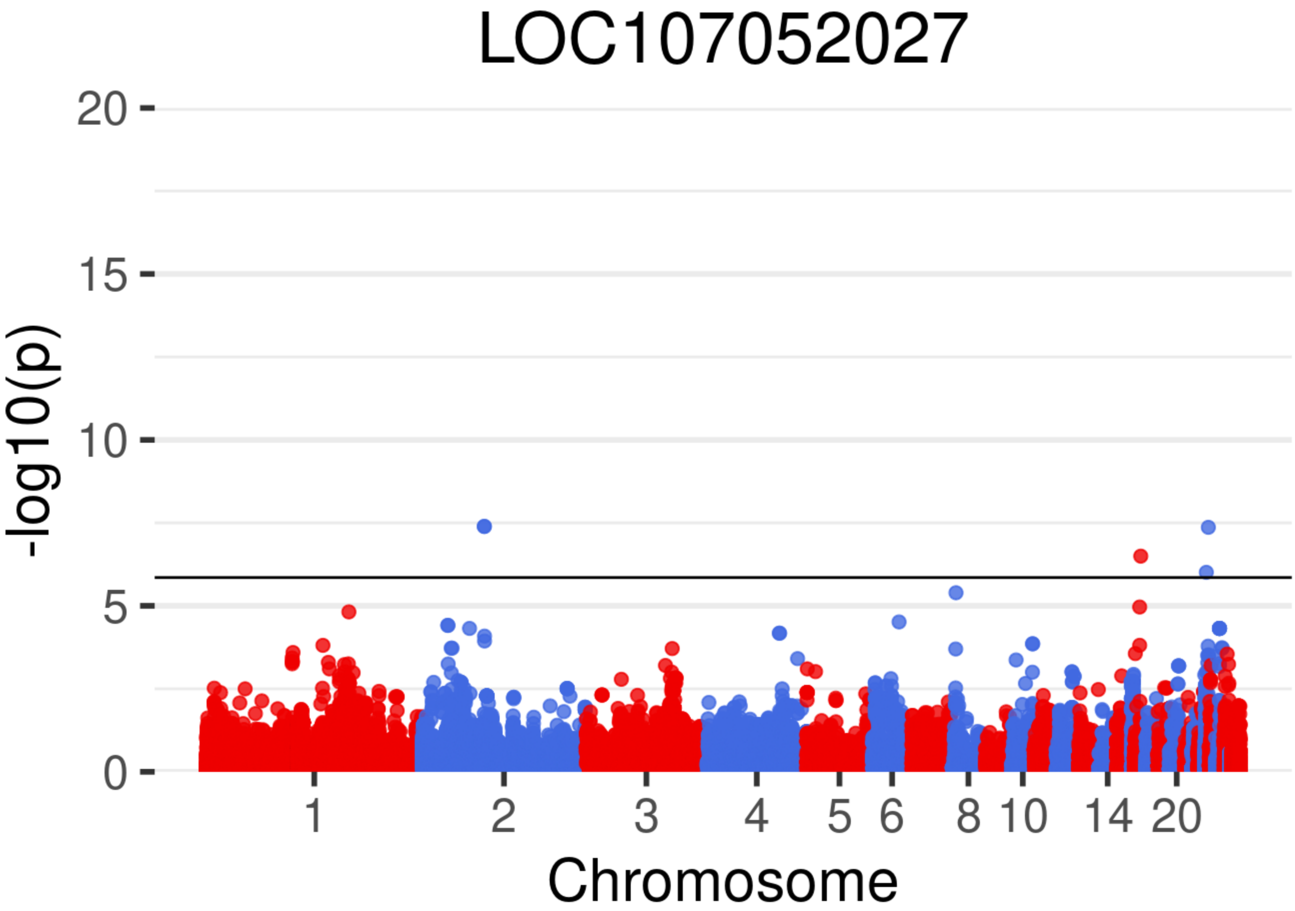

LOC107052027
20
15
10
5
0
-log10(p)
1
2
3
4
5
6
8
10
14
20
Chromosome

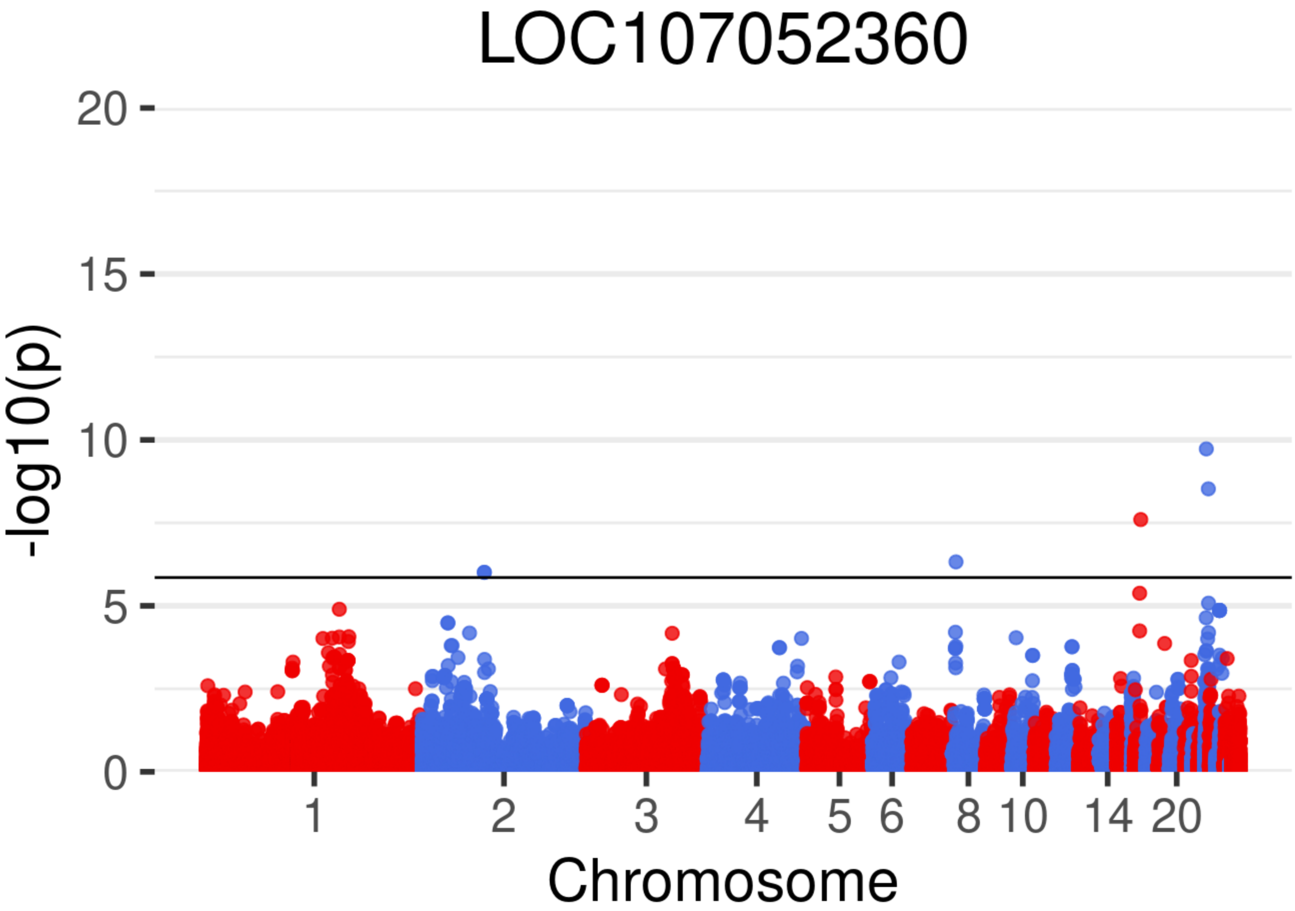

LOC107052360
-log10(p)
20
15
10
5
0
1
2
3
4
5
6
8
10
14
20
Chromosome

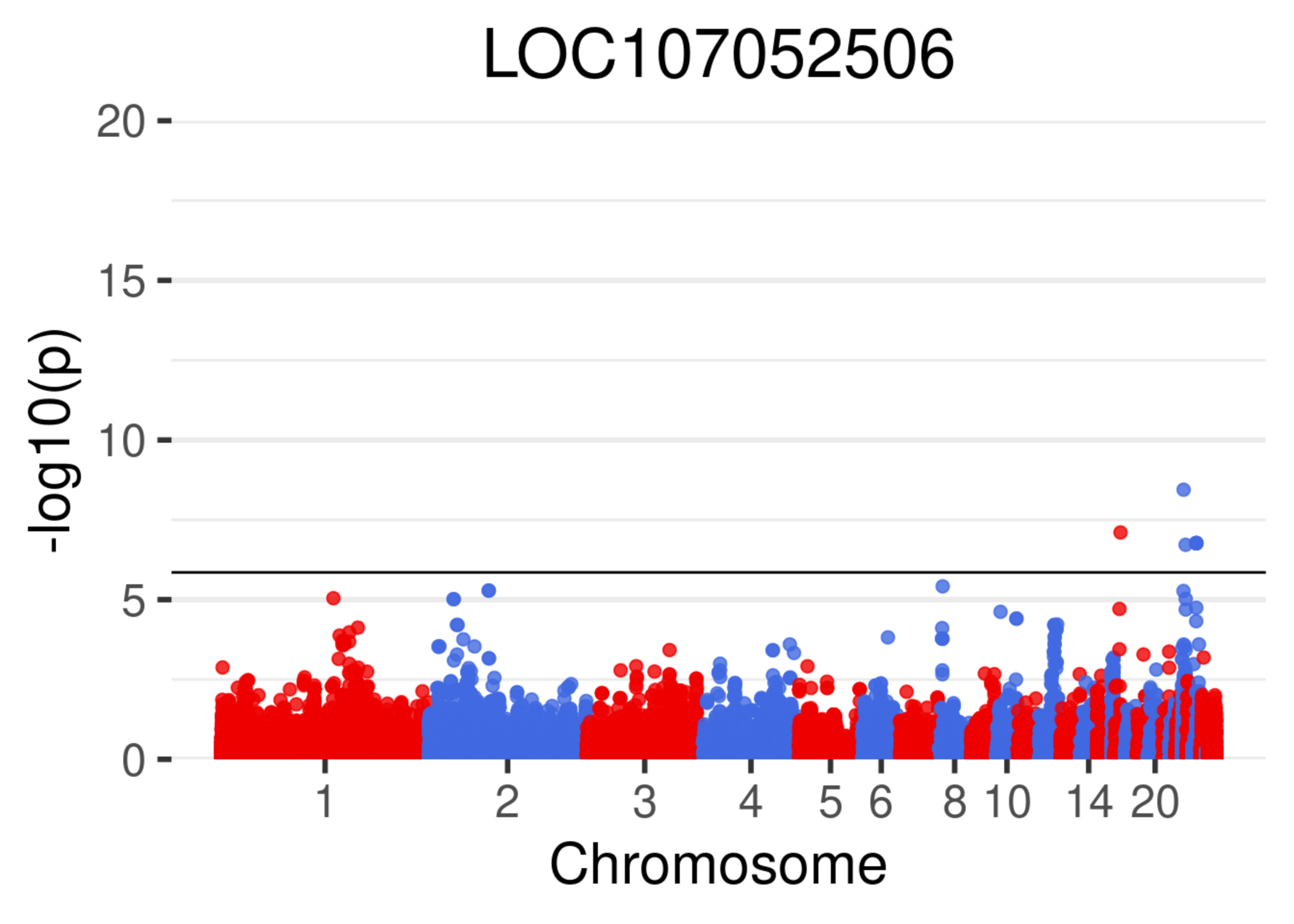

LOC107052506
-log10(p)
20
15
10
5
0
1
2
3
4
5
6
8
10
14
20
Chromosome

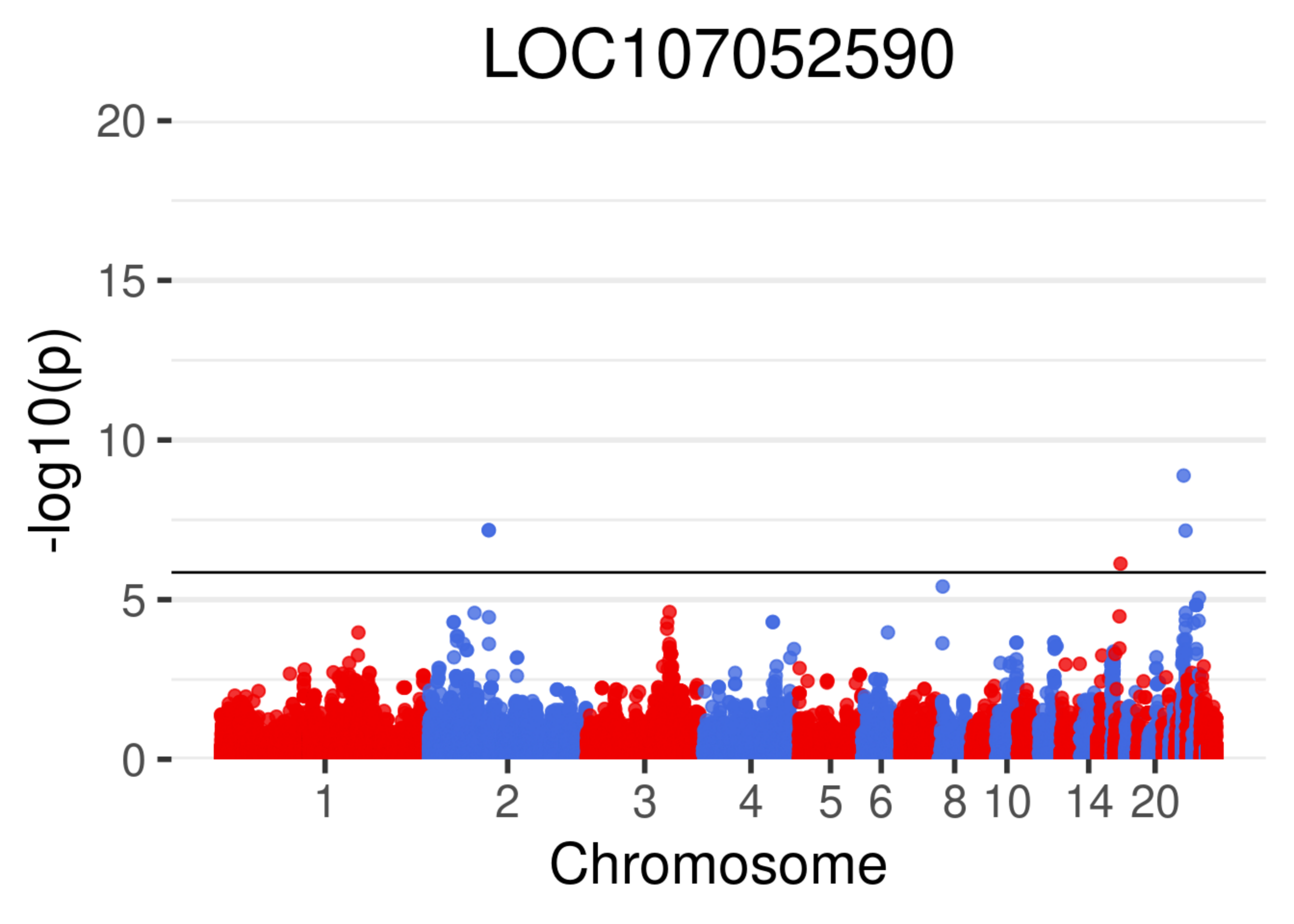
LOC107052590
-log10(p)
0
5
10
15
20
1
2
3
4
5
6
8
10
14
20
Chromosome

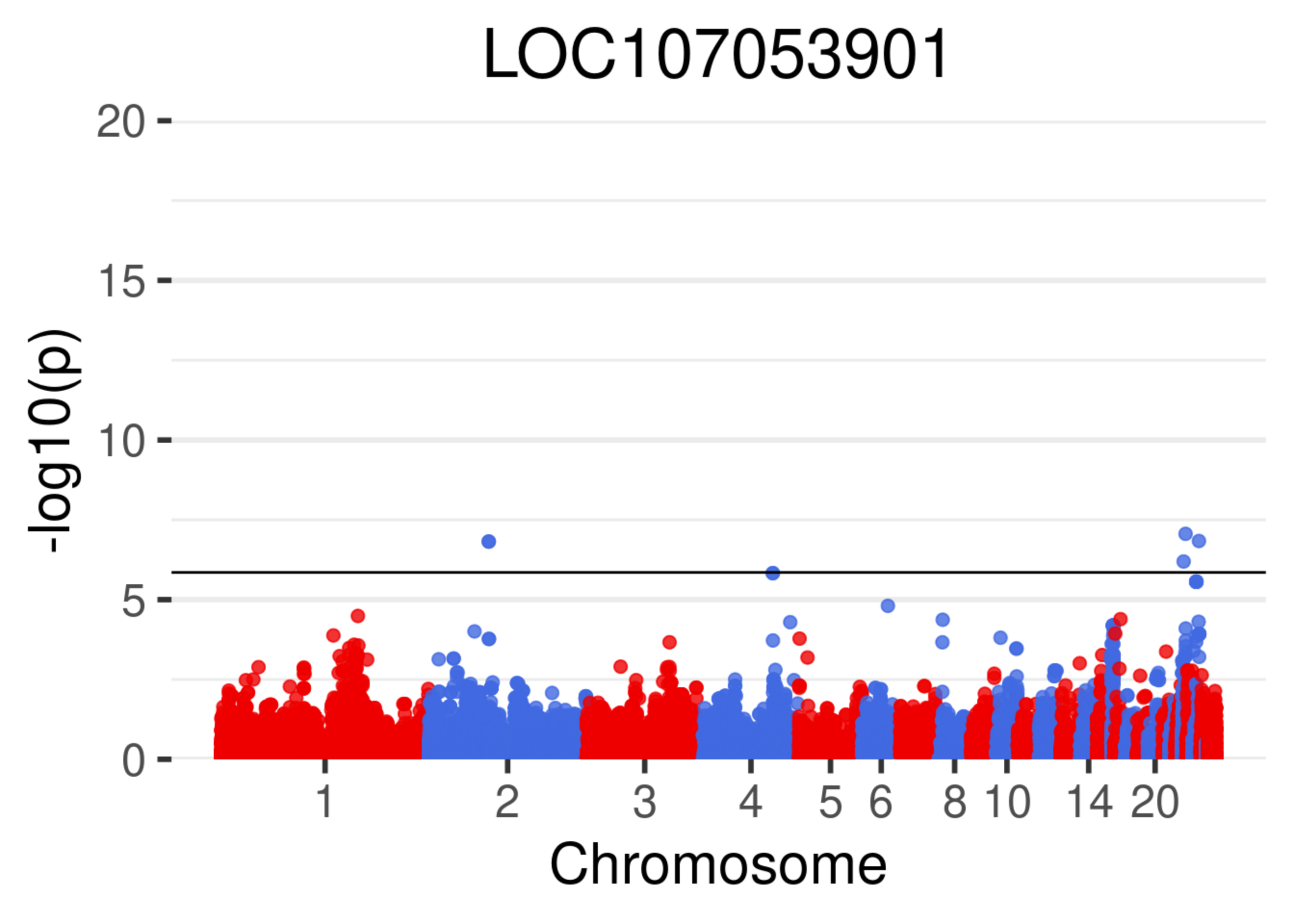

LOC107053901
-log10(p)
20
15
10
5
0
1
2
3
4
5
6
8
10
14
20
Chromosome

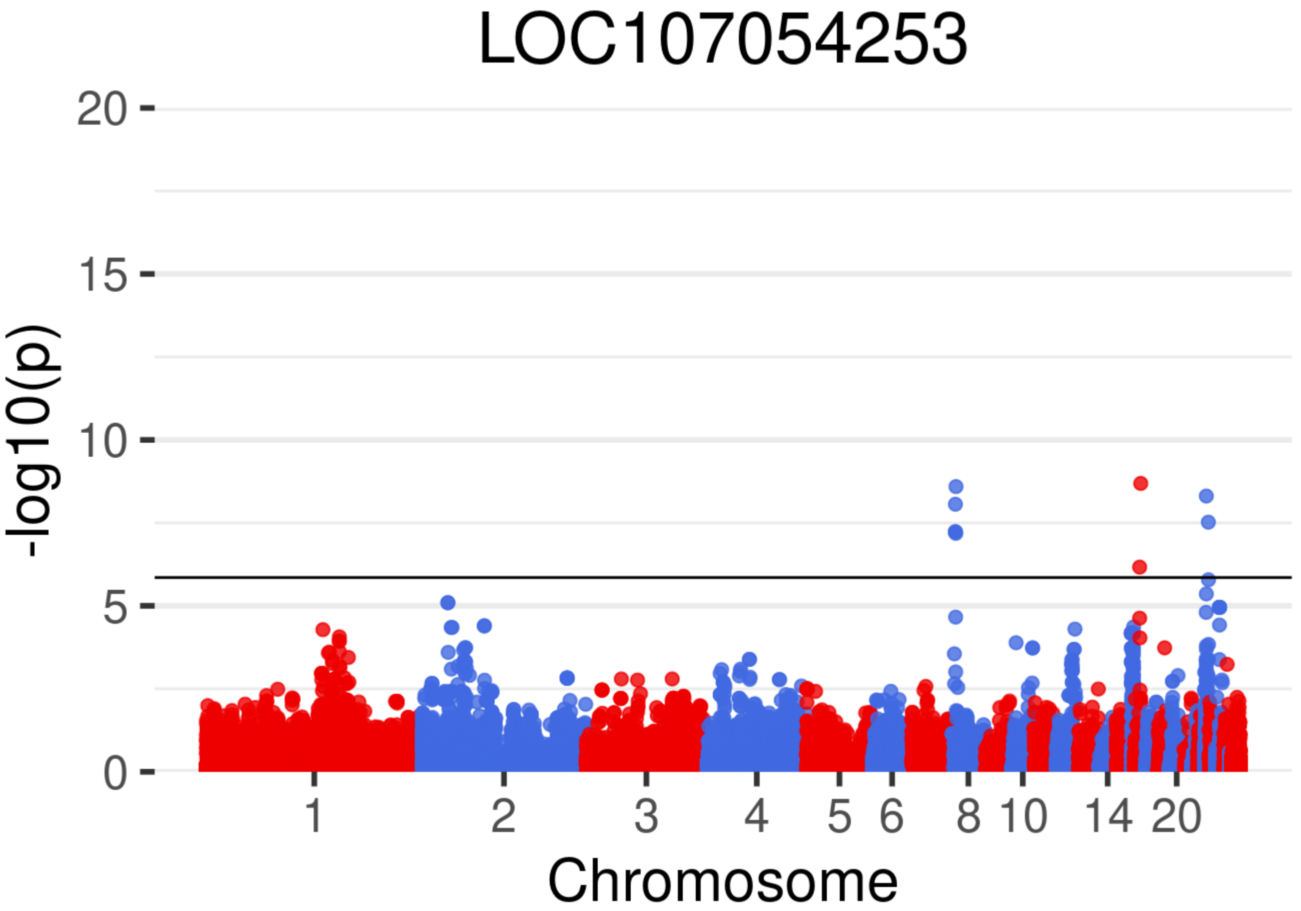

LOC107054253
-log10(p)
20
15
10
5
0
1
2
3
4
5
6
8
10
14
20
Chromosome

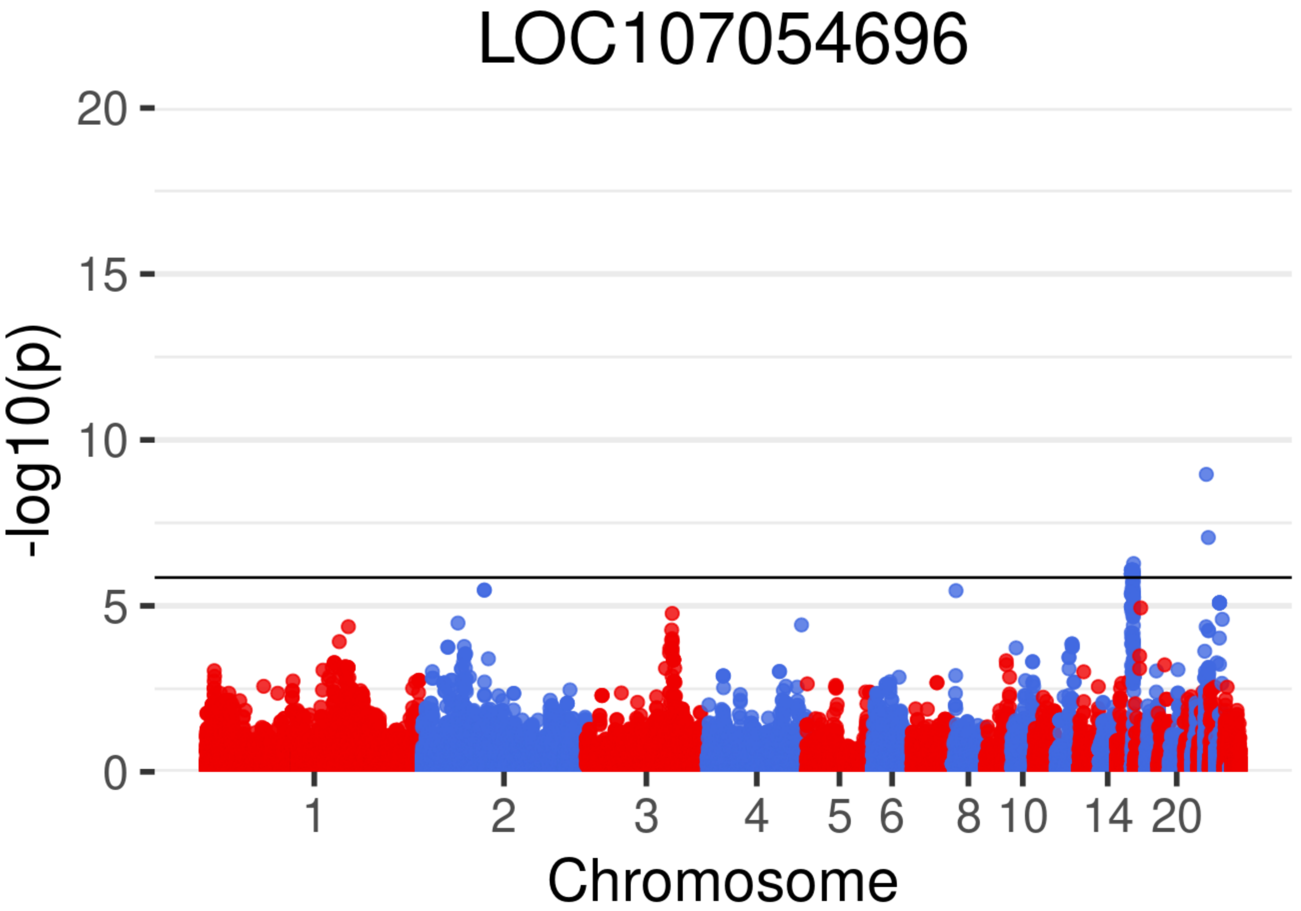

LOC107054696
-log10(p)
20
15
10
5
0
1
2
3
4
5
6
8
10
14
20
Chromosome

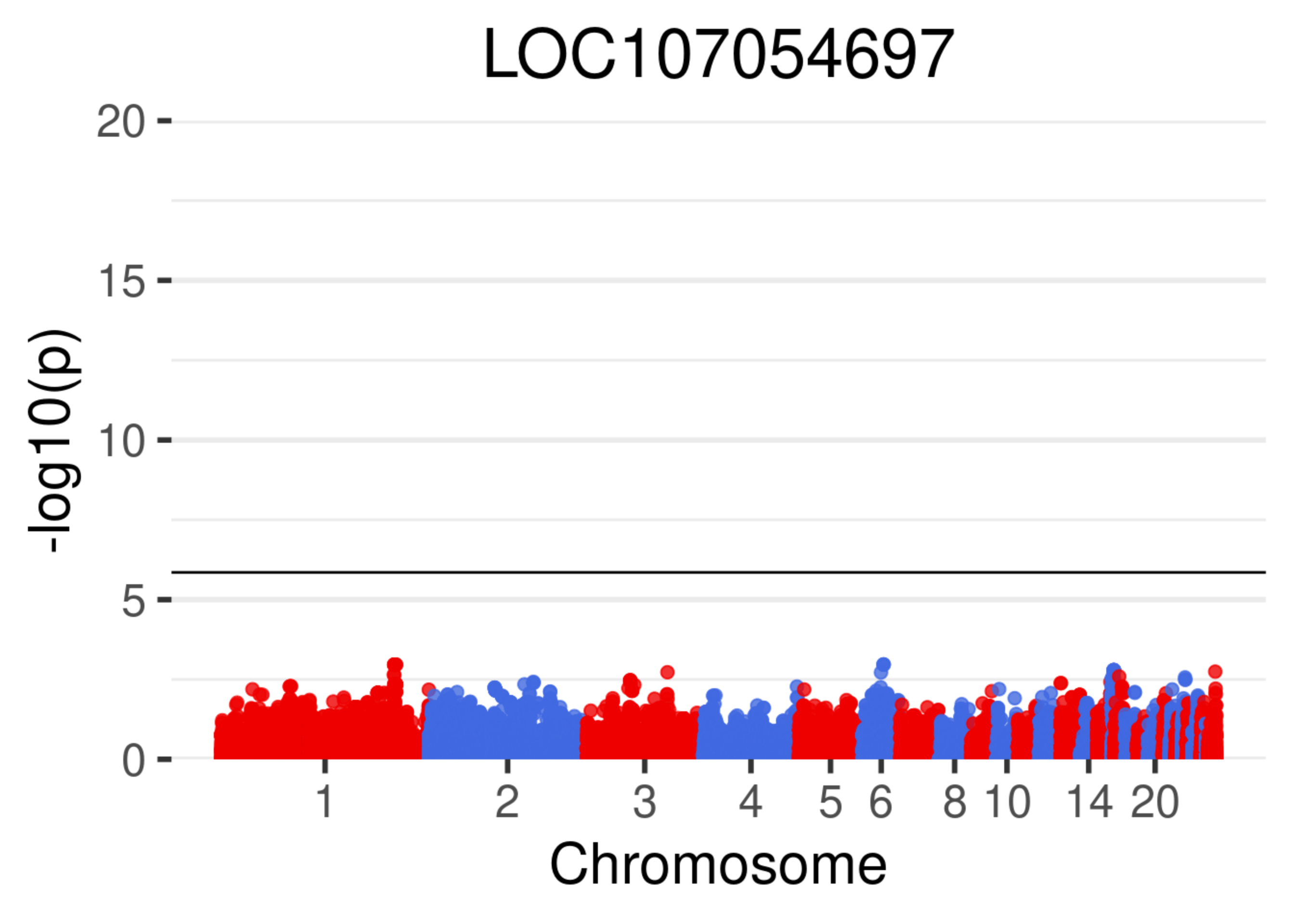
LOC107054697
20
15
10
5
0
-log10(p)
1
2
3
4
5
6
8
10
14
20
Chromosome

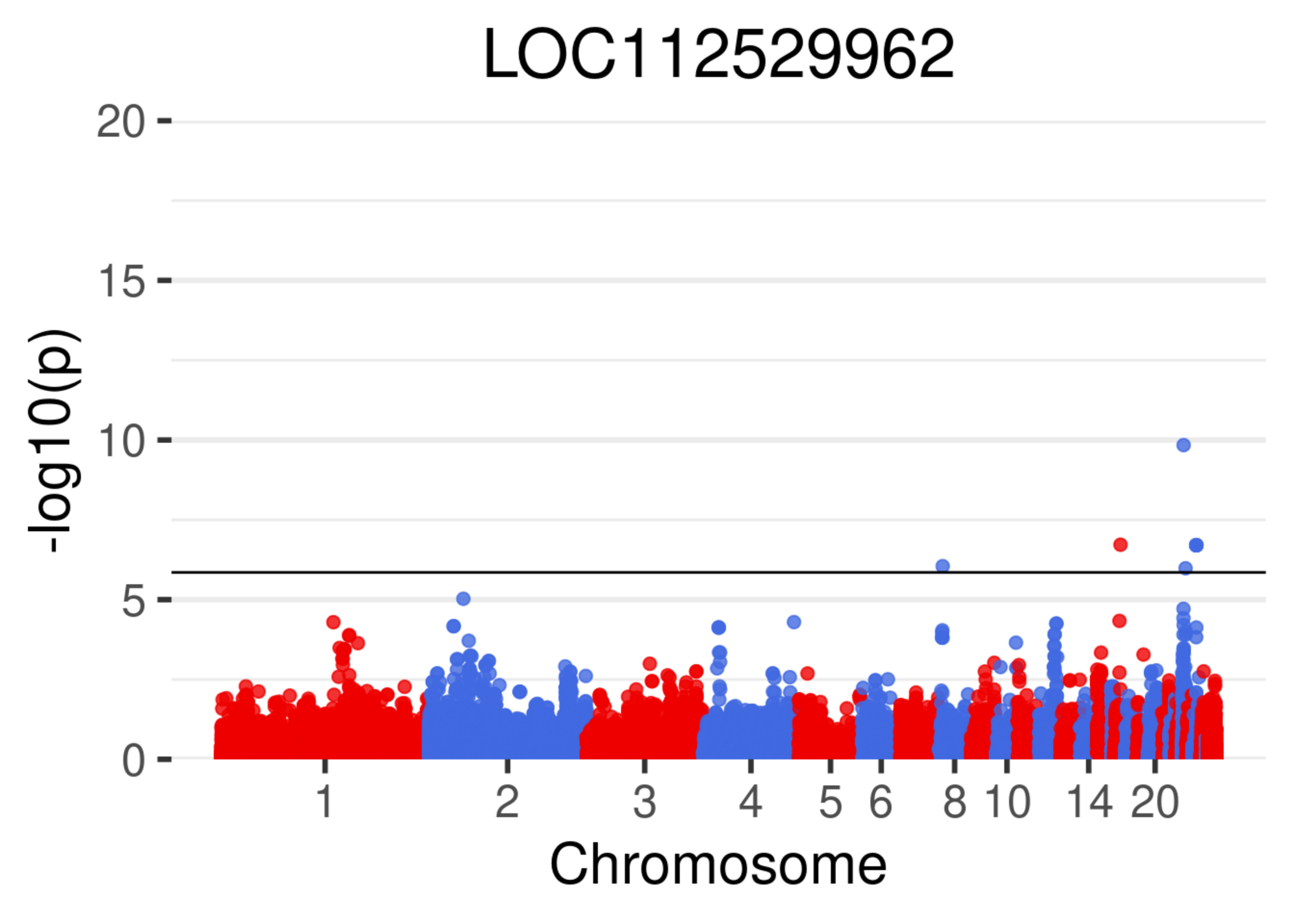

LOC112529962
-log10(p)
20
15
10
5
0
1
2
3
4
5
6
8
10
14
20
Chromosome

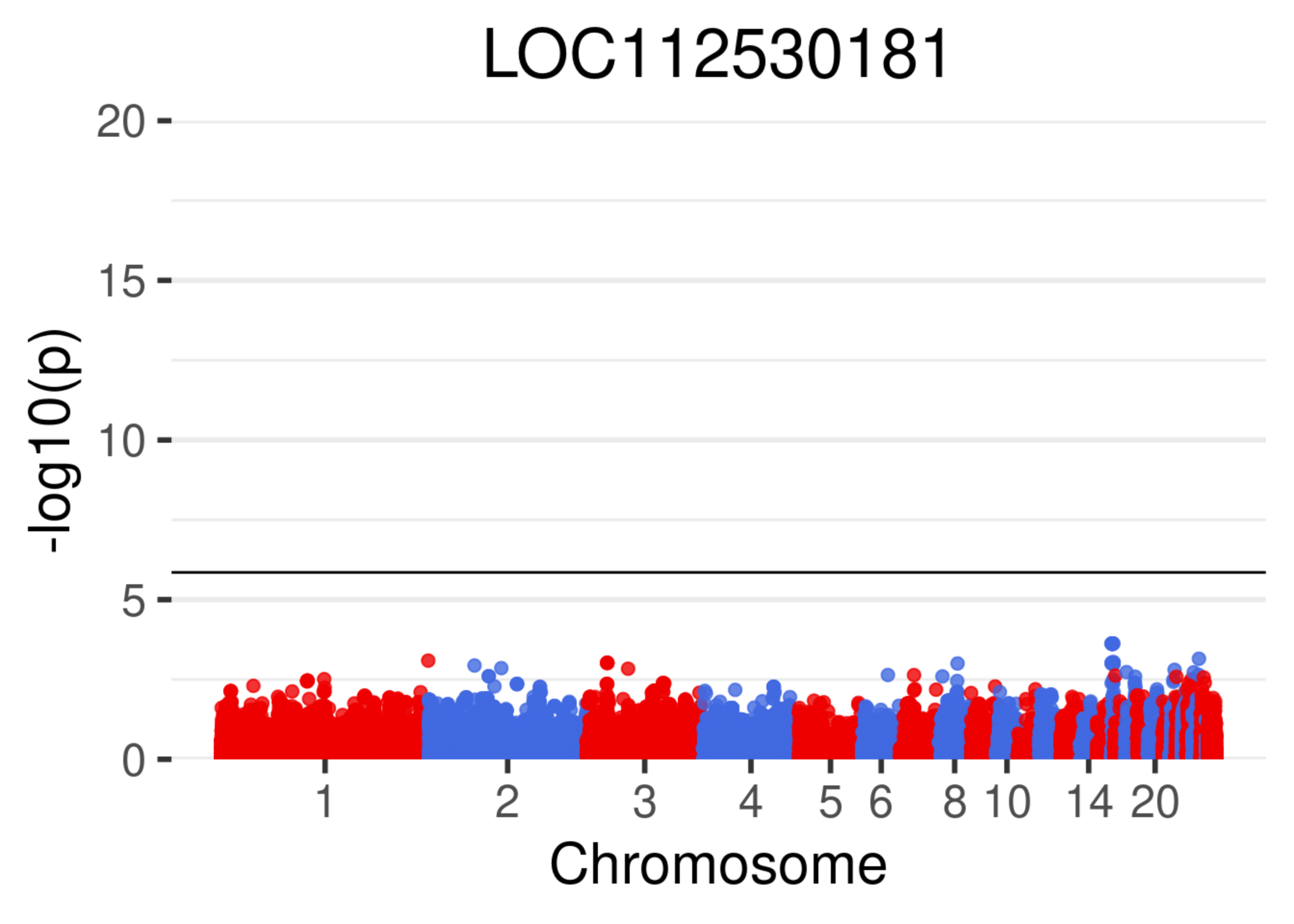
LOC112530181
20
15
10
5
0
-log10(p)
1
2
3
4
5
6
8
10
14
20
Chromosome

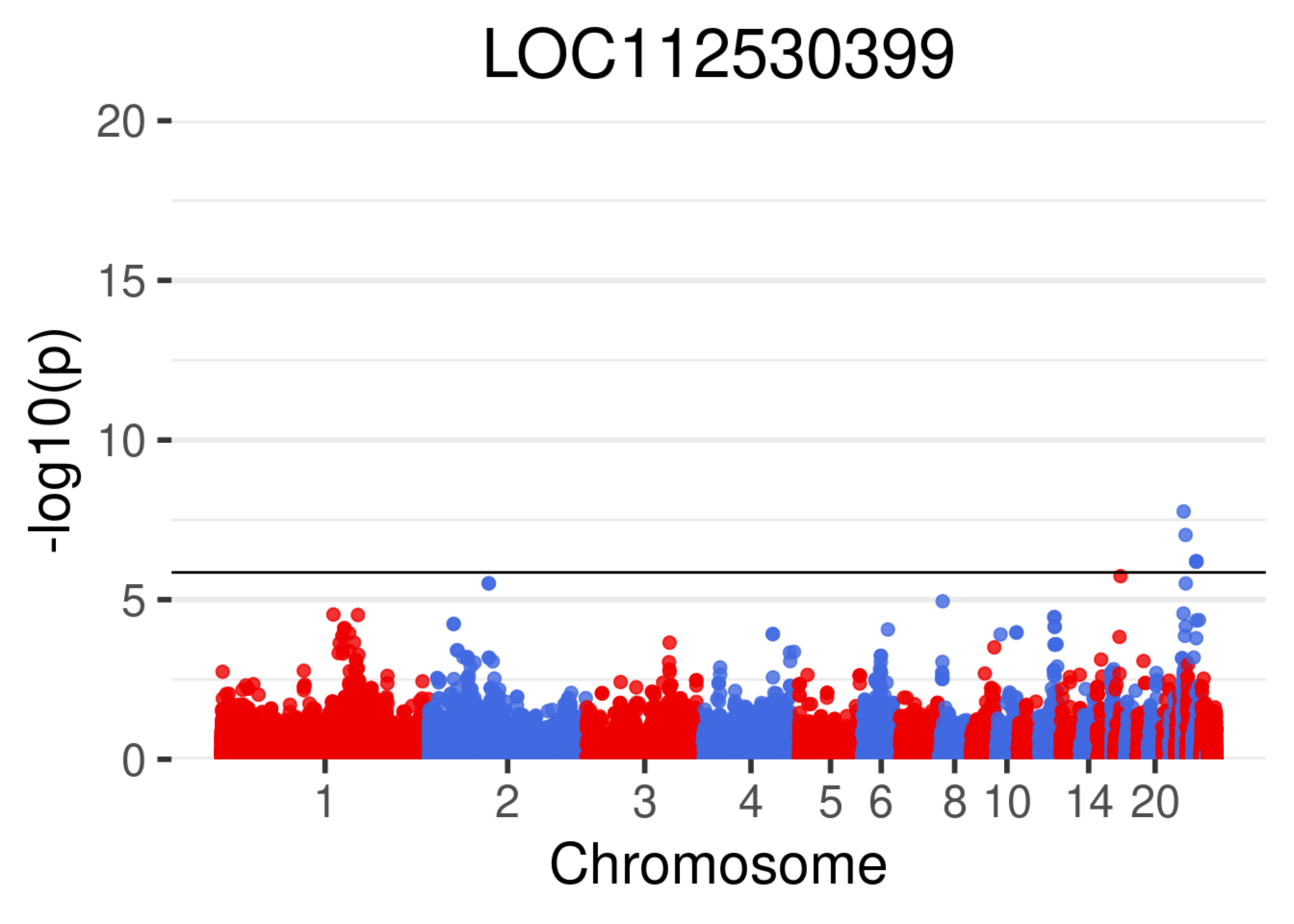
LOC112530399
-log10(p)
20
15
10
5
0
1
2
3
4
5
6
8
10
14
20
Chromosome

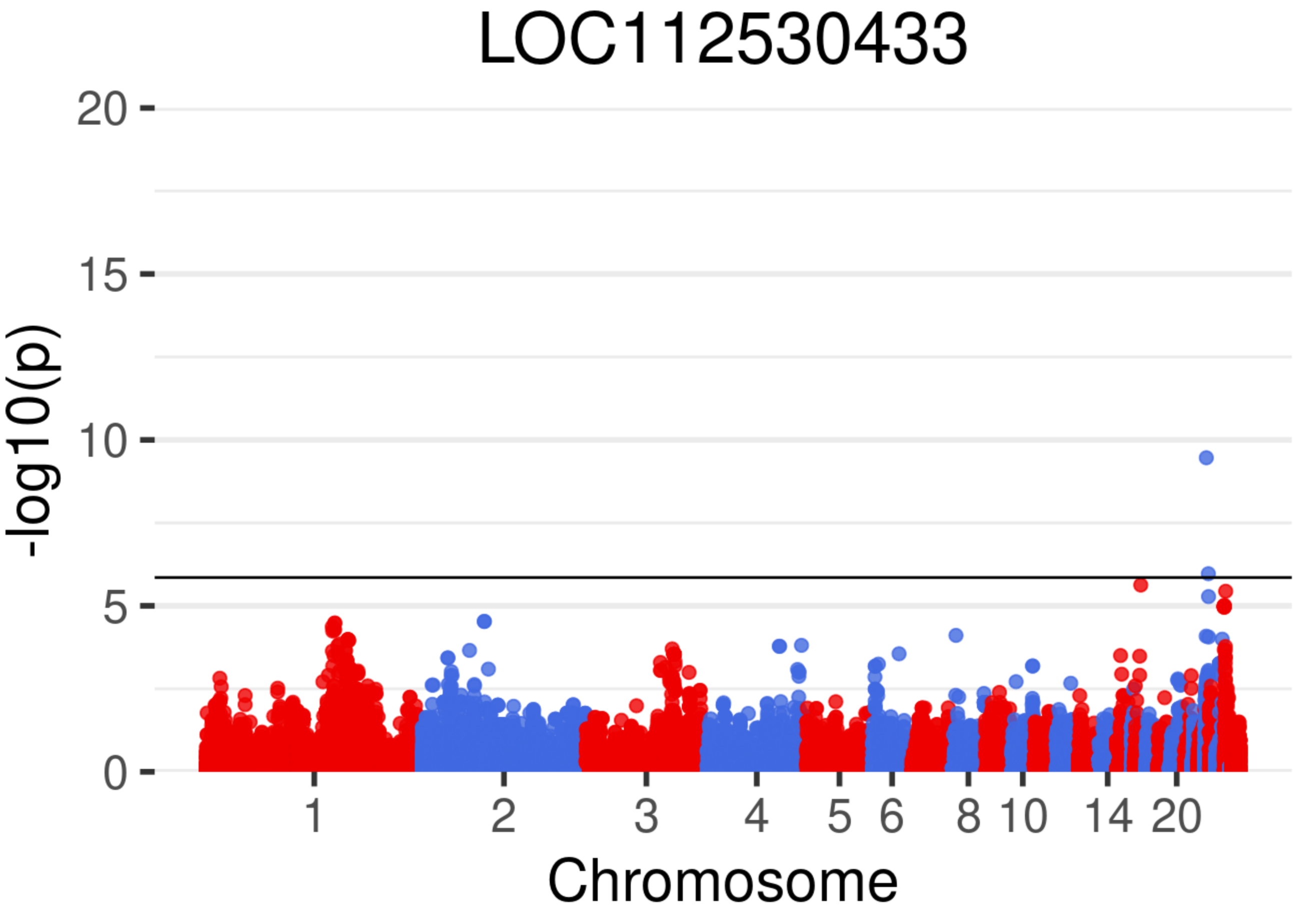
LOC112530433
20
15
10
5
0
-log10(p)
1
2
3
4
5
6
8
10
14
20
Chromosome

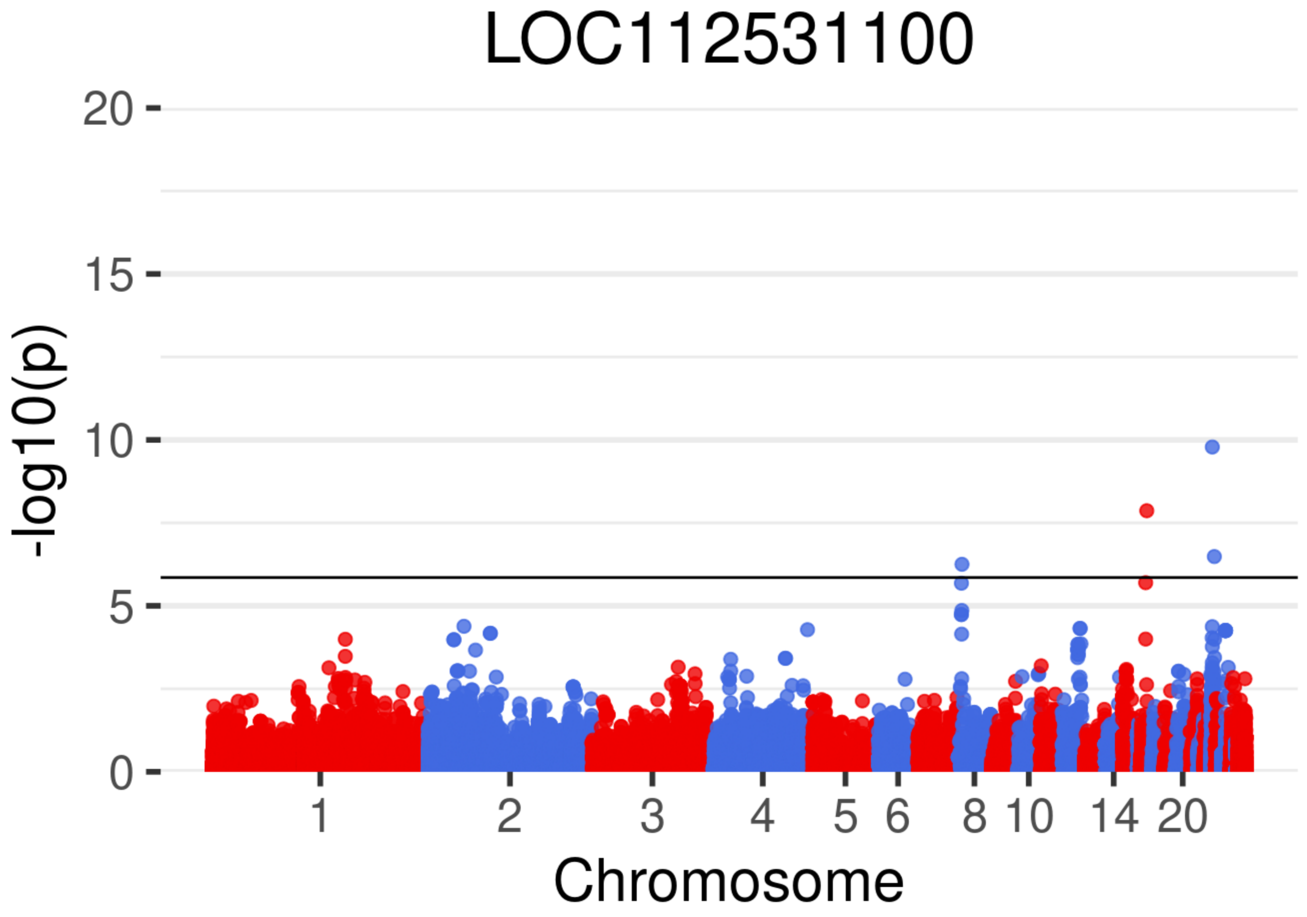
LOC112531100
-log10(p)
20
15
10
5
0
1
2
3
4
5
6
8
10
14
20
Chromosome

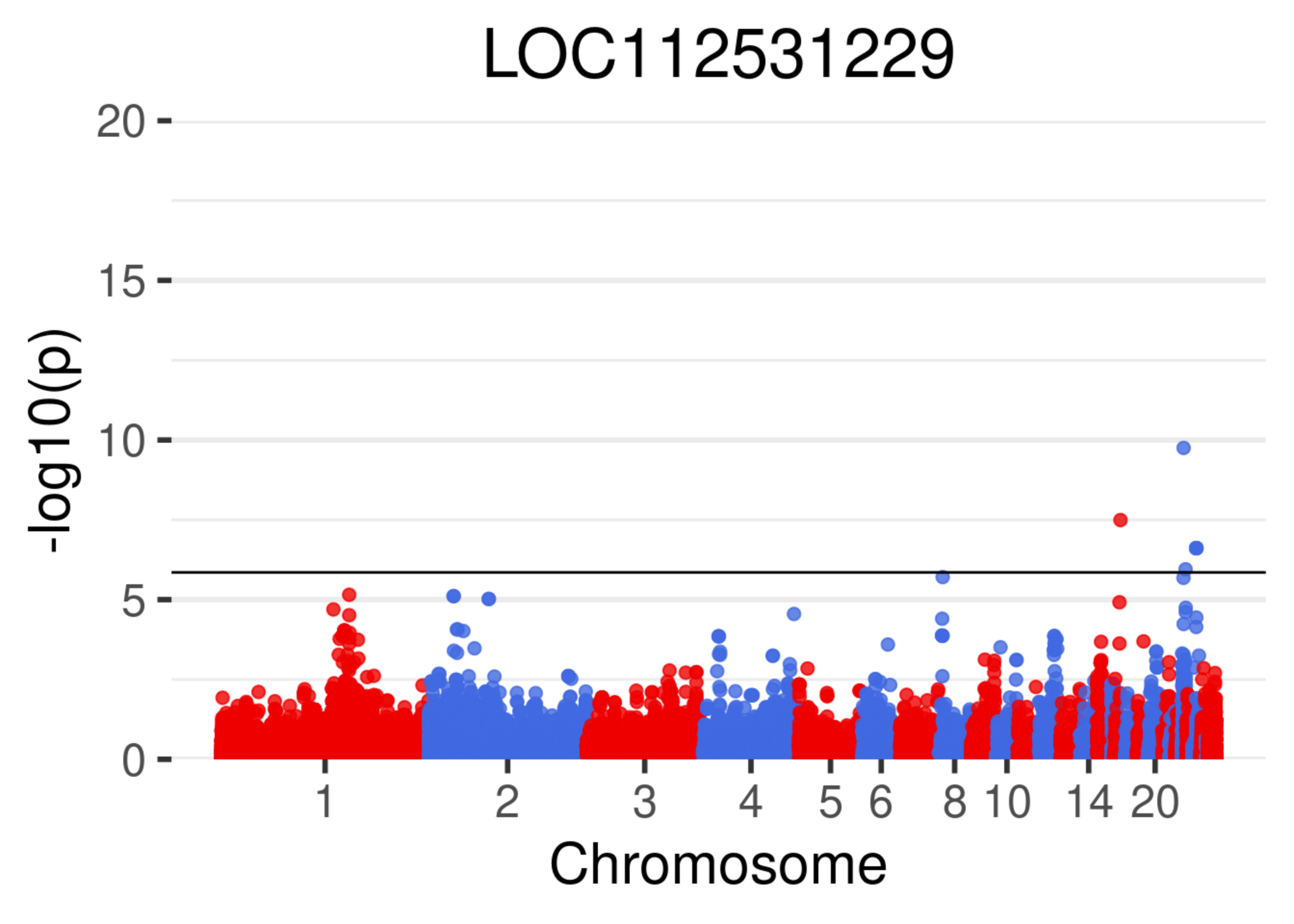
LOC112531229
-log10(p)
20
15
10
5
0
1
2
3
4
5
6
8
10
14
20
Chromosome

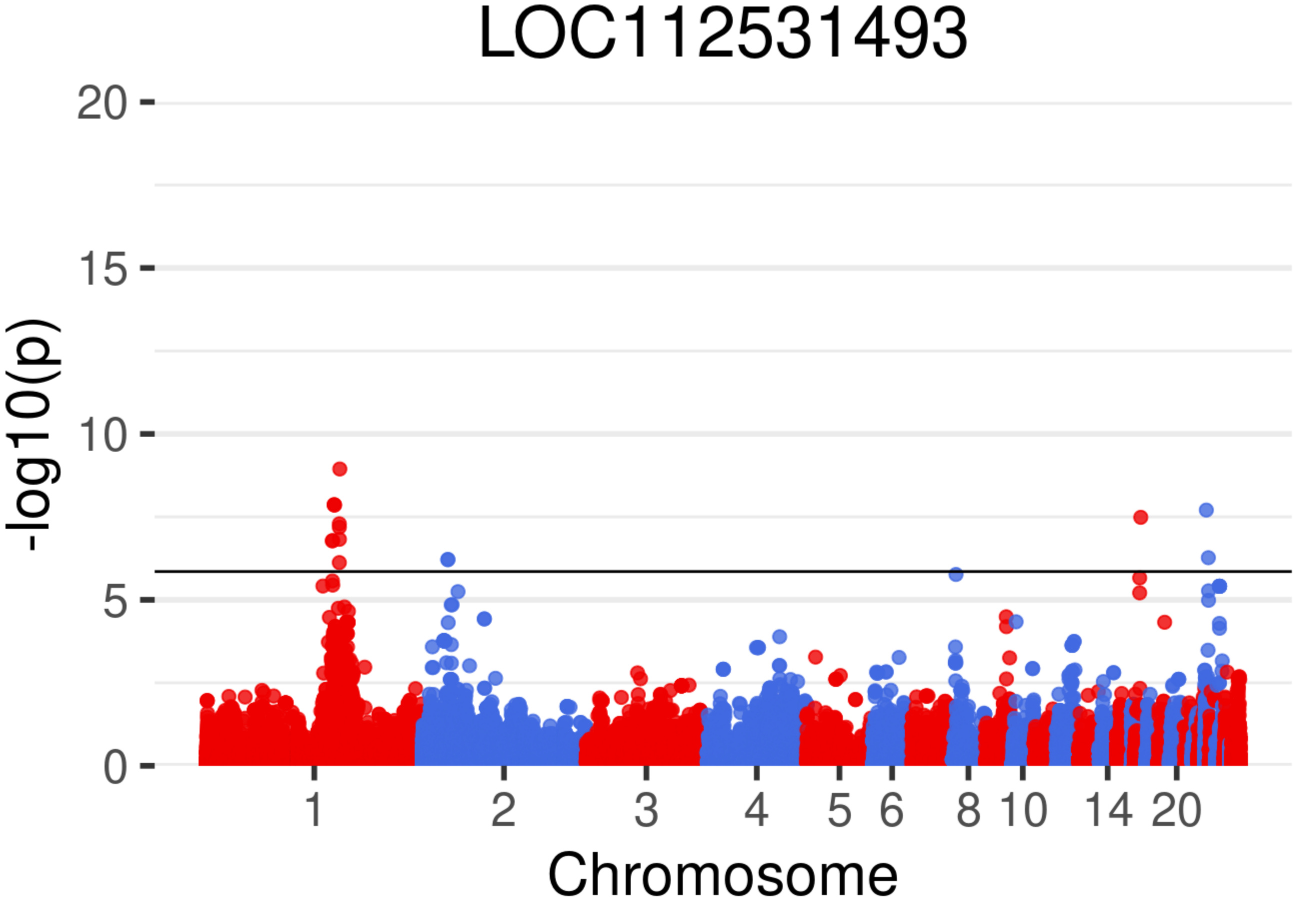
LOC112531493
-log10(p)
20
15
10
5
0
1
2
3
4
5
6
8
10
14
20
Chromosome

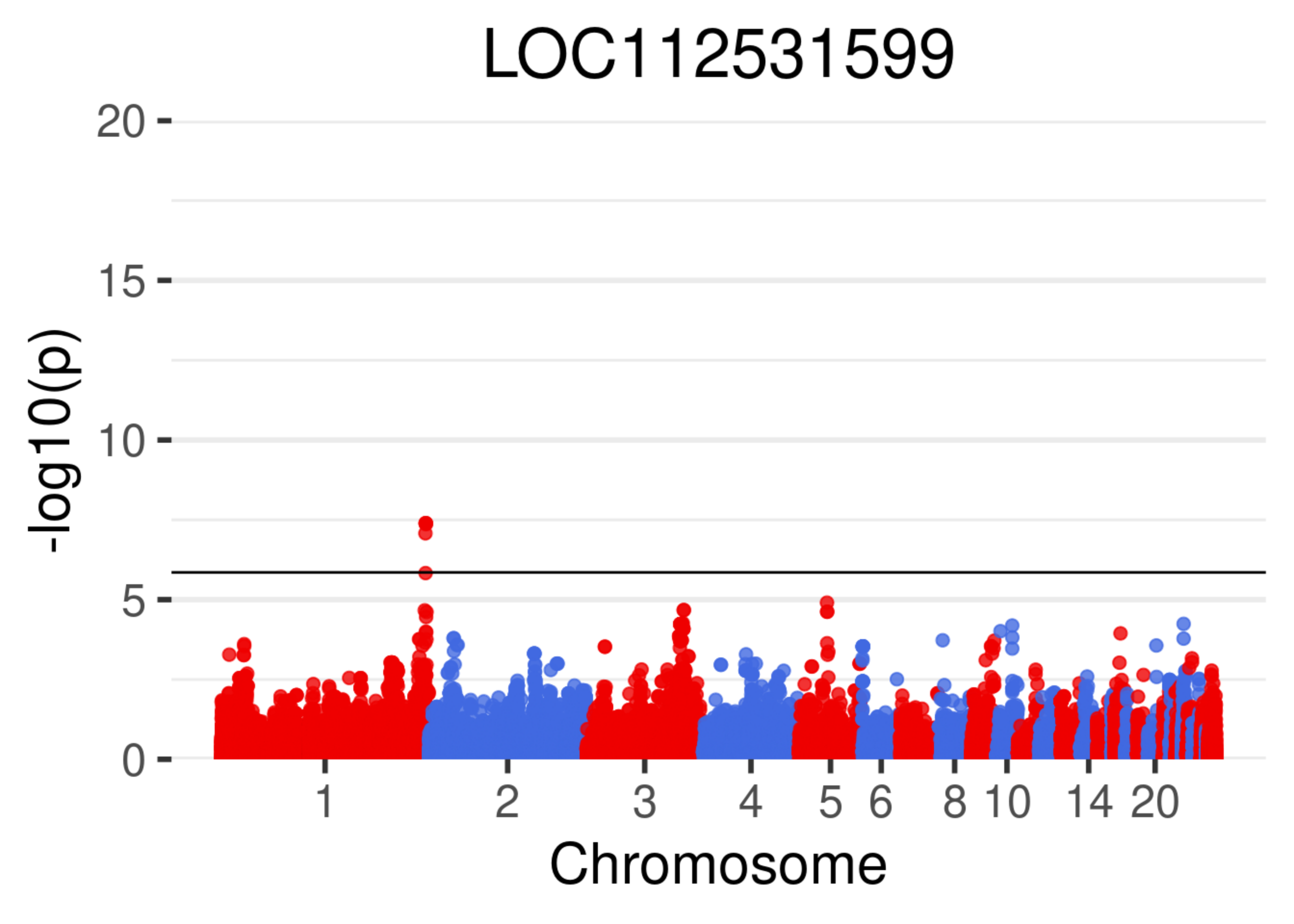
LOC112531599
-log10(p)
20
15
10
5
0
1
2
3
4
5
6
8
10
14
20
Chromosome

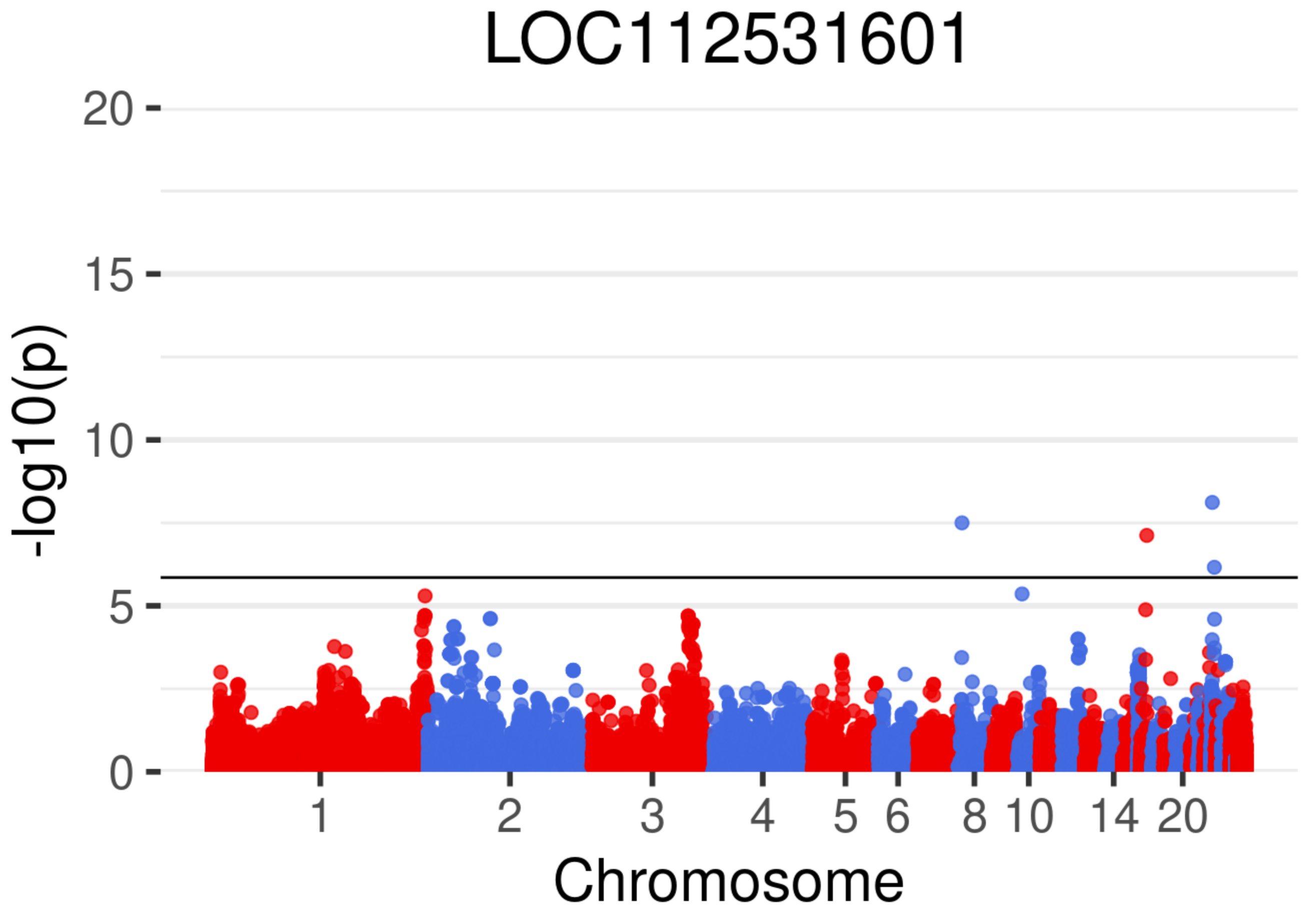
LOC112531601
-log10(p)
0
5
10
15
20
1
2
3
4
5
6
8
10
14
20
Chromosome

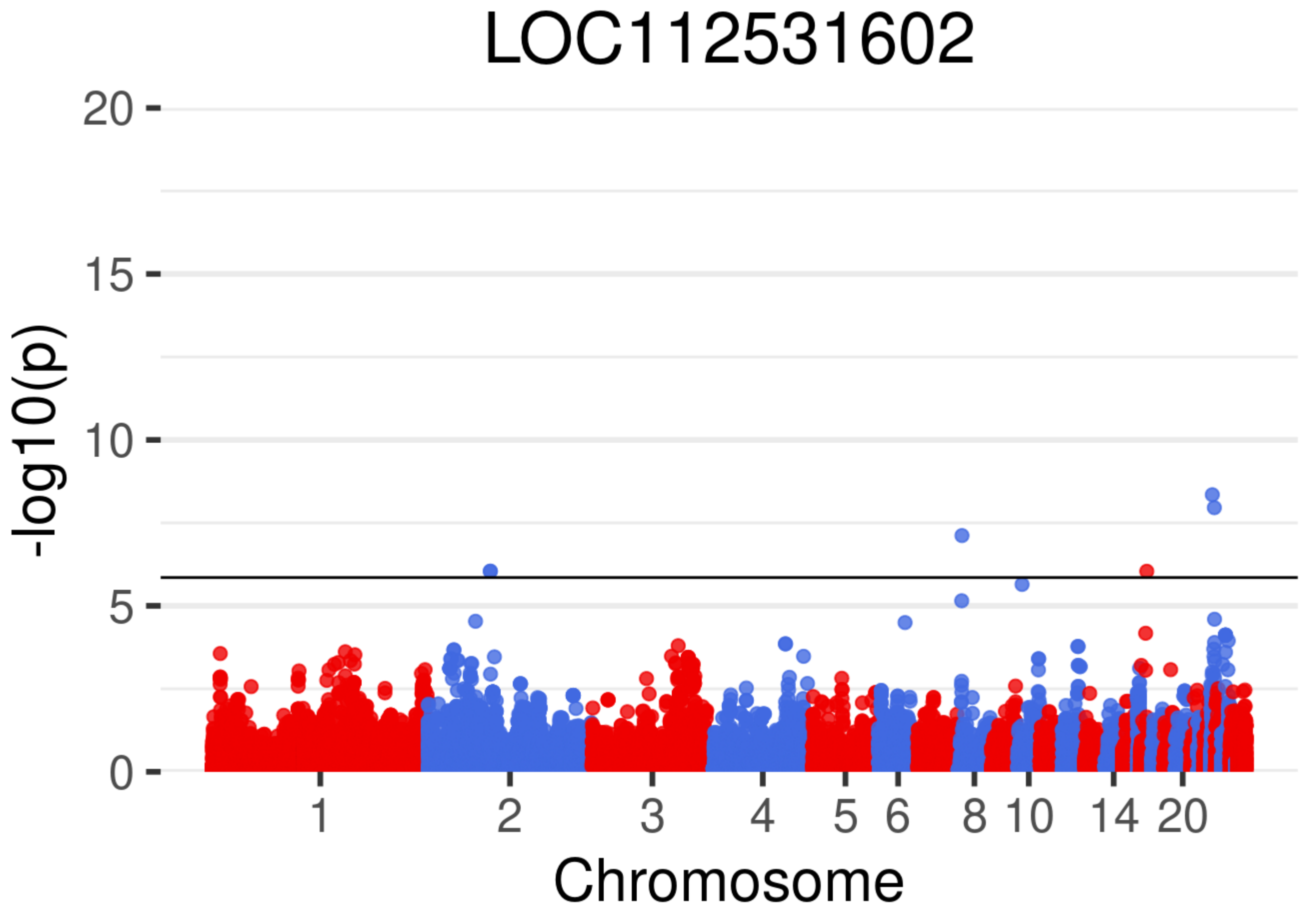
LOC112531602
-log10(p)
20
15
10
5
0
1
2
3
4
5
6
8
10
14
20
Chromosome

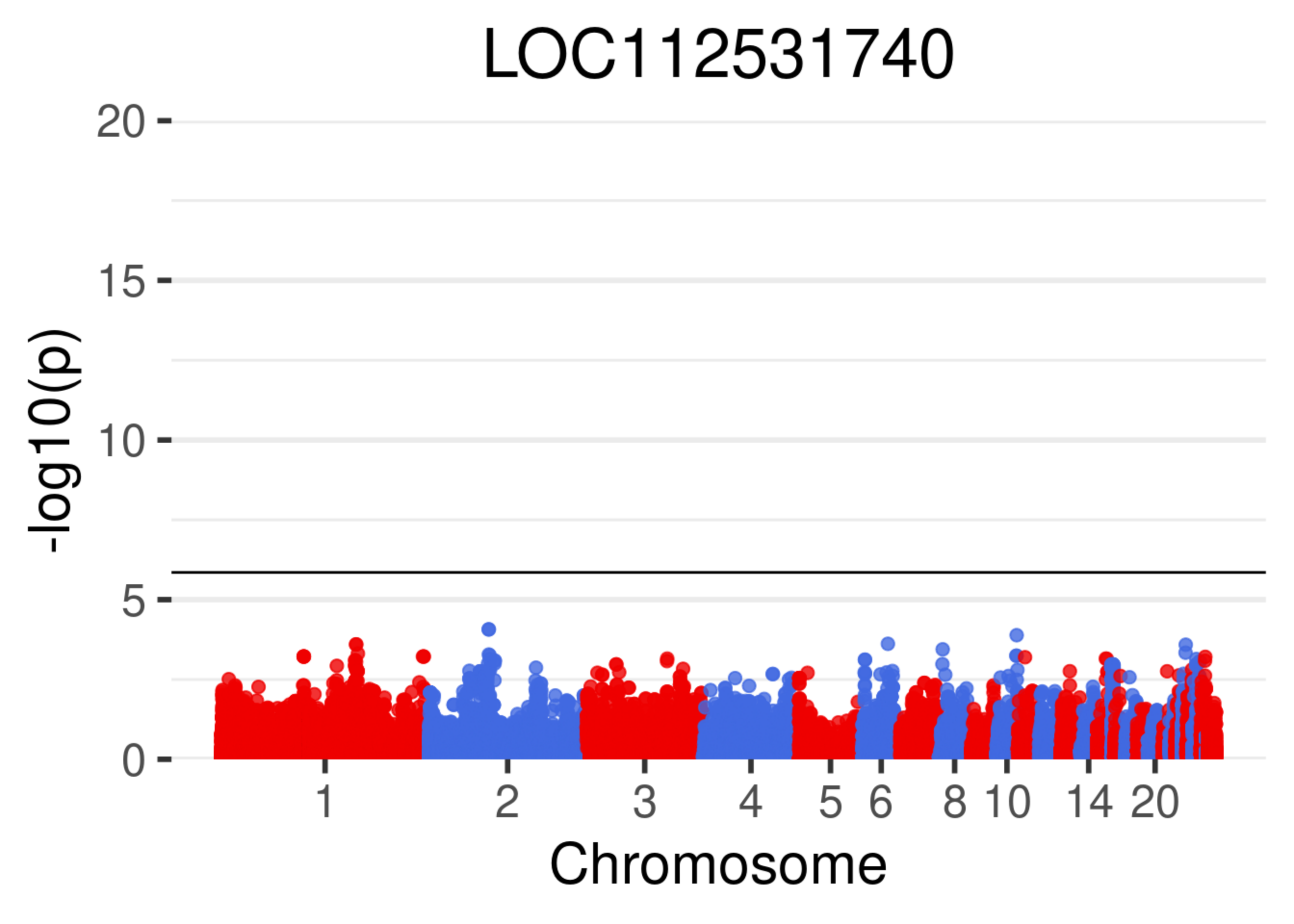
LOC112531740
20
15
10
5
0
-log10(p)
1
2
3
4
5
6
8
10
14
20
Chromosome

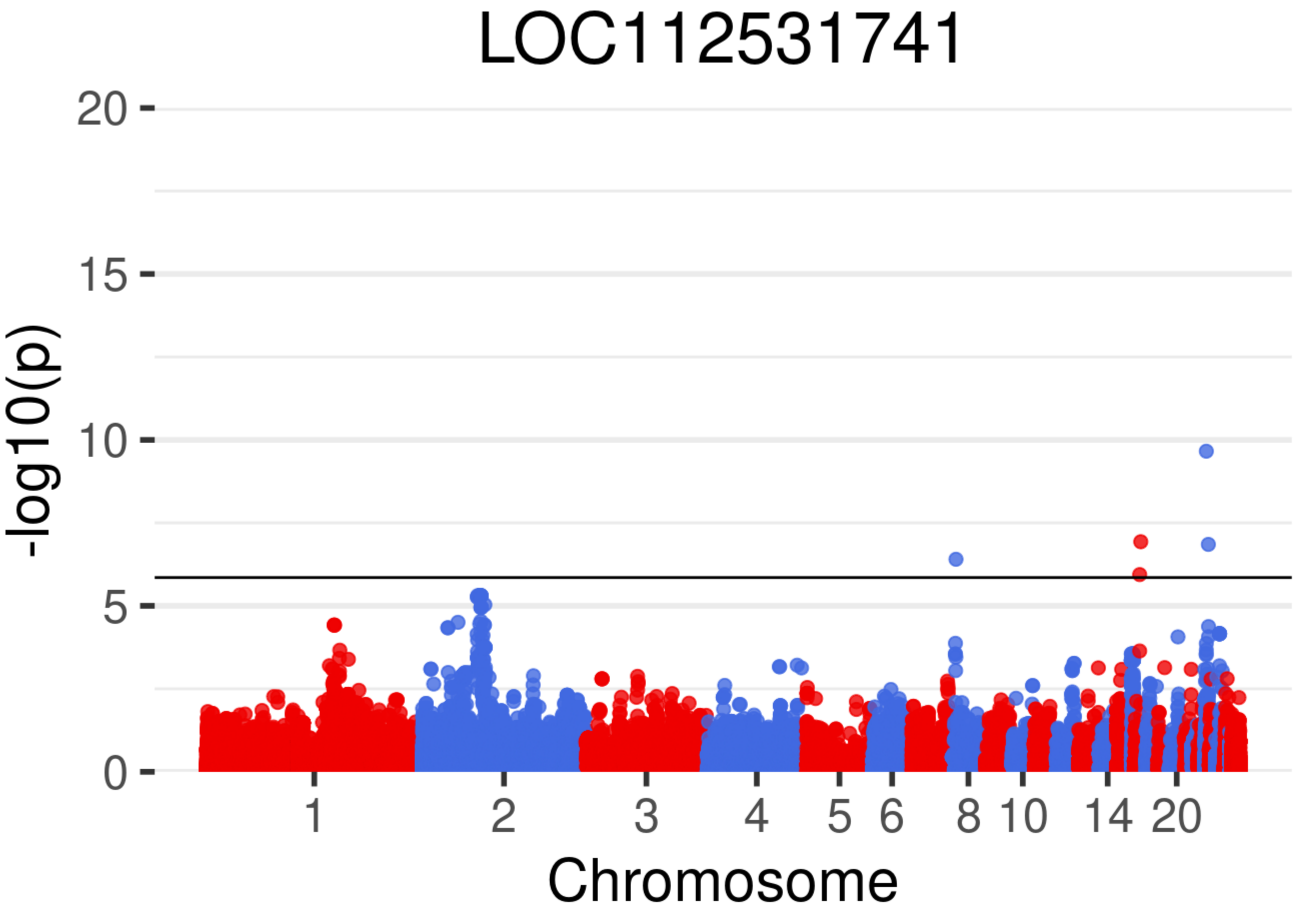
LOC112531741
20
15
10
5
0
-log10(p)
1
2
3
4
5
6
8
10
14
20
Chromosome

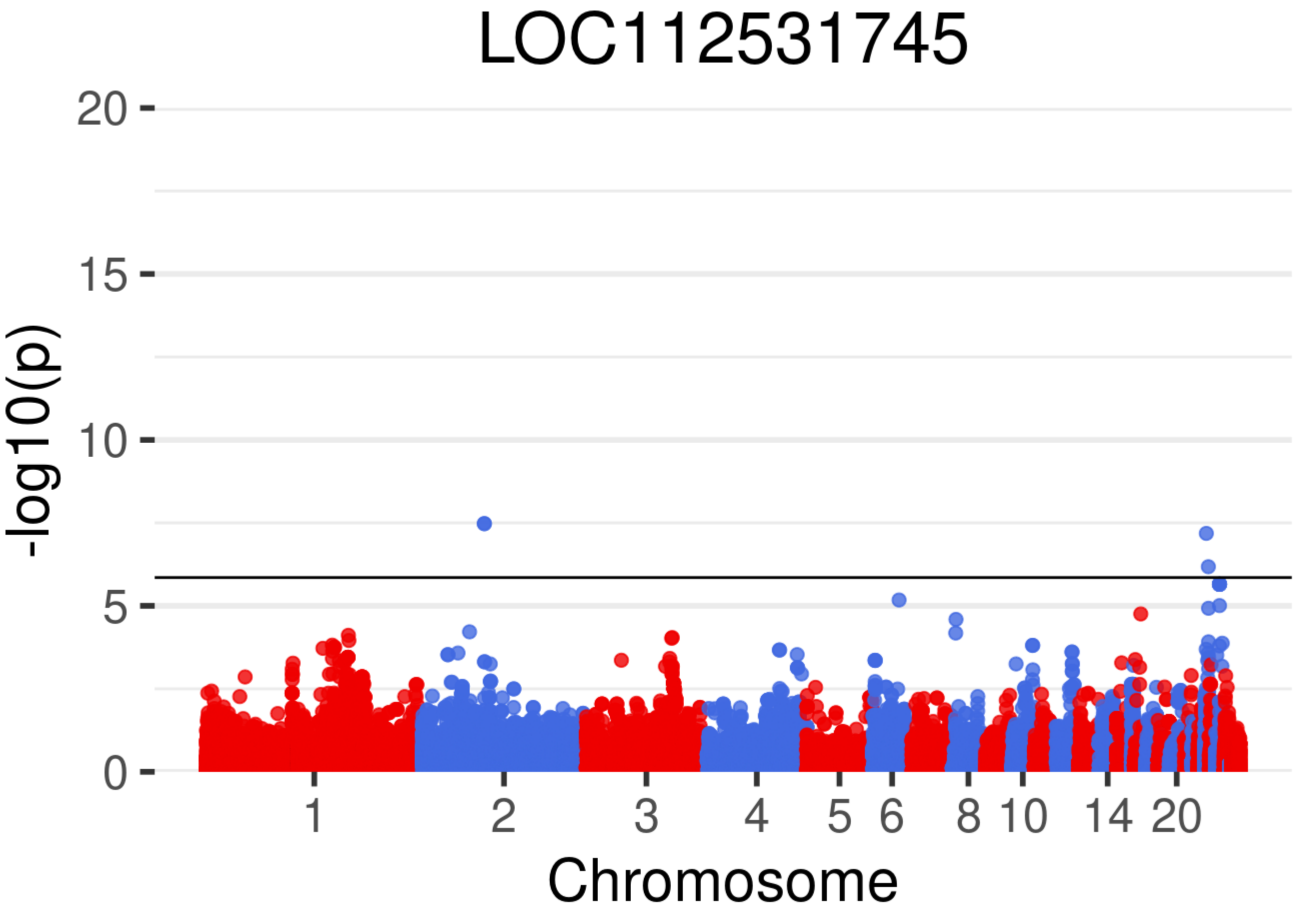

LOC112531745
-log10(p)
20
15
10
5
0
1
2
3
4
5
6
8
10
14
20
Chromosome

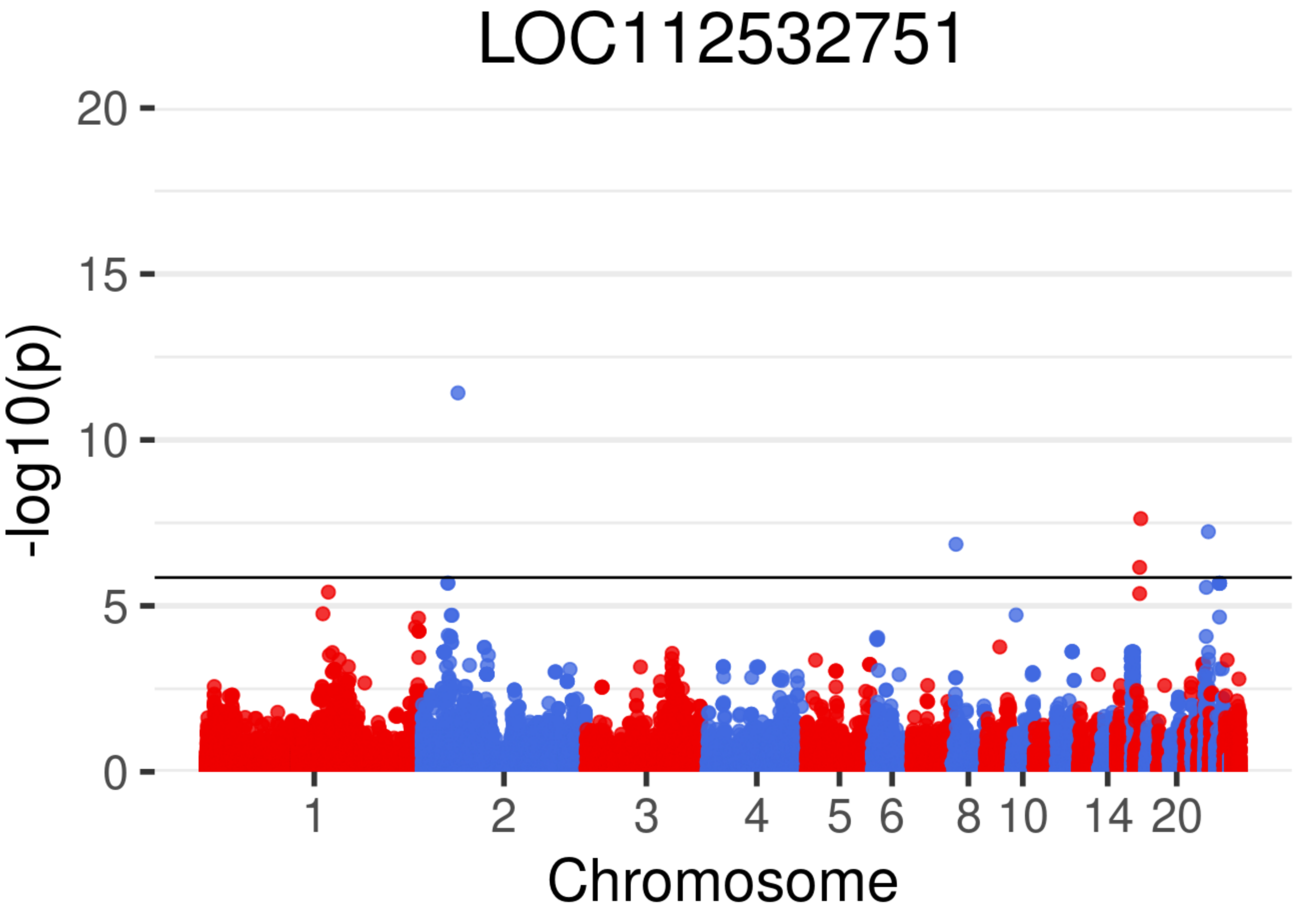
LOC112532751
20
15
10
5
0
-log10(p)
1
2
3
4
5
6
8
10
14
20
Chromosome

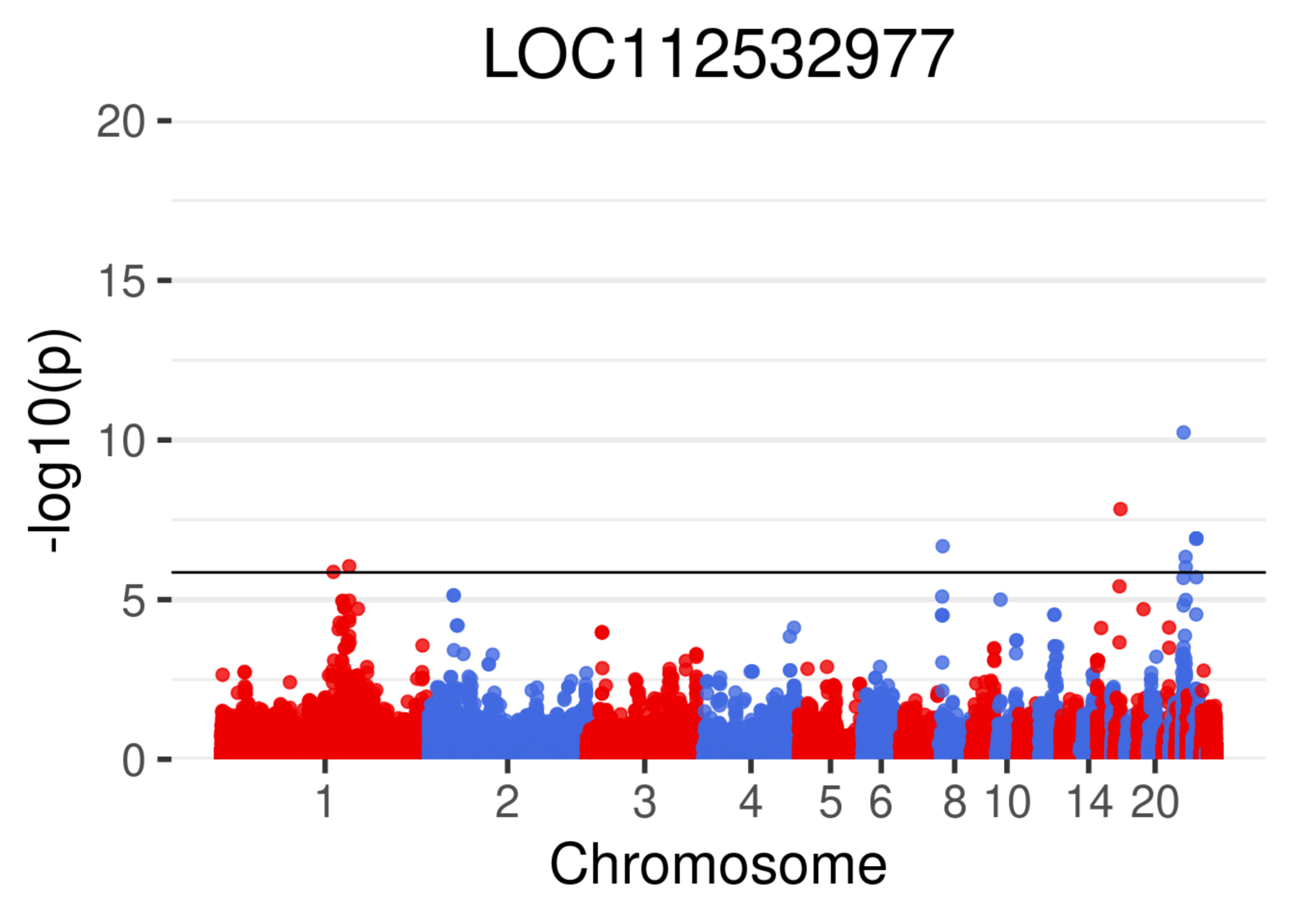
LOC112532977
-log10(p)
0
5
10
15
20
1
2
3
4
5
6
8
10
14
20
Chromosome

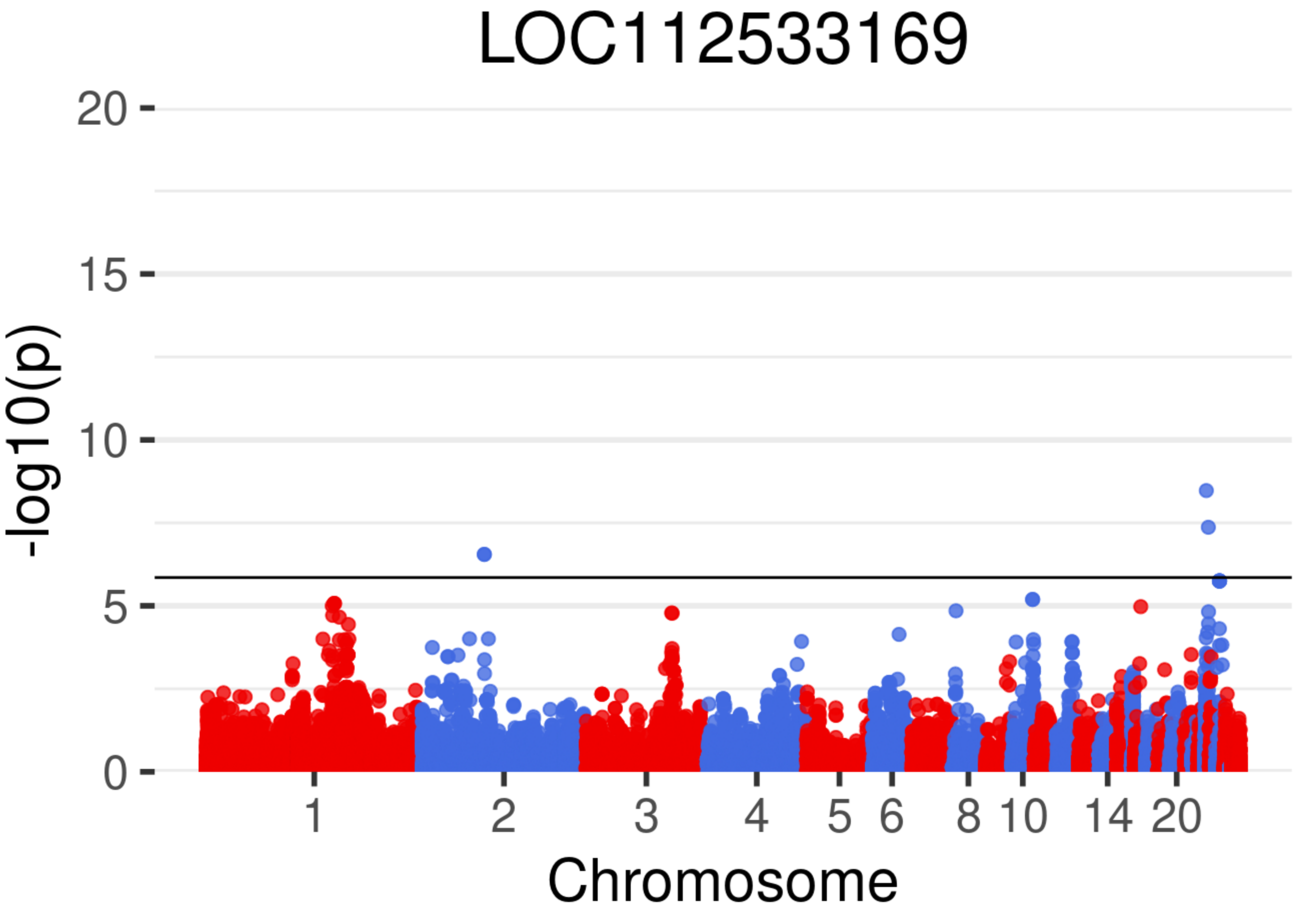
LOC112533169
-log10(p)
20
15
10
5
0
1
2
3
4
5
6
8
10
14
20
Chromosome

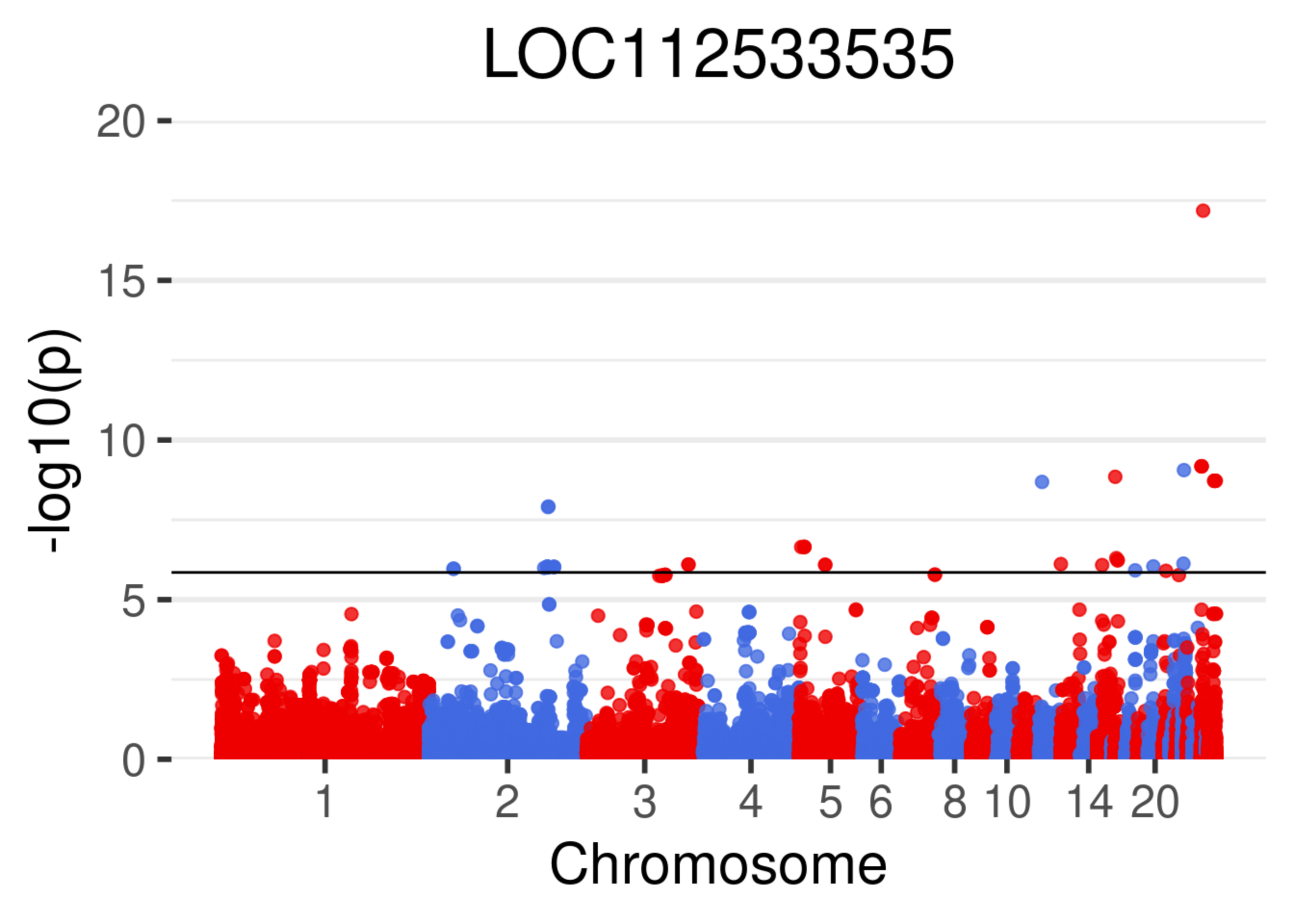

LOC112533535
-log10(p)
20
15
10
5
0
1
2
3
4
5
6
8
10
14
20
Chromosome

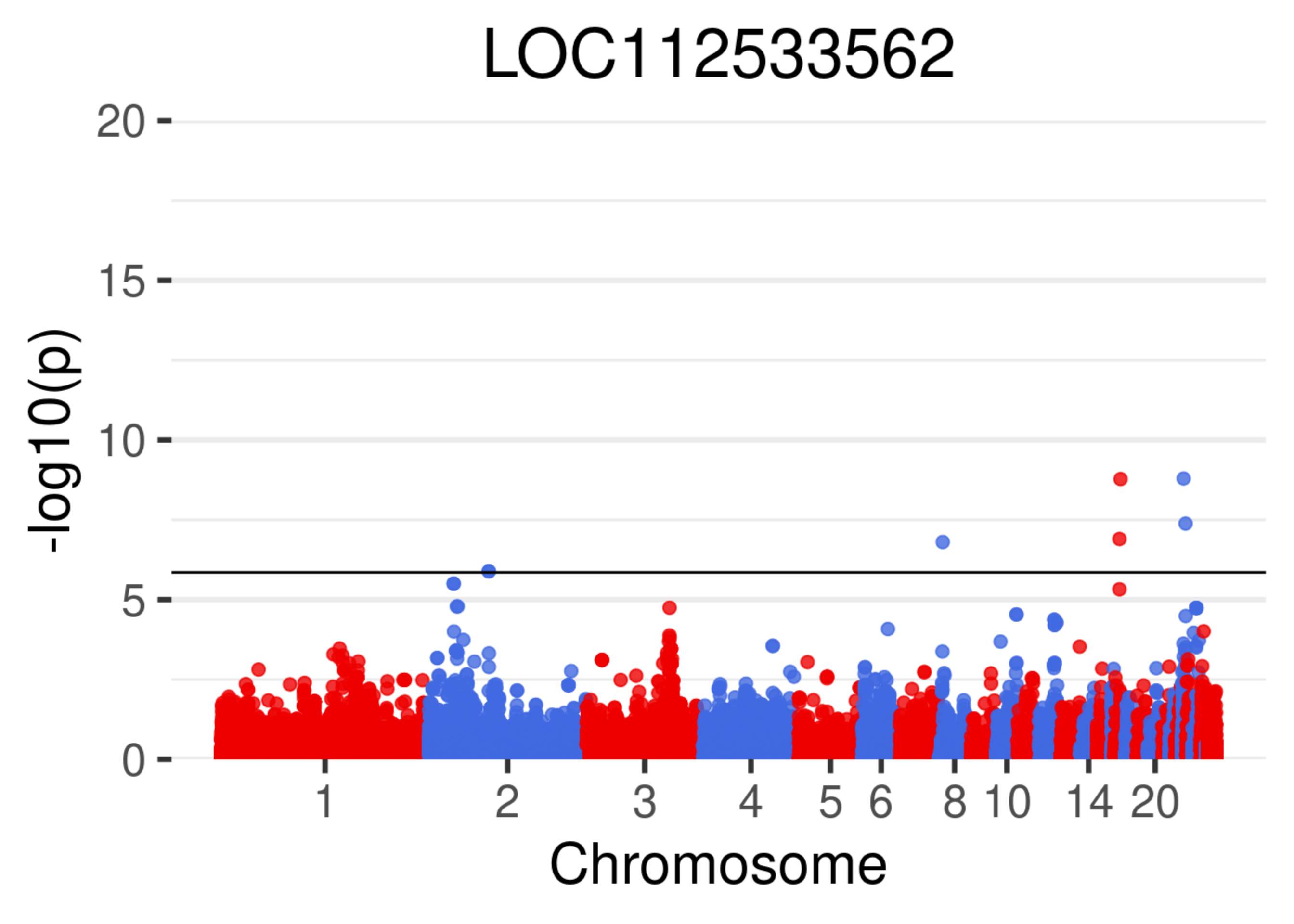

LOC112533562
-log10(p)
20
15
10
5
0
Chromosome
1
2
3
4
5
6
8
10
14
20

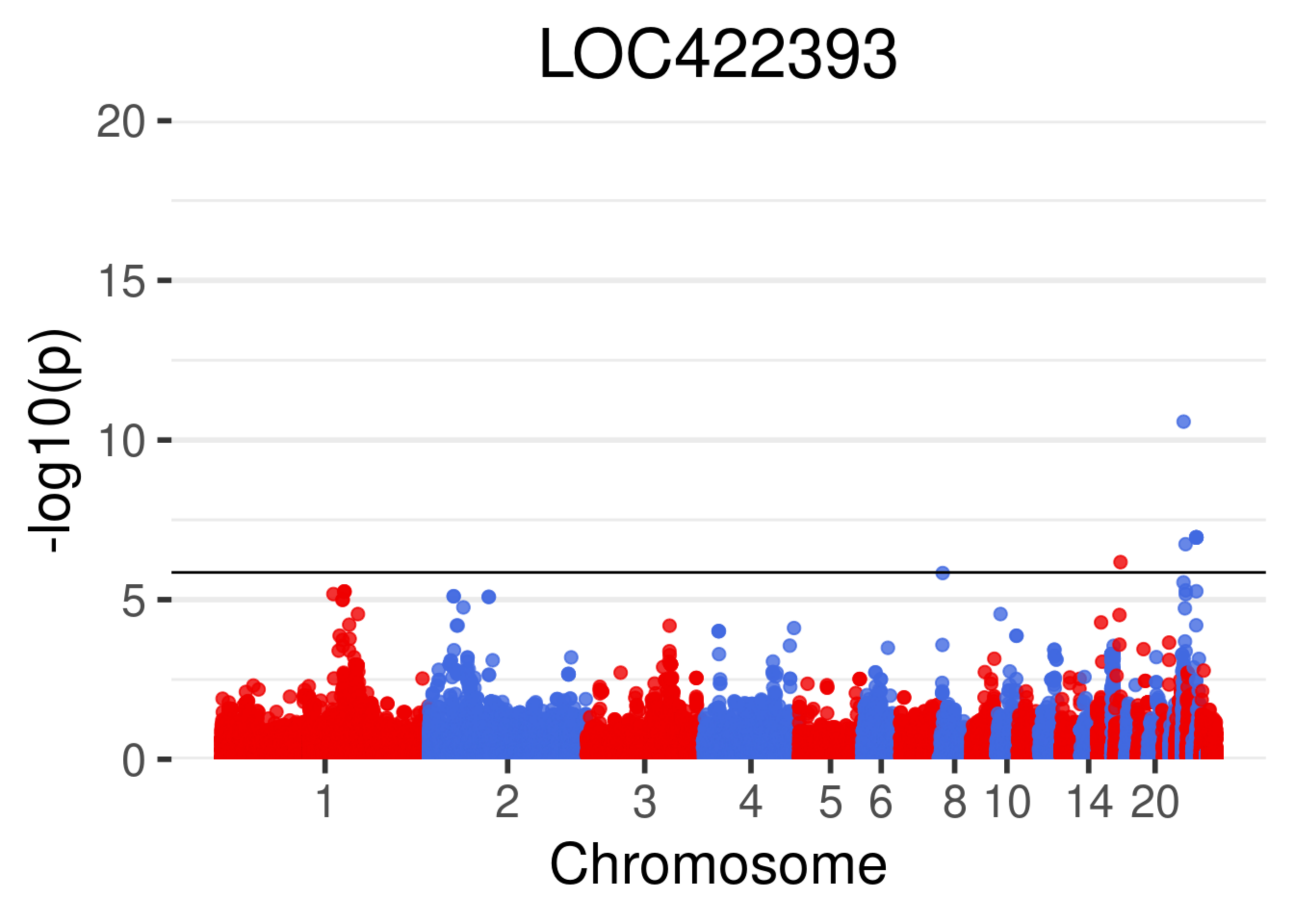

LOC422393
-log10(p)
20
15
10
5
0
1
2
3
4
5
6
8
10
14
20
Chromosome

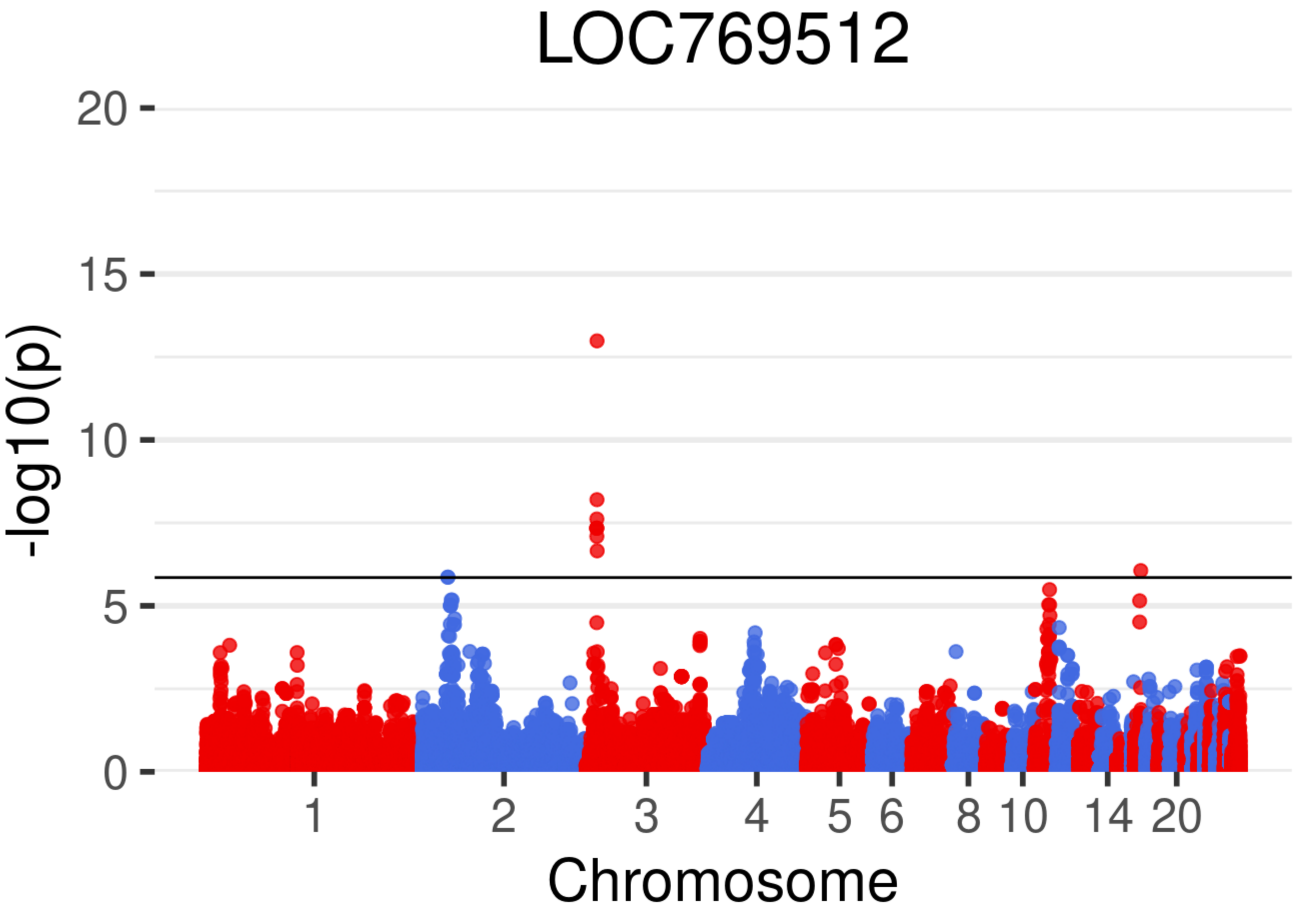

LOC769512
20
15
10
5
0
-log10(p)
1
2
3
4
5
6
8
10
14
20
Chromosome

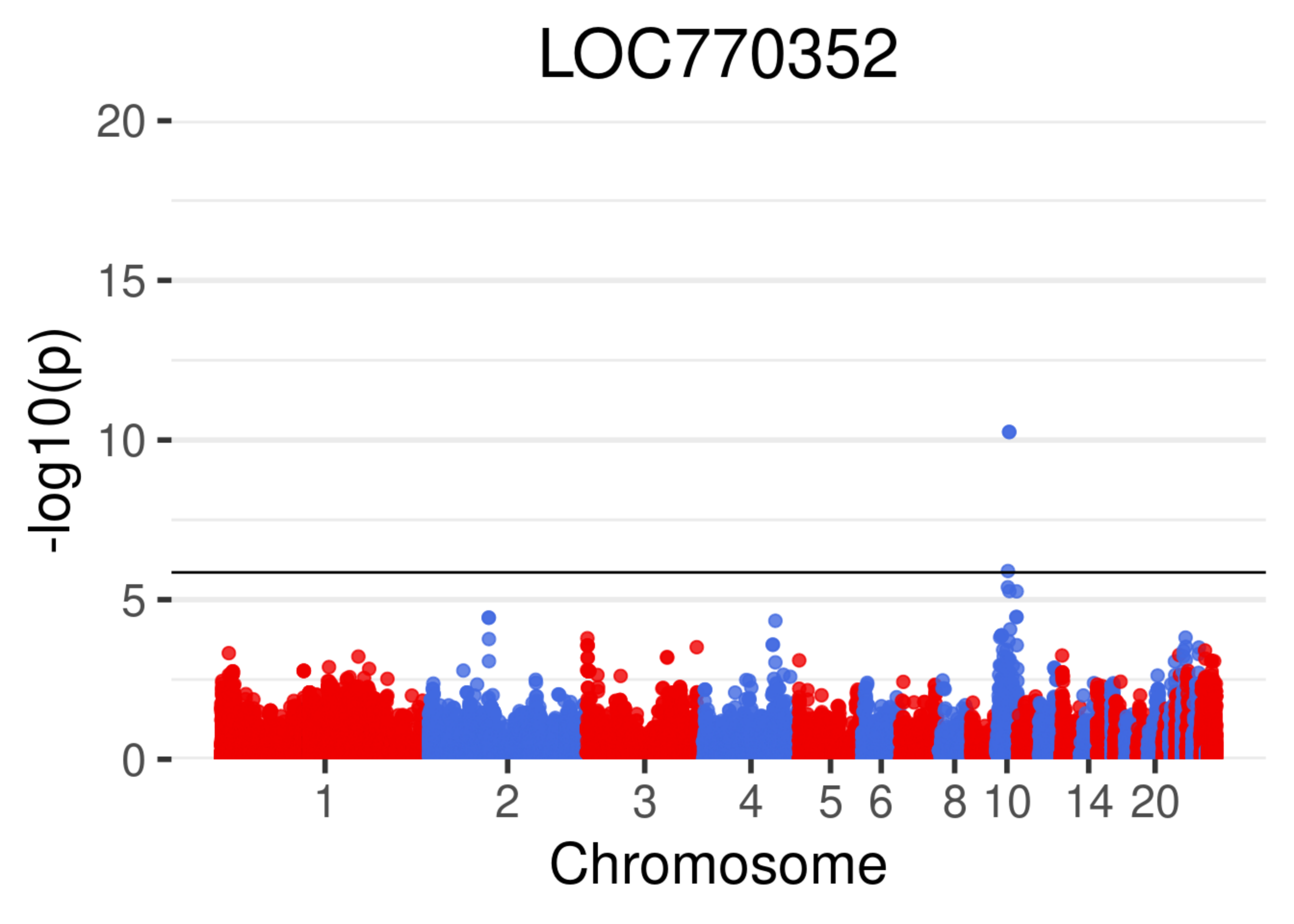
LOC770352
-log10(p)
20
15
10
5
0
1
2
3
4
5
6
8
10
14
20
Chromosome

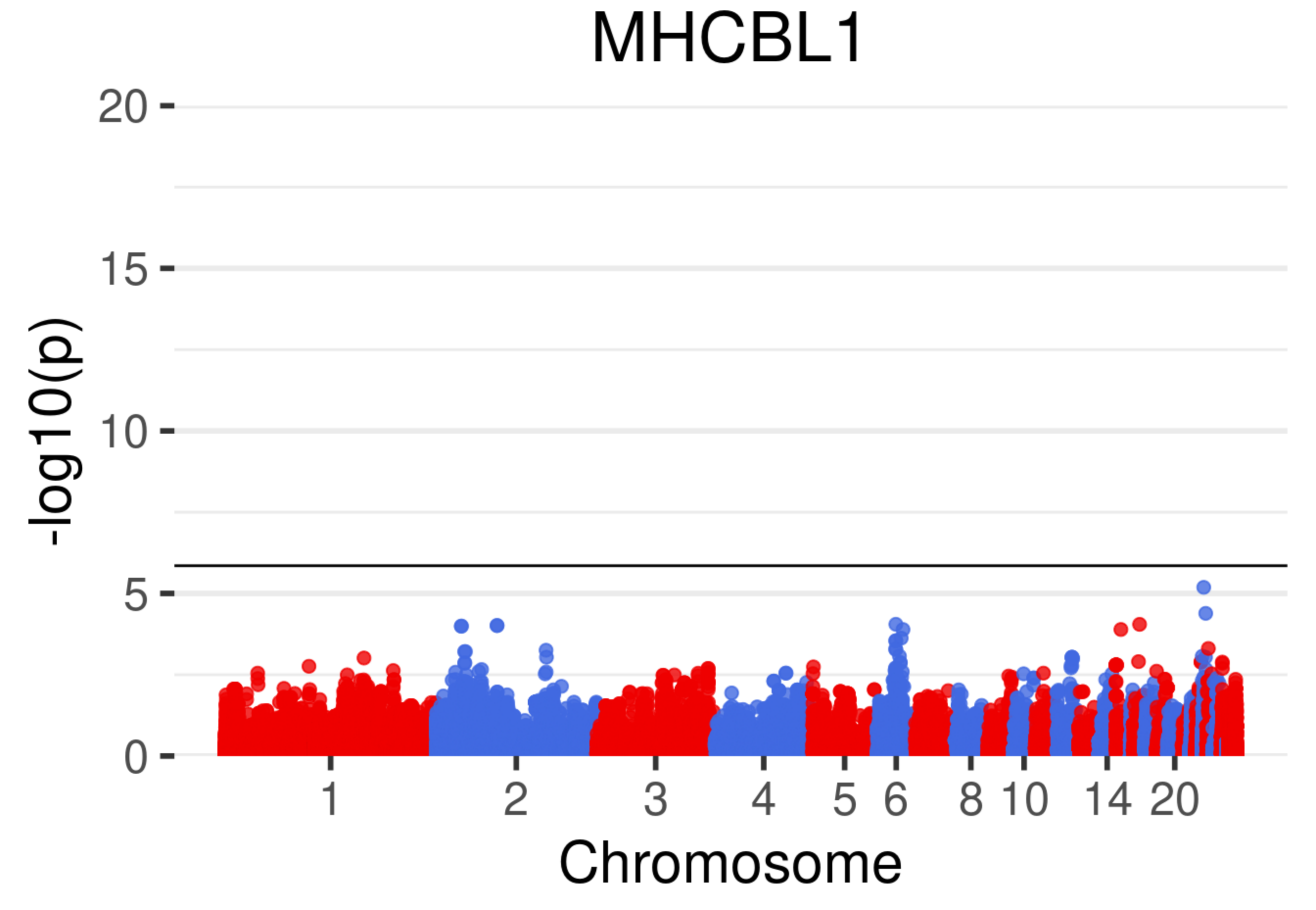

MHCBL1
-log10(p)
20
15
10
5
0
1
2
3
4
5
6
8
10
14
20
Chromosome

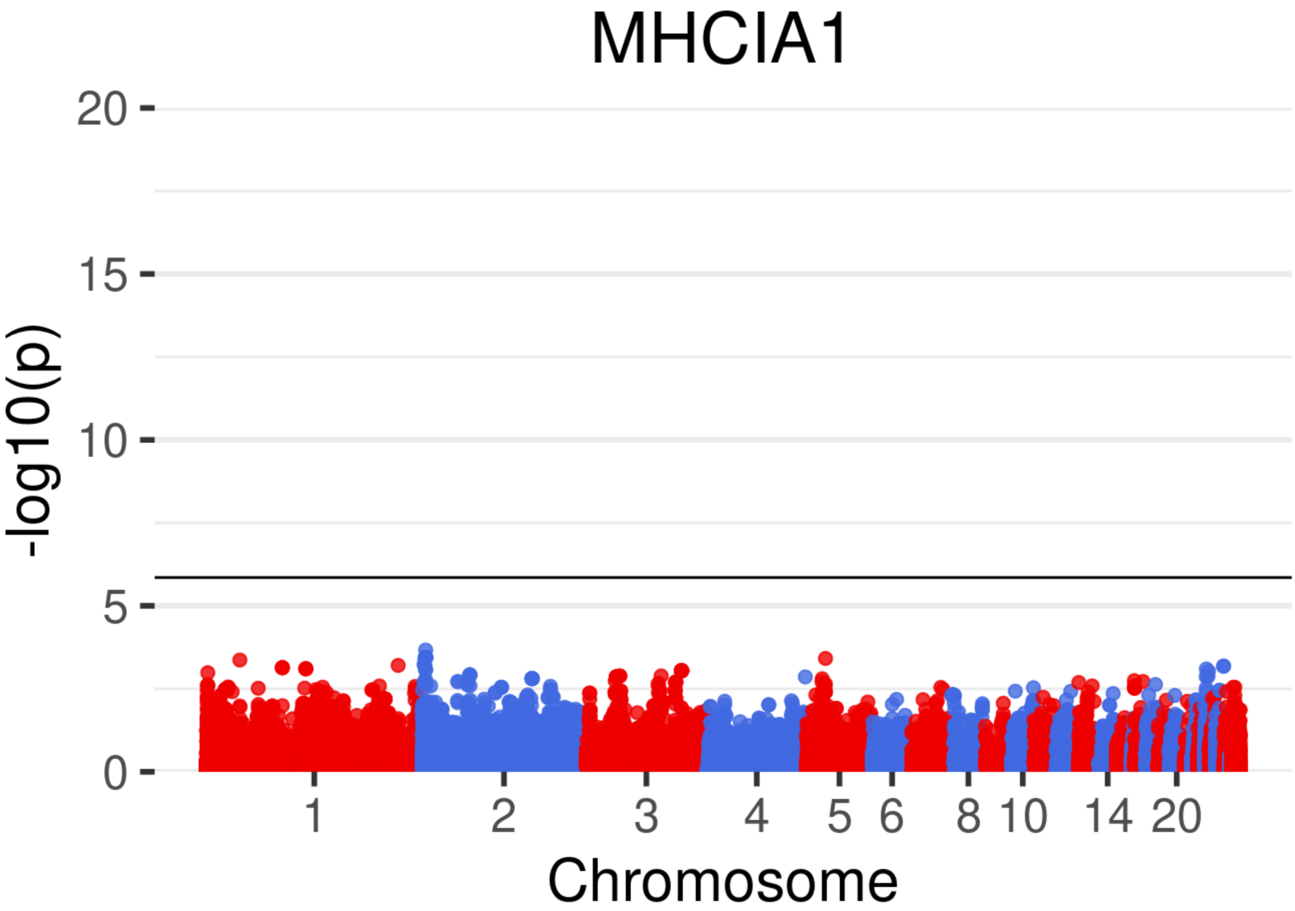
MHCIA1
20
15
10
5
0
-log10(p)
1
2
3
4
5
6
8
10
14
20
Chromosome

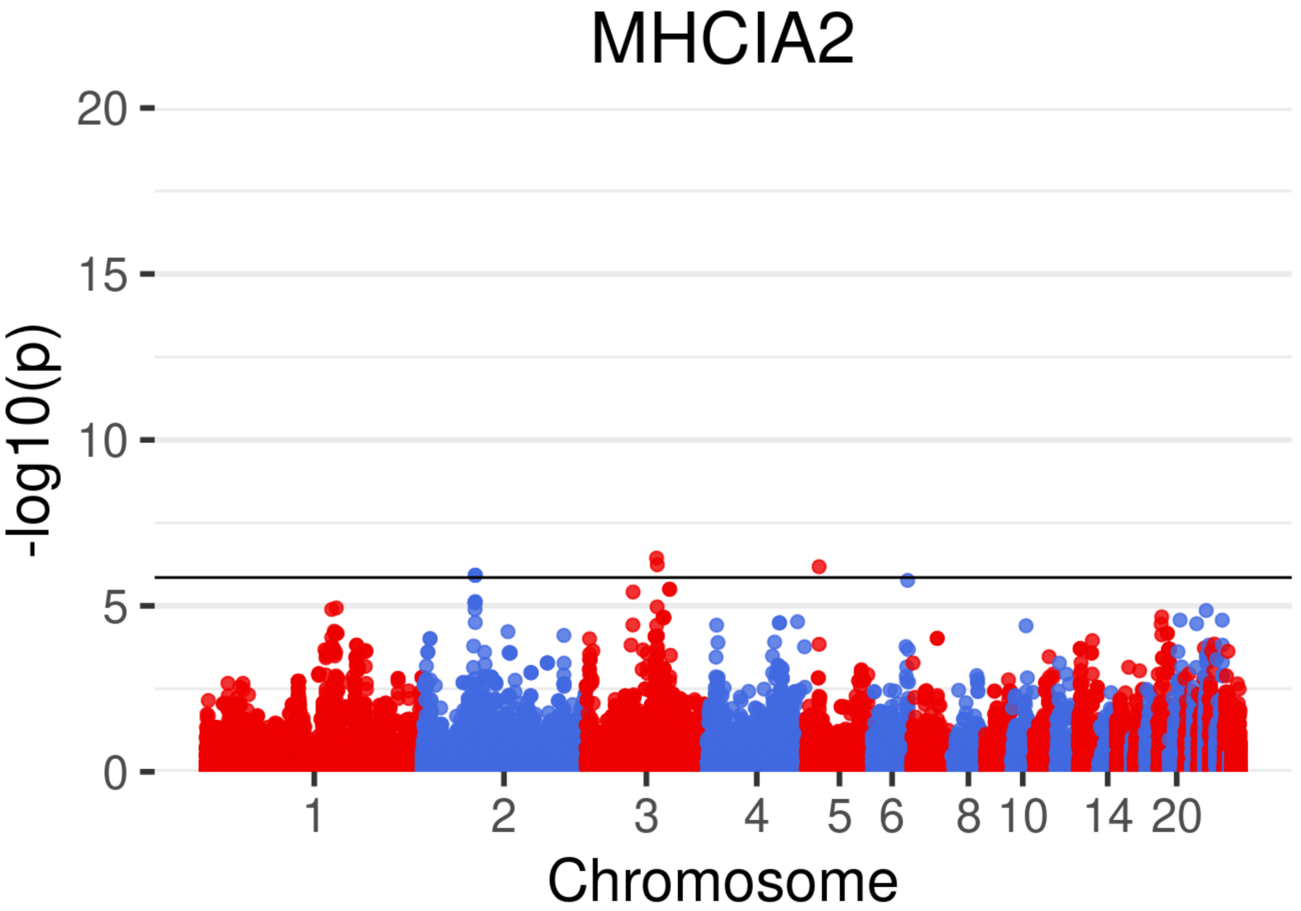
MHCIA2
20
15
10
5
0
-log10(p)
1
2
3
4
5
6
8
10
14
20
Chromosome

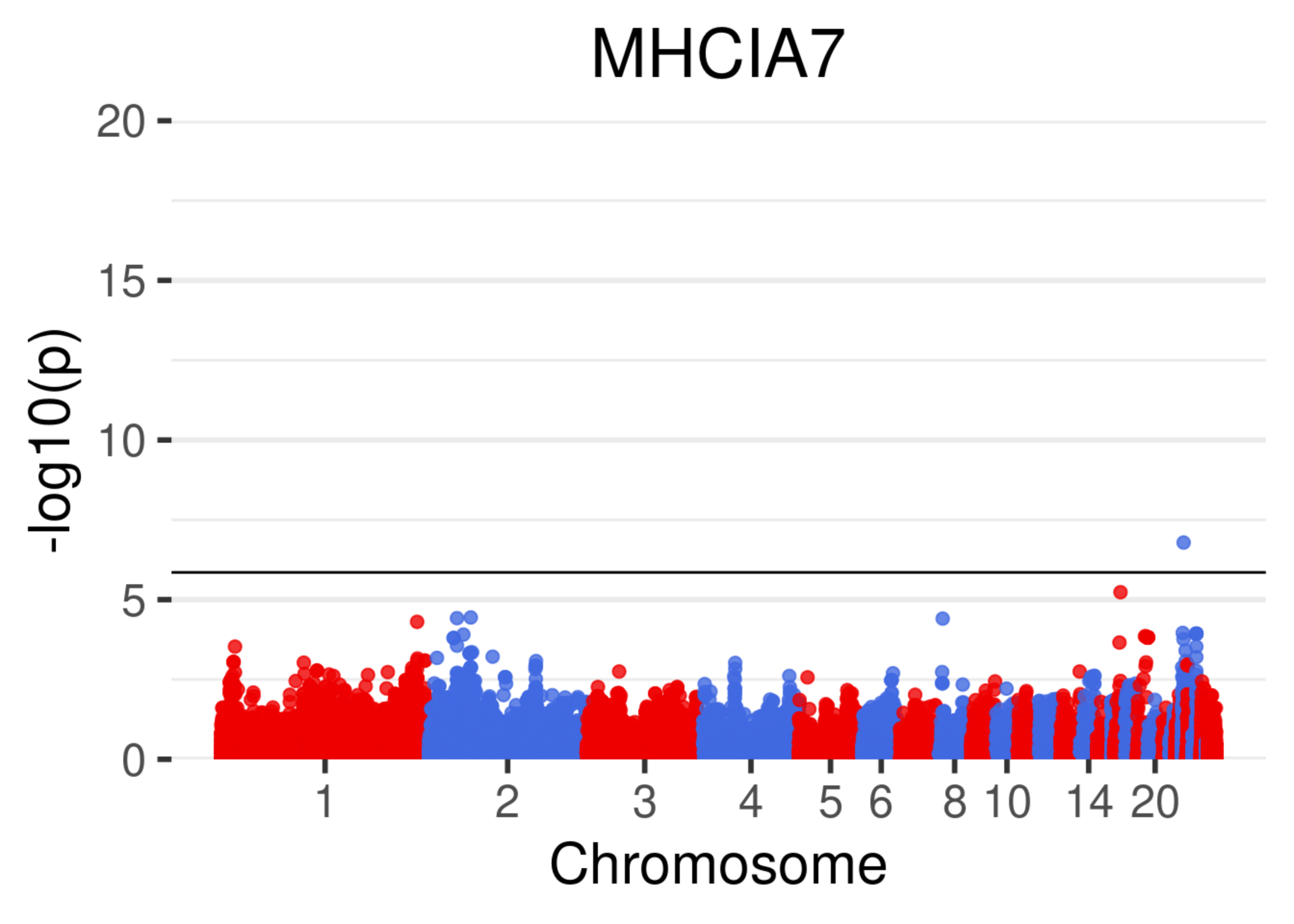

MHCIA7
20
15
10
5
0
-log10(p)
1
2
3
4
5
6
8
10
14
20
Chromosome

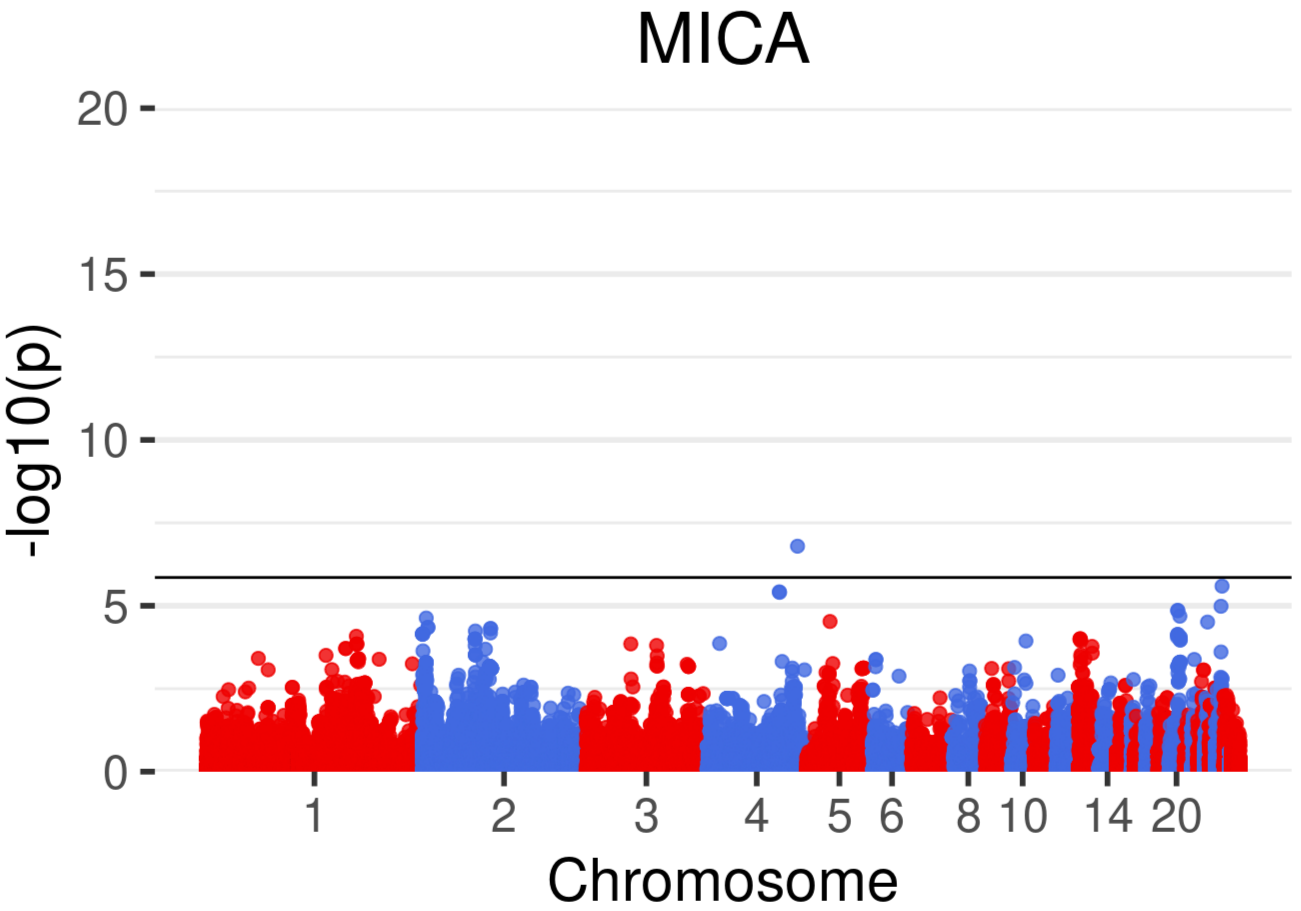
MICA
-log10(p)
20
15
10
5
0
1
2
3
4
5
6
8
10
14
20
Chromosome

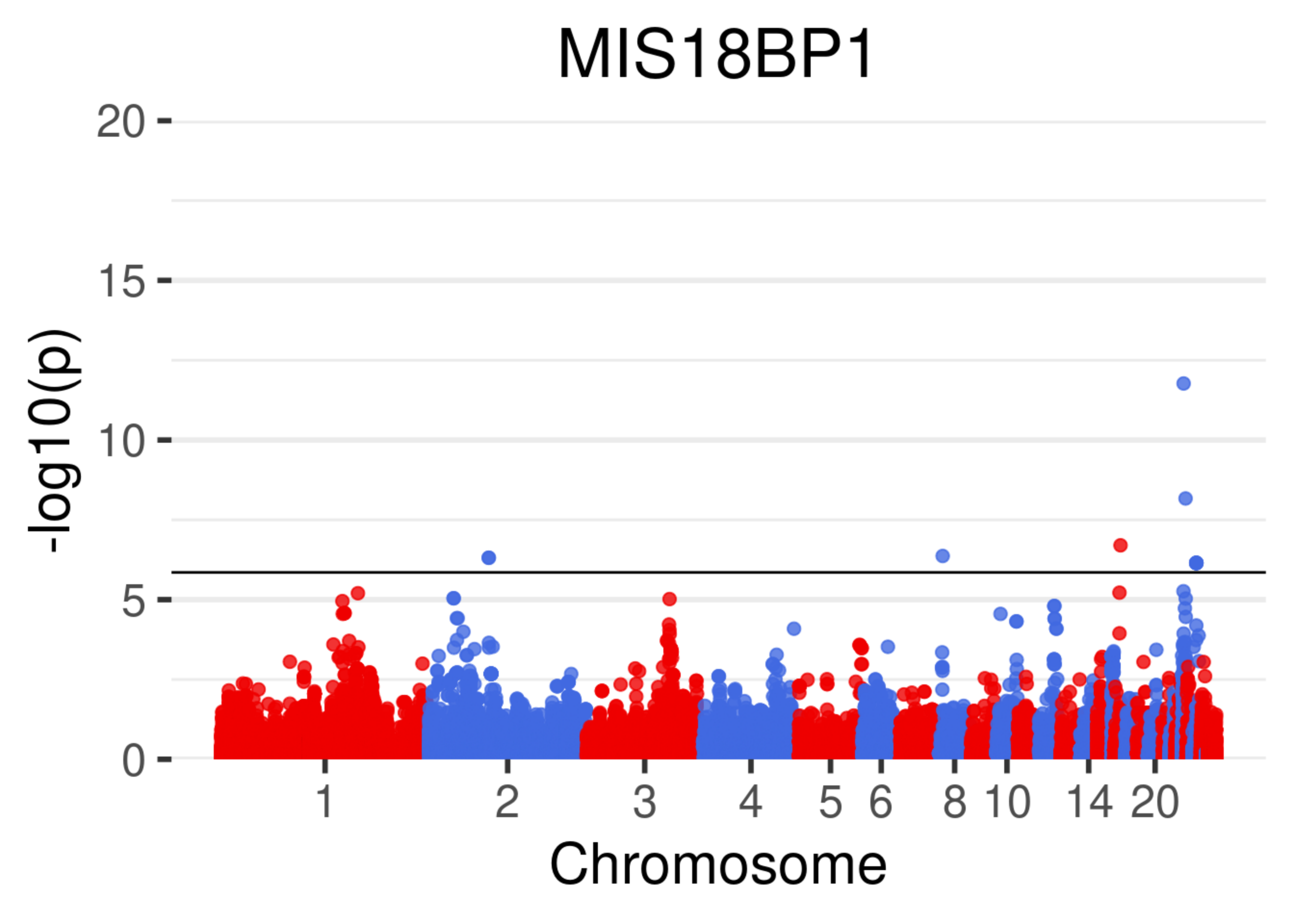

MIS18BP1
20
15
10
5
0
-log10(p)
1
2
3
4
5
6
8
10
14
20
Chromosome

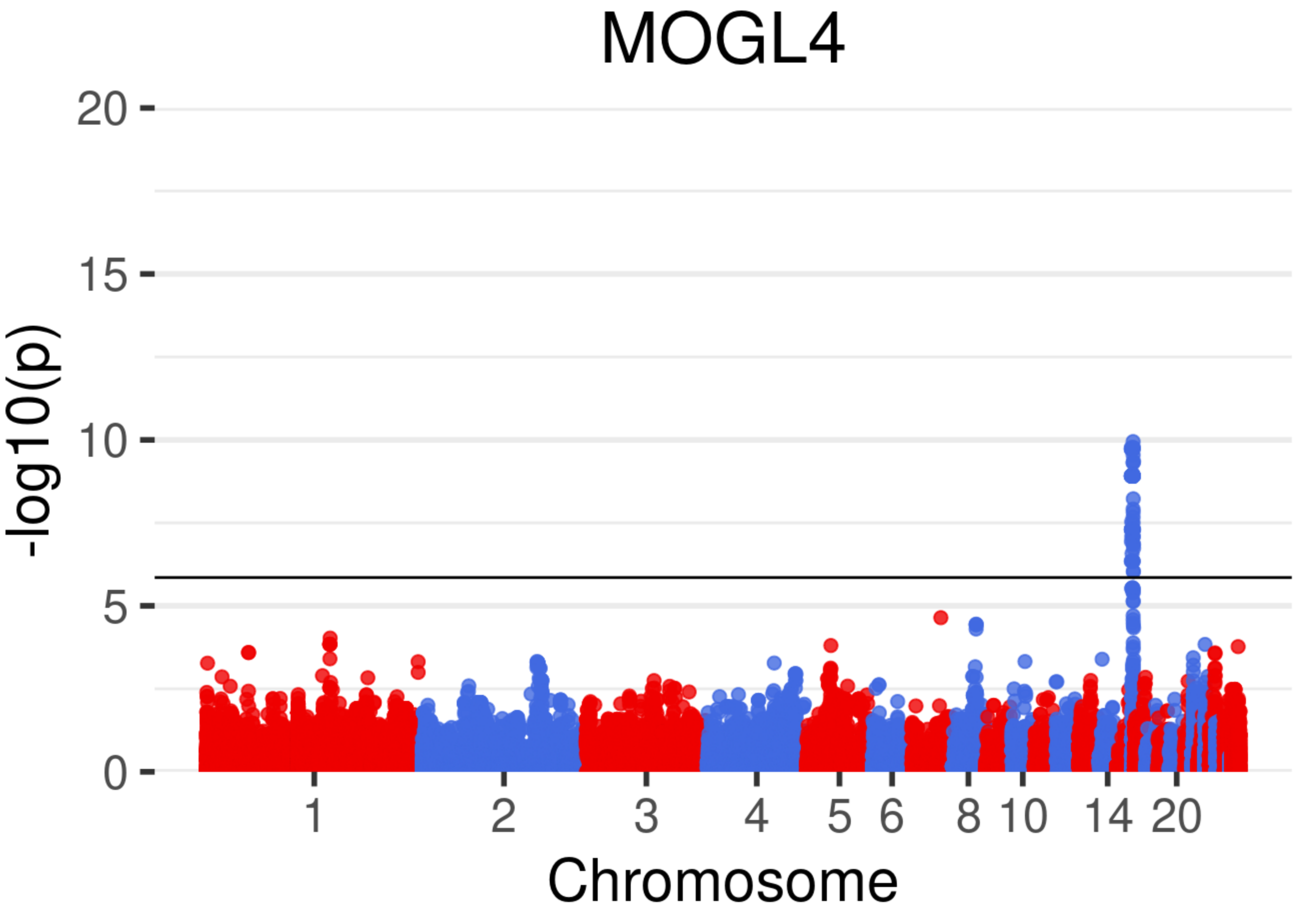
MOGL4
20
15
10
5
0
-log10(p)
1
2
3
4
5
6
8
10
14
20
Chromosome

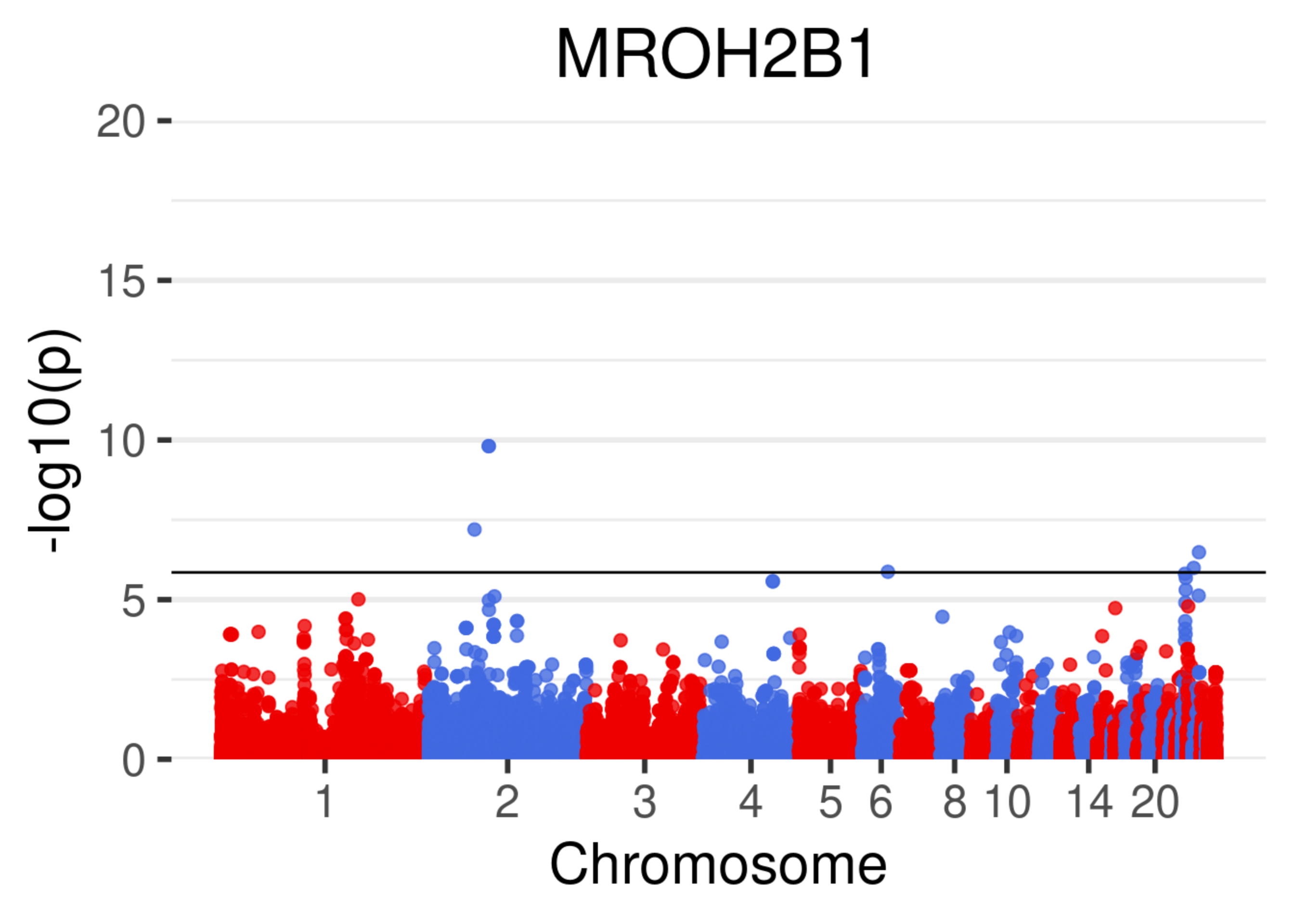
MROH2B1
-log10(p)
20
15
10
5
0
1
2
3
4
5
6
8
10
14
20
Chromosome

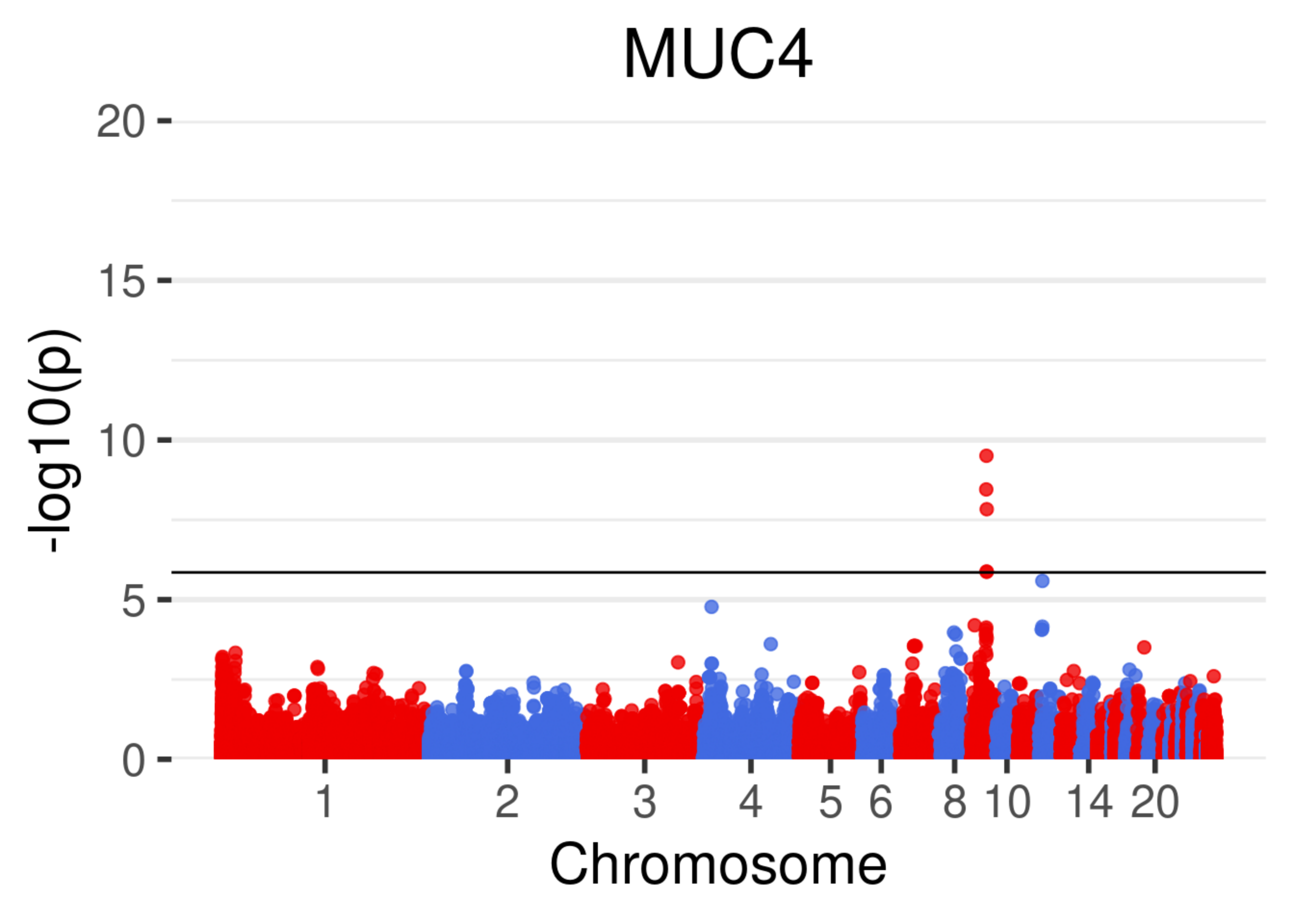
MUC4
20
15
10
5
0
-log10(p)
1
2
3
4
5
6
8
10
14
20
Chromosome

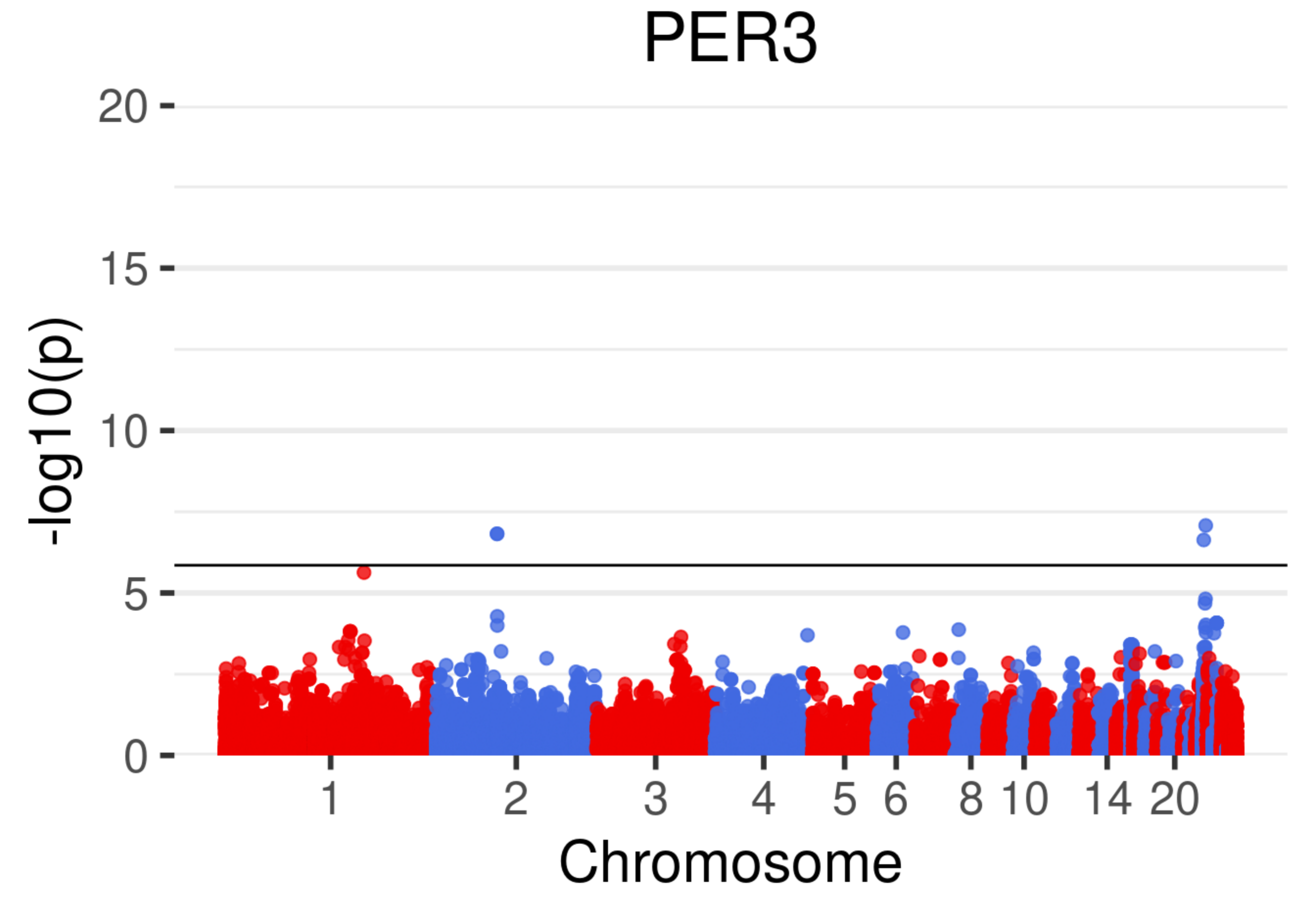
PER3
-log10(p)
20
15
10
5
0
1
2
3
4
5
6
8
10
14
20
Chromosome

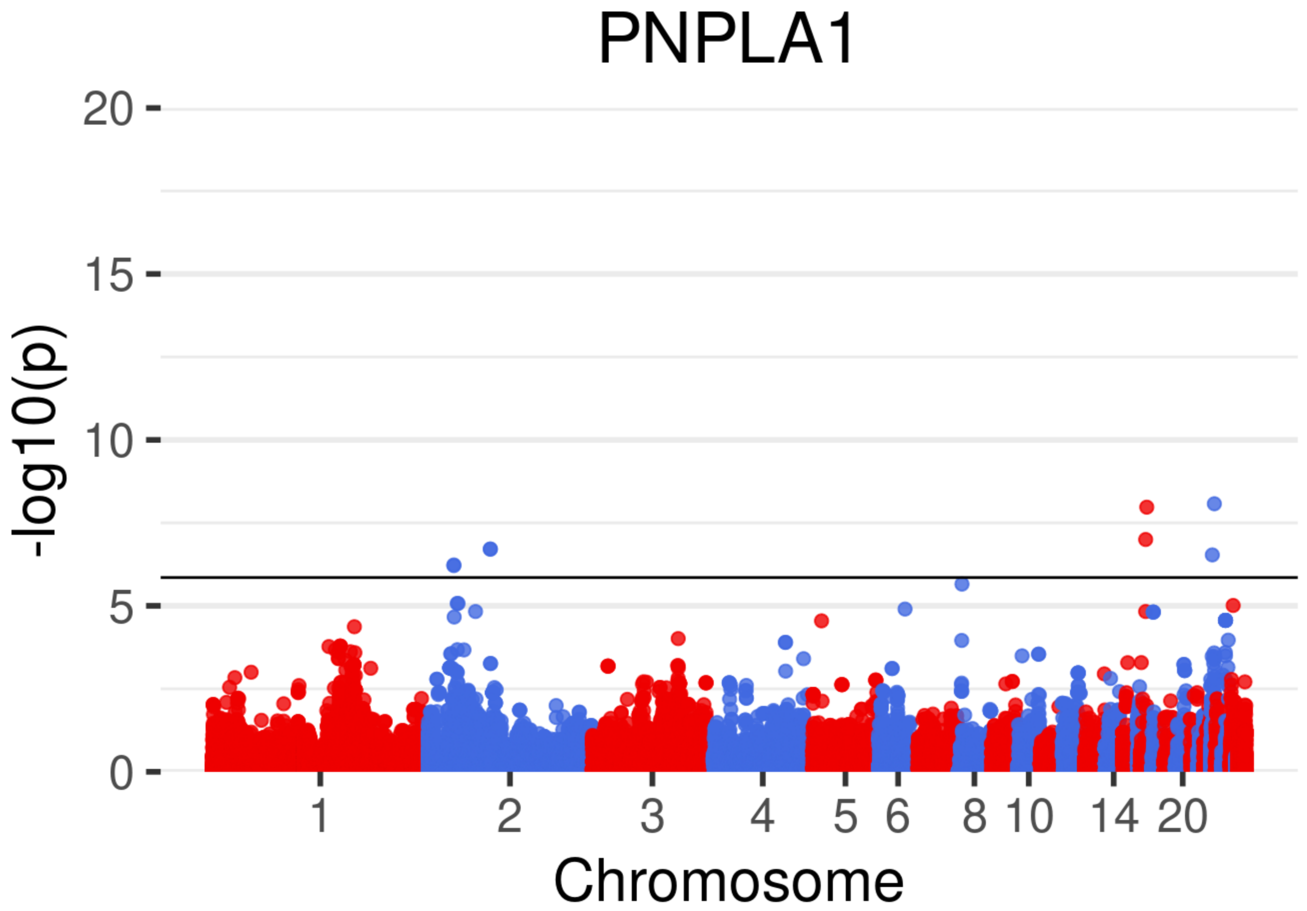
PNPLA1
20
15
10
5
0
-log10(p)
1
2
3
4
5
6
8
10
14
20
Chromosome

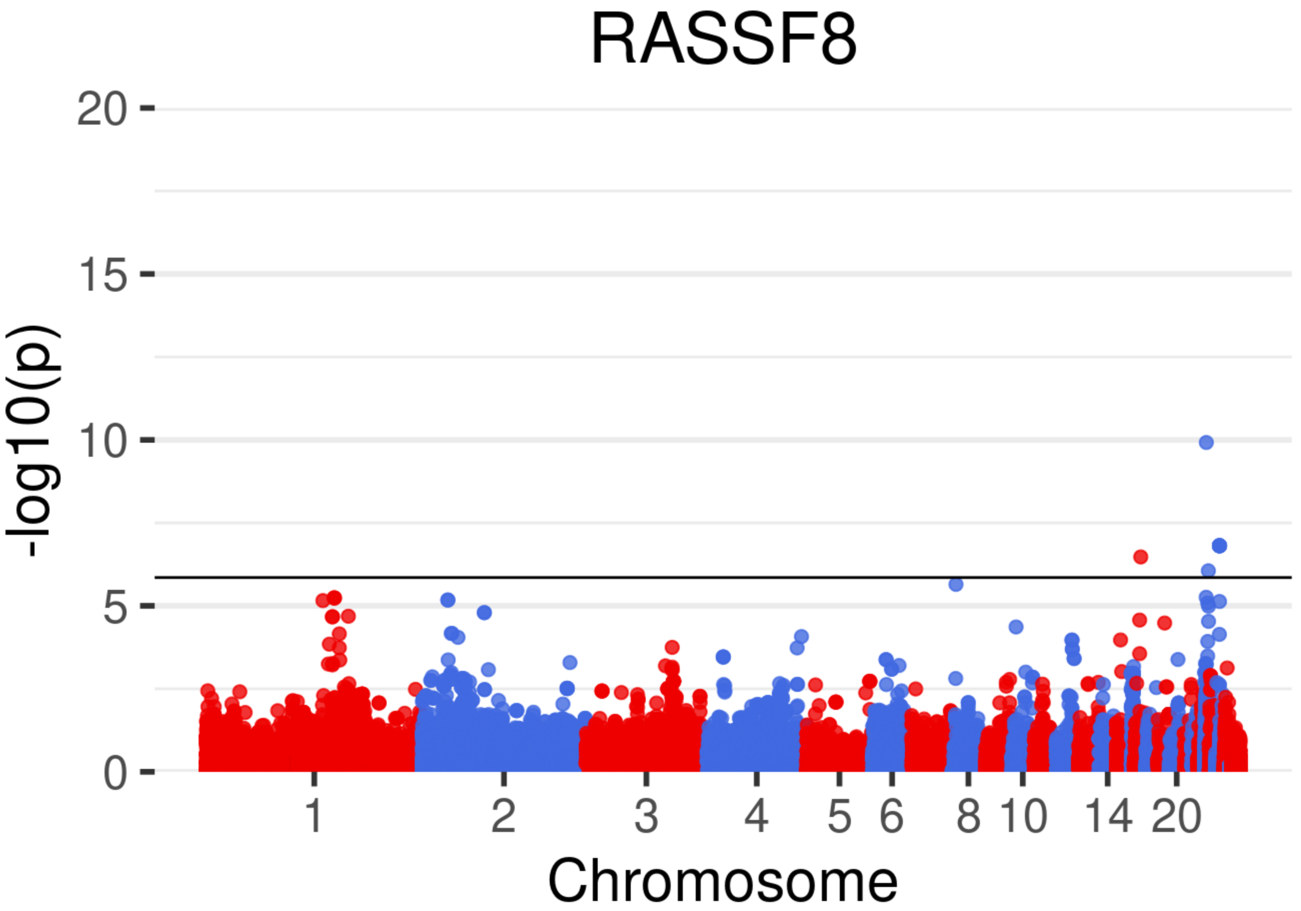

RASSF8
-log10(p)
20
15
10
5
0
1
2
3
4
5
6
8
10
14
20
Chromosome

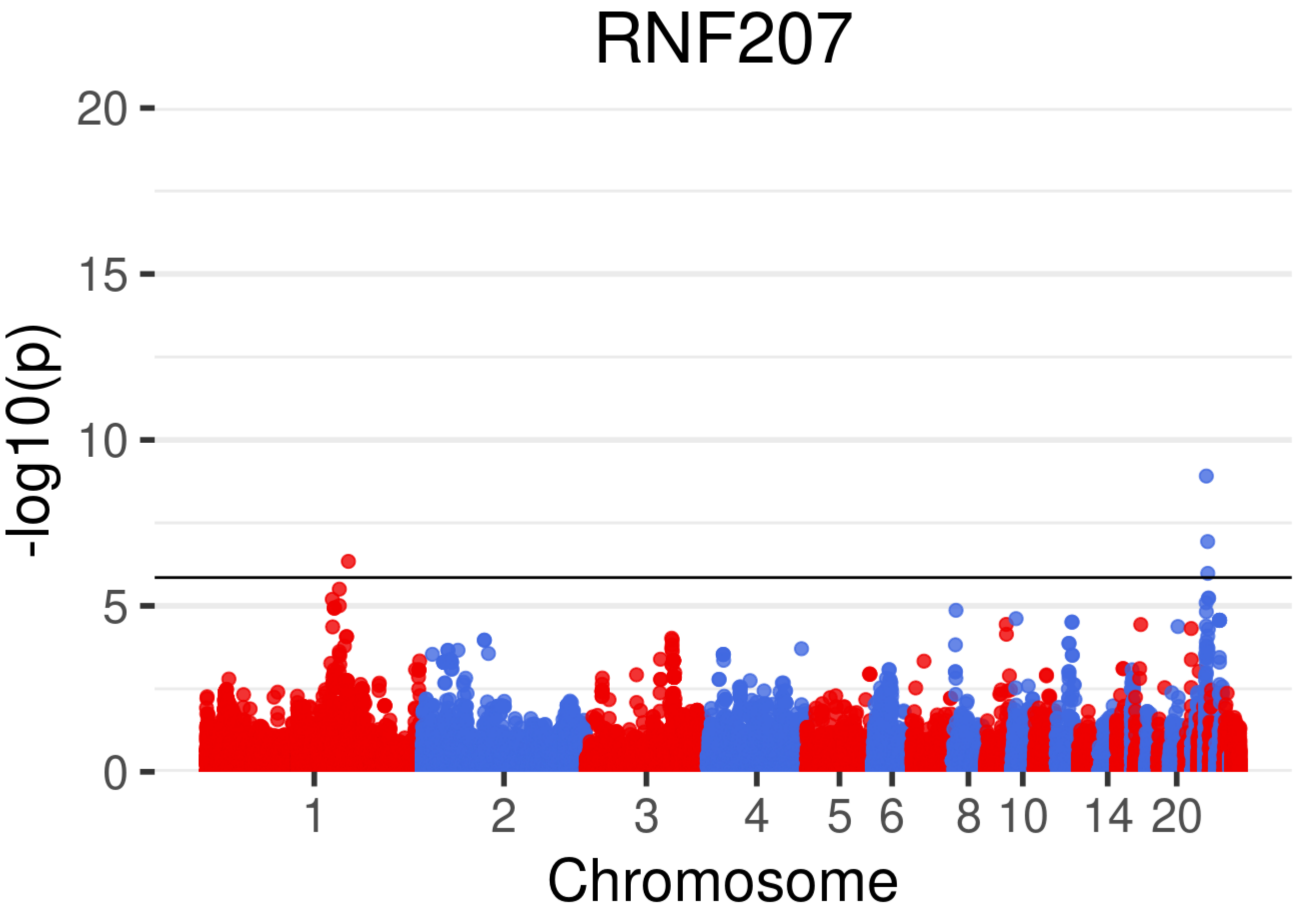
RNF207
-log10(p)
20
15
10
5
0
1
2
3
4
5
6
8
10
14
20
Chromosome

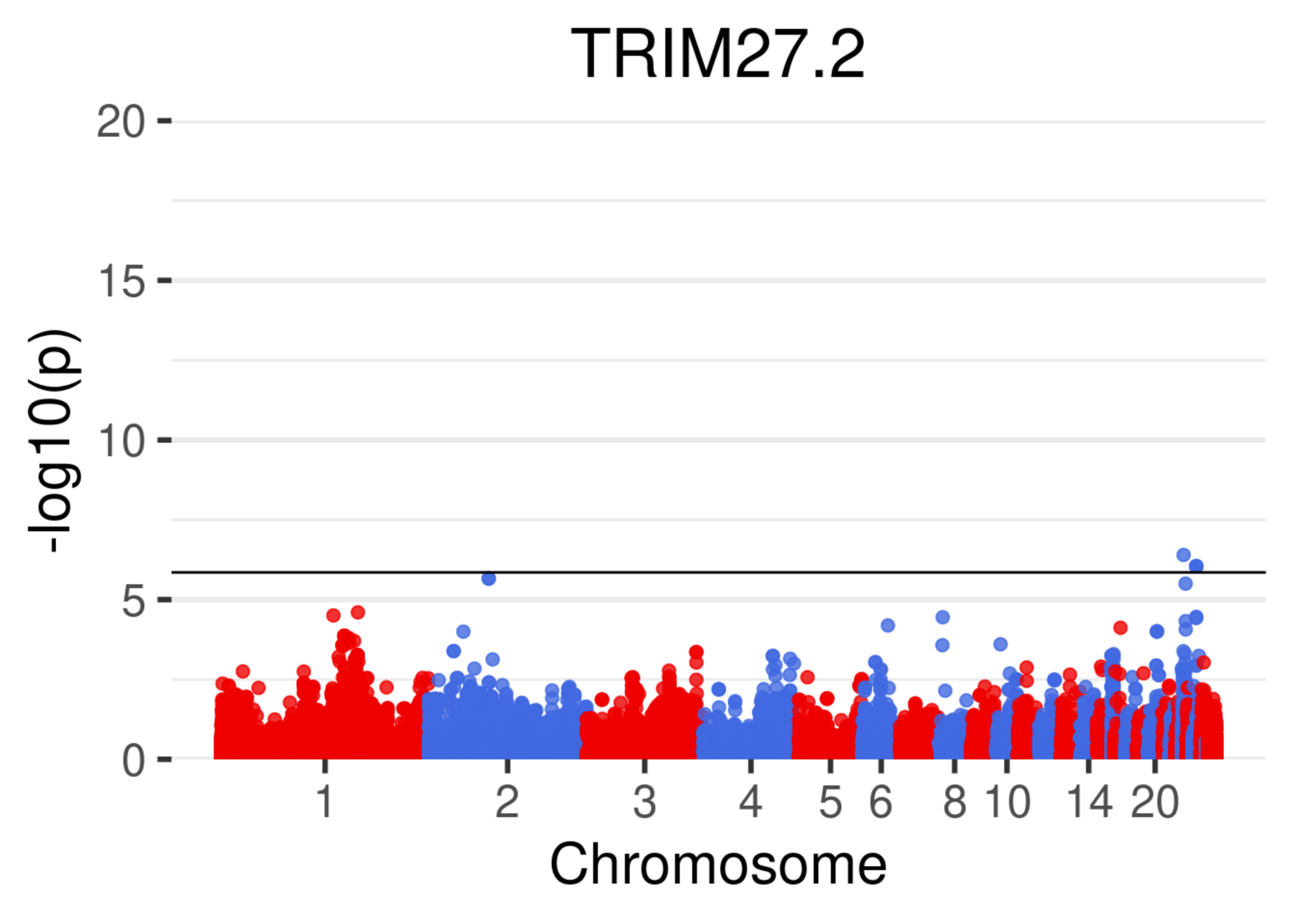

TRIM27.2
20
15
10
5
0
-log10(p)
1
2
3
4
5
6
8
10
14
20
Chromosome

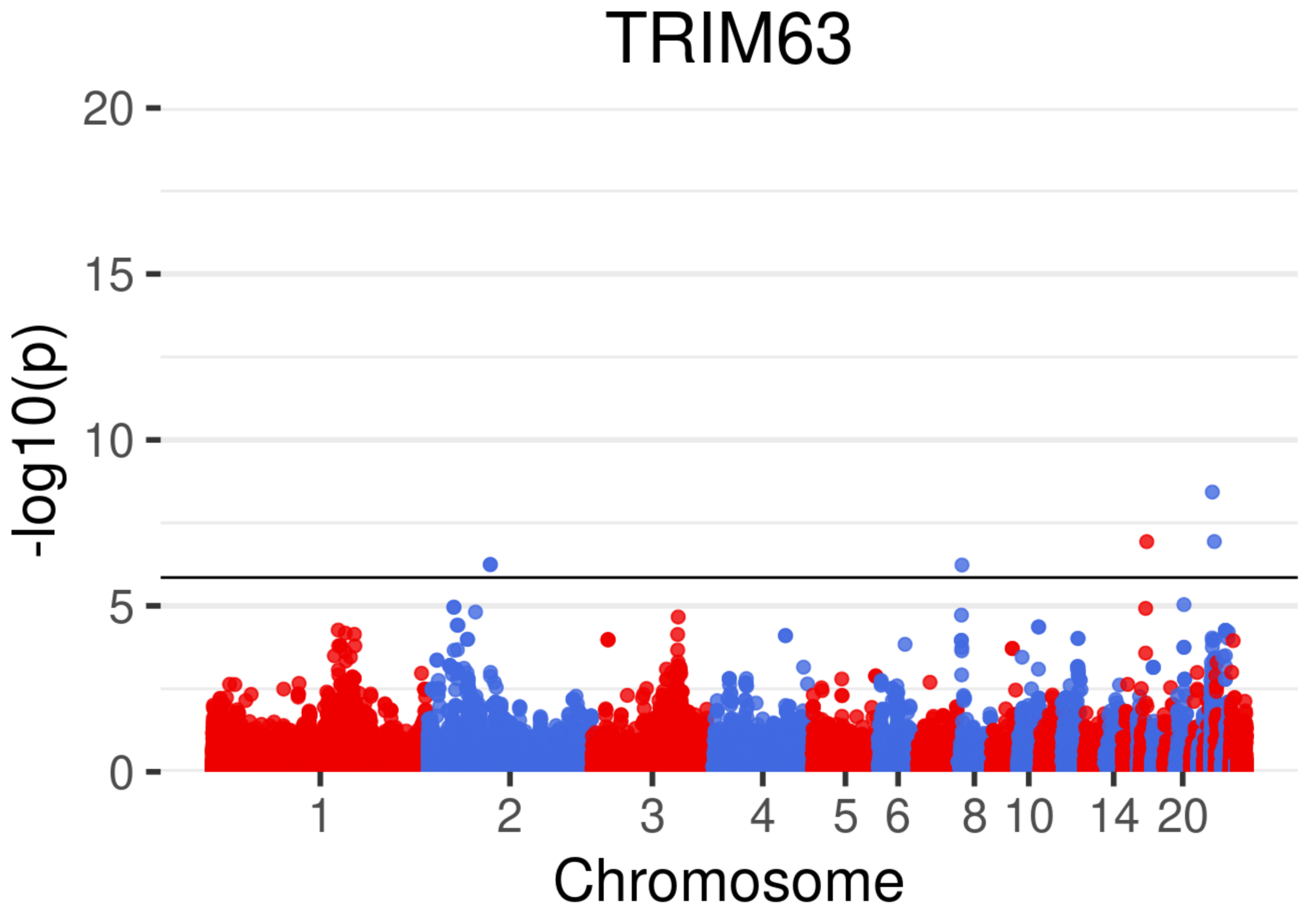

TRIM63
-log10(p)
20
15
10
5
0
1
2
3
4
5
6
8
10
14
20
Chromosome
