# Supplementary material for: Structural variation and eQTL analysis in two experimental populations of chickens divergently selected for feather-pecking behavior
Source: Neurogenetics. 2022 Nov 30;24(1):29–41. doi: 10.1007/s10048-022-00705-5 (PMC9823035; doi:10.1007/s10048-022-00705-5)

Supplementary Information S3: Graphical representation of *ETV1* binding sites in proximity to associated differentially expressed genes.

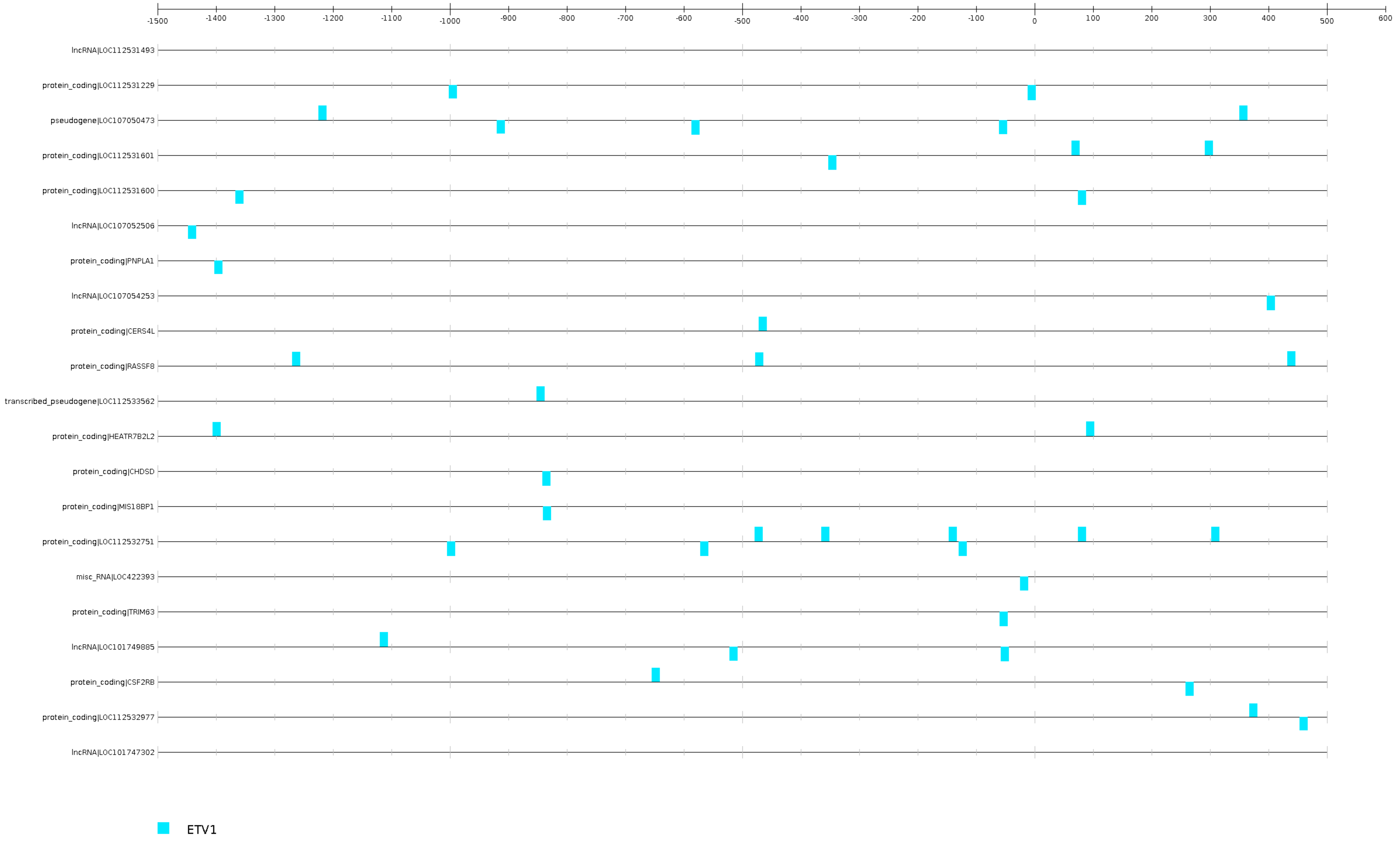

Supplement: Supplementary file 4 — Supplementary file4 (PDF 52 KB) [file 10048_2022_705_MOESM4_ESM.pdf]
